# Synthesis and Biological Evaluation of 2*H*-Indazole Derivatives: Towards Antimicrobial and Anti-Inflammatory Dual Agents

**DOI:** 10.3390/molecules22111864

**Published:** 2017-10-31

**Authors:** Jaime Pérez-Villanueva, Lilián Yépez-Mulia, Ignacio González-Sánchez, Juan Francisco Palacios-Espinosa, Olivia Soria-Arteche, Teresita del Rosario Sainz-Espuñes, Marco A. Cerbón, Karen Rodríguez-Villar, Ana Karina Rodríguez-Vicente, Miguel Cortés-Gines, Zeltzin Custodio-Galván, Dante B. Estrada-Castro

**Affiliations:** 1 Departamento de Sistemas Biológicos, División de Ciencias Biológicas y de la Salud, Universidad Autónoma Metropolitana-Xochimilco (UAM-X), Ciudad de México 04960, Mexico; jpalacios@correo.xoc.uam.mx (J.F.P.-E.); soriao@correo.xoc.uam.mx (O.S.-A.); trsainz@correo.xoc.uam.mx (T.d.R.S.-E.); qkarenrodv@hotmail.com (K.R.-V.); karovi24@hotmail.com (A.K.R.-V.); miguel_gines@outlook.com (M.C.-G.); zeltzin_888@hotmail.com (Z.C.-G.); benzaidest@hotmail.com (D.B.E.-C.); 2 Unidad de Investigación Médica en Enfermedades Infecciosas y Parasitarias-Pediatría, IMSS, Ciudad de México 06720, Mexico; lilianyepez@yahoo.com; 3 Catedrático CONACYT comisionado a Departamento de Sistemas Biológicos, División de Ciencias Biológicas y de la Salud, Universidad Autónoma Metropolitana-Xochimilco (UAM-X), Ciudad de México 04960, Mexico; ignacio.gonzalez.s@gmail.com; 4 Facultad de Química, Departamento de Biología, Universidad Nacional Autónoma de México, Ciudad de México 04510, Mexico; mcerbon85@yahoo.com.mx

**Keywords:** anticandidal, indazole, *Entamoeba histolytica*, *Giardia intestinalis*, rational drug design, *Trichomonas vaginalis*

## Abstract

Indazole is considered a very important scaffold in medicinal chemistry. It is commonly found in compounds with diverse biological activities, e.g., antimicrobial and anti-inflammatory agents. Considering that infectious diseases are associated to an inflammatory response, we designed a set of 2*H*-indazole derivatives by hybridization of cyclic systems commonly found in antimicrobial and anti-inflammatory compounds. The derivatives were synthesized and tested against selected intestinal and vaginal pathogens, including the protozoa *Giardia intestinalis*, *Entamoeba histolytica*, and *Trichomonas vaginalis*; the bacteria *Escherichia coli* and *Salmonella enterica* serovar Typhi; and the yeasts *Candida albicans* and *Candida glabrata*. Biological evaluations revealed that synthesized compounds have antiprotozoal activity and, in most cases, are more potent than the reference drug metronidazole, e.g., compound **18** is 12.8 times more active than metronidazole against *G. intestinalis*. Furthermore, two 2,3-diphenyl-2*H*-indazole derivatives (**18** and **23**) showed in vitro growth inhibition against *Candida albicans* and *Candida glabrata*. In addition to their antimicrobial activity, the anti-inflammatory potential for selected compounds was evaluated in silico and in vitro against human cyclooxygenase-2 (COX-2). The results showed that compounds **18**, **21**, **23**, and **26** display in vitro inhibitory activity against COX-2, whereas docking calculations suggest a similar binding mode as compared to rofecoxib, the crystallographic reference.

## 1. Introduction

Infectious diseases caused by protozoa, bacteria, and yeasts have a major impact on human health. Enteric pathogenic protozoa and bacteria are a frequent cause of intestinal disease which, in turn, is an important cause of morbidity and mortality around the world [1]. Two important etiological agents of intestinal parasitic diseases are the protozoa *Giardia intestinalis* and *Entamoeba histolytica*, which have been estimated to affect 280 million and 50 million people worldwide each year, respectively [2,3]. Furthermore, some bacterial strains have been identified as responsible for severe intestinal illness. Examples of these are pathogenic strains of *Escherichia coli*, e.g., enterohemorrhagic *E. coli* (EHEC) and enteroaggregative *E. coli* (EAEC), and *Salmonella enterica* serovar Typhi [1,4]. Intestinal diseases caused by protozoa and bacteria affect persons of all ages, but have a high incidence in children [1,5]. Even though infections associated with each pathogen display particular clinical symptoms, all of them are causal agents of infectious diarrhea with severe health consequences and that could lead to death [1,4,5].

On the other hand, *Trichomonas vaginalis* and *Candida albicans* are two of the major etiological agents of vaginitis. According to the World Health Organization (WHO), 276 million new cases of trichomoniasis have been estimated [6]. Infection by *T. vaginalis* can cause severe inflammation of the genital tract, which has been associated with preterm labor, low-birth weight, sterility, cervical cancer, and predisposition to HIV infection [5,6,7]. In addition, it has been reported that 75% of women have at least one vaginal yeast infection during their lifespan [8]. Infections by *Candida* usually cause swelling, itching, and irritation and can turn into a very serious problem for pregnant and immunocompromised women [8,9].

Although some antimicrobial drugs are currently available for treatment of intestinal or vaginal infections, it has been reported that resistant strains of these microbes to the current therapies are emerging and that patients’ responses to the available chemotherapeutic agents vary [5,7,8,10]. Therefore, it is important to develop new active molecules to address these current health problems.

**Figure 1:**
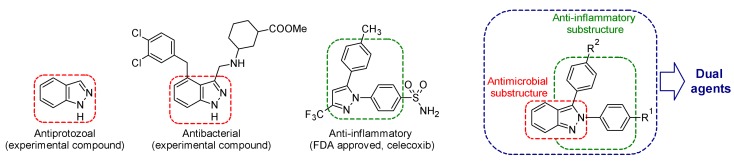
Design of the 2,3-diphenyl-2*H*-indazole derivatives.

The indazole nucleus is a very important heterocyclic framework in medicinal chemistry. This scaffold is present in a large number of compounds with a wide range of biological activities [11]. Some indazole derivatives have recently been reported as antiprotozoals, with activity against *E. histolytica* and *T. vaginalis* [12,13]. Furthermore, indazole derivatives have been synthesized and tested against several Gram-positive and Gram-negative bacterial strains [11,14,15]. Particularly, 3-phenyl-1*H*-indazole, and some derivatives, have been identified as DNA gyrase B inhibitors [16]. Although, these reports give an insight of the potential of indazole derivatives as antiprotozoal and antibacterial agents, the information available is still limited. Therefore, it is necessary to synthesize new indazole derivatives to obtain more information about their antimicrobial potential. Considering a multitarget design approach [17], the derivatives presented in this work were designed from a combination of cyclic systems found in antiprotozoal [12,13], antibacterial [11,14,15,16,18], and anti-inflammatory compounds (Figure 1) [19,20,21]. This strategy was chosen because an inflammatory response is commonly found in infectious and parasitic diseases (e.g., amebiosis and trichomonosis). Moreover, previous studies showed that amebic infections induce host cyclooxygenase-2 (COX-2) and consequently the production of prostaglandin PGE_2_. Therefore, it has been suggested that PGE_2_ could play a major role in pathogenesis of *E. histolytica* [22,23]. Eighteen compounds including 2-phenyl-2*H*-indazole and 2,3-diphenyl-2*H*-indazole derivatives were synthesized and tested against the protozoa *G. intestinalis*, *E. histolytica*, and *T. vaginalis*. The more active antiprotozoal compounds were tested against some bacterial and yeast strains including enterohemorrhagic and enteroaggregative *E. coli* (strain 933/EHEC and strain 042/EAEC), *S. enterica* serovar Typhi, *C. albicans*, and *C. glabrata*. Additionally, six selected compounds were assayed in vitro and in silico as potential anti-inflammatory agents using the COX-2 inhibition assay. To shed light on the potential toxic effects, the cytotoxicity of a selected group of compounds was assessed on HaCaT (aneuploid immortal keratinocytes) and HeLa (human epitheloid cervix carcinoma) cells.

## 2. Results and Discussion

### 2.1. Chemical Synthesis

**Scheme 1:**
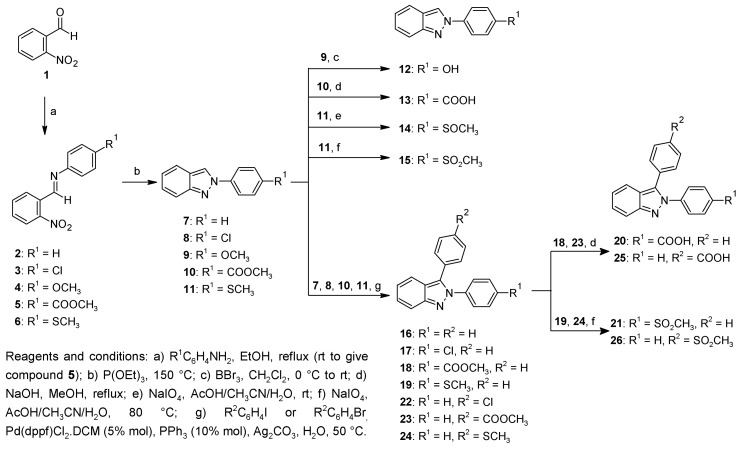
Synthesis of indazole derivatives **7**–**26**.

The 2-phenyl-2*H*-indazole and 2,3-diphenyl-2*H*-indazole derivatives (**7**–**26**) were synthesized as illustrated in Scheme 1. The commercially-available 2-nitrobenzaldehyde (**1**) was heated with aniline or *p*-substituted aniline under reflux conditions to afford the corresponding Schiff bases (**2**–**4**, **6**); only the reaction to afford compound **5** was conducted at room temperature to achieve better results. Compounds **2**–**6** were reduced and cyclocondensed with P(OEt)_3_ to give the 2-phenyl-2*H*-indazole derivatives (**7**–**11**) by the Cadogan reaction [24]. Compound **12** was synthesized by *o*-demethylation of **9** with boron tribromide [25]. Hydrolysis of **10** with NaOH afforded the carboxylic acid **13**. Compounds **14** and **15** were obtained by *S*-oxidation of **11** with sodium metaperiodate [26]. Compounds **16**–**19** and **22**–**24** were synthesized by a palladium-catalyzed arylation of the corresponding 2-phenyl-2*H*-indazole derivative with a variety of aryl iodides or bromides as previously reported [27]. Chemical yields for the palladium catalyzed arylation were slightly lower as compared to previously reported data by Ohnmacht et al. These results can be explained since the original reported methodology was scaled up tenfold to achieve the needed quantity of products for the biological assays. With the same procedure of hydrolysis and *S*-oxidation described above, compounds **20** and **25** were obtained from **18** and **23**, respectively, whereas **19** and **24** were used as starting material to produce **21** and **26**.

All synthesized compounds have sharp melting points and were characterized by using ^1^H-NMR (nuclear magnetic resonance) and ^13^C-NMR spectra. The data on previously-reported structures were consistent with literature reports. Eight of the synthetized indazole derivatives resulted in new structures, which were also characterized by high-resolution mass spectrometry. The nuclear magnetic resonance and mass spectra of compounds can be found in Figures S1–S49 in the Supporting Information.

### 2.2. Antiprotozoal Activity

The in vitro antiprotozoal assays against *E. histolytica*, *G. intestinalis*, and *T. vaginalis* of the 2-phenyl-2*H*-indazole and 2,3-diphenyl-2*H*-indazole derivatives were carried out following the method previously described [28,29]. 2-Phenyl-2*H*-indazole derivatives **7**–**15** were evaluated and the results are shown in Table 1 as IC_50_ values. Metronidazole (MTZ) and albendazole (ABZ) were used as reference drugs. The most active 2-phenyl-2*H*-indazole derivatives against the three protozoa were compounds **8** and **10**, these compounds containing 4-chlorophenyl and 4-(methoxycarbonyl)phenyl groups at position 2. Similarly, compound **7**, having a phenyl at position 2, has good activity against the three protozoa, ranking third in activity for *T. vaginalis* and forth against *G. intestinalis* and *E. histolytica*. Additionally, compound **15**, with 4-(methylsulfonyl)phenyl at the same position, can be classified among the fourth most active 2-phenyl-2*H*-indazole derivatives, at least for two parasites evaluated (*G. intestinalis* and *T. vaginalis*). Considering these results, 2,3-diphenyl-2*H*-indazole derivatives **16**, **17**, **18**, **21**, **22**, **23**, and **26** were selected to be tested for their antiprotozoal activity. These derivatives had groups H, COOCH_3_, Cl, and SO_2_CH_3_ at positions 2 and 3, substituents that induced the best response in 2-phenyl-2*H*-indazole derivatives. In addition, carboxylic acids **20** and **25** were of interest for comparative purposes with ester derivatives. Comparison of the 2,3-diphenyl-2*H*-indazole derivatives (**16**, **17**, **18**, **20**, and **21**) with its parent analogs 2-phenyl-2*H*-indazole derivatives (**7**, **8**, **10**, **13**, and **15**) indicated that only compounds **16**, **17**, and **20**, retained or increased the potency against at least two parasites; however, the improvement in activity was poor. Compound **16** increased only its potency two-fold against *G. intestinalis* and *T. vaginalis* relative to **7**; whereas compound **17** also increased its potency against *E. histolytica* two-fold when compared with **8**, whereas the activity against *G. intestinalis* and *T. vaginalis* is preserved. A similar two-fold improvement in potency was found for compound **20**, when compared to **13**, against *G. intestinalis* and *E. histolytica* and a three-fold improvement against *T. vaginalis*.

On the other hand, the influence of changing Cl, COOCH_3_, SO_2_CH_3_, and COOH substituents from the phenyl group at position 2 to the phenyl group at position 3, was studied (compounds **22**, **23**, **25**, and **26**). The results showed that activities for compounds **22**, **23**, **25**, and **26** were equal, or even lower, in most cases than their respective analogs **17**, **18**, **20**, and **21** against the three parasites evaluated. The only exception was **26**, which improved four-fold its activity against *E. histolytica* compared to **21**. Although 2,3-diphenyl-2*H*-indazole derivatives have good antiprotozoal activity, most of the compounds have equal or even lower activity than its corresponding 2-phenyl-2*H*-indazole analog. Nevertheless, all tested compounds behave as potent antiprotozoal agents, in almost all cases better than metronidazole, the drug of choice. Additionally, most compounds were slightly more potent against *E. histolytica*, compared with the other two evaluated parasites. Although, some indazole derivatives have been reported as active compounds against *E. histolytica* and *T. vaginalis*, the activity against *G. intestinalis* had not been previously reported for derivatives having an indazole nucleus. Therefore, 2-phenyl-2*H*-indazole and 2,3-diphenyl-2*H*-indazole are promising frameworks for the design of new antiprotozoal agents.

### 2.3. Antibacterial and Anticandidal Assays

The susceptibility assays against *E. coli* 933, *E. coli* 042, *S. enterica* serovar Typhi, *C. albicans*, and *C. glabrata* were carried out using the disk diffusion test, in accordance with the procedure outlined by The Clinical and Laboratory Standards Institute (CLSI) [30]. A selection of compounds based on the results from the antiprotozoal assays were tested at 5 mg/mL (Table S1), however, they were inactive or poorly active even at high concentration against the bacterial strains tested. Nevertheless, compounds **18** and **23** showed a notable inhibition zone against *C. albicans* (inhibition halos of 10 and 13 mm, respectively). Moreover, these same compounds showed activity against *C. glabrata* (inhibition halos of 3 and 4 mm, respectively), which is usually less sensitive to the commercial antimycotics. Based on these observations, the minimum inhibitory concentration (MIC) against *C. albicans* and *C. glabrata* was calculated for compounds **18** and **23**, Table 2. The results showed that both compounds display activity in the low millimolar range, and are slightly more active against *C. albicans* as compared to *C. glabrata*.

### 2.4. In Vitro and In Silico Studies on Cyclooxygenase-2

**Figure 2:**
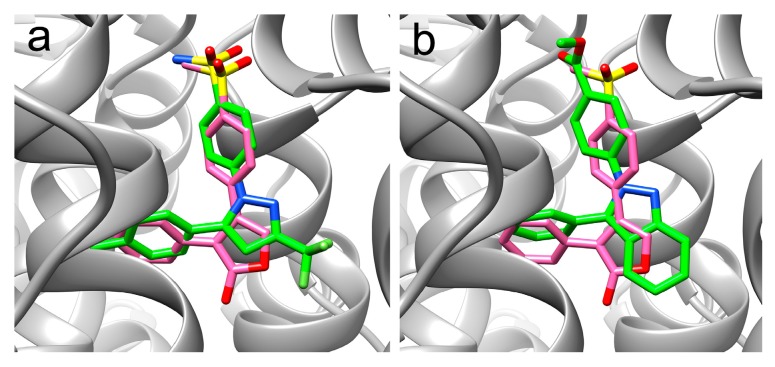
Predicted binding modes on human COX-2 for celecoxib (panel (**a**)) and **18** (panel (**b**)). Docked compounds are shown in green, whereas the crystallographic reference rofecoxib is in pink.

Considering that inflammatory response is associated with parasitic infections and the suggested role of PGE_2_, and therefore of the host COX-2 in the pathogenesis of *E. histolytica* [22], in vitro assays on human recombinant COX-2 were carried out. Additionally, molecular docking studies using AutoDock Vina software (TSRI, La Jolla, CA, USA) were performed to aid in the interpretation of the experimental results [31]. Compounds **18** and **23** were evaluated because of their strong antiprotozoal activity and their moderate anticandidal effects. Moreover, compounds **21** and **26** were of interest because of their methylsulfonyl group, which is commonly found in COX-2 inhibitors. Additionally, compounds **7** and **16** were considered as unsubstituted references. The results for in vitro assays and docking calculations are shown in Table 3. Compounds **7**, **16**, **18**, **21**, **23**, and **26** were tested at 10 µM, whereas the positive reference celecoxib was used at 1 µM, as previously described [32]. The results showed in vitro COX-2 inhibition by compounds **18**, **21**, **23**, and **26** (36–50%, at 10 µM); however, they are still weak inhibitors (see Table 3). Nevertheless, these compounds represent an interesting starting point towards the design of new antiparasitic compounds with an additional COX-2 inhibitory property. Docking studies suggest a similar binding mode of compounds **18**, **21**, **23**, and **26** against human COX-2 as compared to celecoxib and the crystallographic ligand rofecoxib [33]. The predicted binding mode of the reference celecoxib and **18** are shown in Figure 2. Additionally, better docking scores were found for the 2,3-diphenyl-2*H*-indazole derivatives **16**, **18**, **21**, **23**, and **26** as compared to the 2*H*-indazole derivatives (e.g., compound **7**).

### 2.5. Cytotoxicity Assays

Biological assays on HaCaT and HeLa cell lines were conducted to gain insight into the cytotoxic effects of these derivatives on human cells, as compared with the effects observed on protozoa. Cellular viability was determined by MTT (3-(4,5-dimethylthiazol-2-yl)-2,5-diphenyltetrazolium bromide) assay [34,35]. Compounds **18** and **23** were chosen considering their antiprotozoal and anticandidal effect, in addition to their COX-2 inhibitory activity. Additionally, compound **16** was considered as an unsubstituted reference. The results of the cytotoxicity assays are shown in Table 4.

Although the IC_50_ determinations were limited by the low solubility of the compounds in the cell culture medium at higher concentrations than 10 µM, they did not exhibit important cytotoxic effect at 10 µM in either of the cell lines (% viability > 90%). Therefore, the IC_50_ values are higher than 10 µM in all cases. Since all tested compounds showed antiprotozoal activity (IC_50_ values) lower than one micromolar (high nanomolar range), the results indicated that compounds **16**, **18**, and **23** are selective antiprotozoal compounds. Only compound **16** was soluble enough for IC_50_ determination on HaCaT and HeLa cell lines, having values of 93.65 and 125.00 µM, respectively.

In summary, nine 2-phenyl-2*H*-indazole derivatives (**7**–**15**) and eleven 2,3-diphenyl-2*H*-indazole derivatives (**16**–**26**) were synthesized. Eight compounds resulted in new structures (**14**, **15**, **18**–**21**, **25**, and **26**). Biological evaluations revealed that 2-phenyl-2*H*-indazole and 2,3-diphenyl-2*H*-indazole derivatives have giardicidal, amebicidal, and trichomonicidal activity lower than one micromolar and, in most cases, are more potent than the drug of choice metronidazole. Although the compounds are mainly inactive against the used bacterial strains, a major finding was that most of the compounds are selective antiprotozoal agents. In addition, compounds **18** and **23** inhibit in vitro growth of *C. albicans* and *C. glabrata*. Furthermore, compounds **18**, **21**, **23**, and **26** showed inhibition of COX-2 at 10 µM, which adds an interesting property to these 2,3-diphenyl-2*H*-indazole derivatives, since COX-2 inhibition has been suggested to be beneficial on *E. histolytica* infections. Assays in HaCaT and HeLa cells revealed low cytotoxicity in human cells for a selection of these derivatives. These results suggest that 2-phenyl-2*H*-indazole and 2,3-diphenyl-2*H*-indazole are promising scaffolds for the design of new compounds against intestinal and vaginal pathogens, such as protozoa and yeasts. The mechanisms of action of indazole derivatives synthesized in this work as antiprotozoal and anticandidal agents are still unknown and constitutes a further research topic to be addressed in future research.

## 3. Materials and Methods

### 3.1. Chemicals and Instruments

All chemicals and starting materials were obtained from Sigma-Aldrich (Toluca, MC, Mexico). Reactions were monitored by TLC on 0.2 mm percolated silica gel 60 F254 plates (Merck, Darmstadt, Germany) and visualized by irradiation with a UV lamp. Silica gel 60 (70–230 mesh) was used for column chromatography. Melting points were determined in open capillary tubes with a Büchi M-565 melting point apparatus (Flawil, Switzerland) and are uncorrected. ^1^H-NMR and ^13^C-NMR spectra were measured with an Agilent DD2 spectrometer (Santa Clara, CA, USA), operating at 600 MHz and 151 MHz for ^1^H and ^13^C, respectively. Chemical shifts are given in parts per million relative to tetramethylsilane (Me_4_Si, *δ* = 0); *J* values are given in Hz. Splitting patterns are expressed as follow: s, singlet; d, doublet; q, quartet; dd, doublet of doublet; t, triplet; m, multiplet; bs, broad singlet. High-resolution mass spectra were recorded on a Bruker ESI/APCI-TOF, MicroTOF-II-Focus spectrometer (Billerica, MA, USA) by electrospray ionization (ESI). All compounds were named using the automatic name generator tool implemented in ChemBioDraw Ultra 13.0 software (PerkinElmer, Waltham, MA, USA), according IUPAC rules.

### 3.2. Chemical Synthesis

*General procedure for the synthesis of 1-(2-nitrophenyl)-N-phenylmethanimines* (**2**–**6**). 2-Nitrobenzaldehyde (5 g, 33.08 mmol) and aniline or the corresponding substituted aniline (33.08 mmol, 1 eq) were dissolved in ethanol (12–40 mL; the minimum quantity to dissolve the starting materials) and stirred at reflux temperature for 1–4 h to yield compounds **2**–**4**, **6**. Finally, the mixture was cooled to induce crystallization and the solid formed was separated using vacuum filtration and washed with cold ethanol. This same reaction was carried out at room temperature to yield compound **5**.

*General procedure for the synthesis of 2-phenyl-2H-indazole derivatives* (**7**–**11**). 2-Phenyl-2*H*-indazole derivatives were synthesized employing a slight modification of the Cadogan method [24]. The corresponding imine **2**–**6** (20 mmol) was heated in triethyl phosphite (60 mmol) at 150 °C (0.5–2 h) until the starting material was totally consumed. Then, phosphite and phosphate were separated using vacuum distillation and the residue was purified using column chromatography with hexane–ethyl acetate (90:10) as a mobile phase to give the respective 2-phenyl-2*H*-indazole derivatives **7**–**9** and **11**. A slightly more polar mobile phase was used for the purification of the compound **10**, hexane-ethyl acetate (80:20).

*4-(2H-indazol-2-yl) phenol* (**12**). Compound **9** (4 mmol) was dissolved in dichloromethane (12 mL) and cooled to 0 °C under N_2_ atmosphere. Then, boron tribromide (12 mL of 1 M solution in dichloromethane, 12 mmol) was added and the reaction mixture was warmed to room temperature and stirred overnight. After completion of the reaction, a saturated sodium bicarbonate solution was added and the solid formed was filtered under vacuum. The crude product was purified using a short column packed with silica gel and ethyl acetate-hexanes (6:4) as a mobile phase to give compound **12**.

*General procedure for the synthesis of derivatives* **13**, **20***, and* **25**. The appropriate methyl ester derivative (**10**, **18**, and **23**, 1.2 mmol) was dissolved in methanol (7.5 mL) and an aqueous solution of NaOH (3.6 mmol in 3 mL of water) was added. The reaction mixture was heated under reflux for five hours. After completion of the reaction, the mixture was cooled on ice and acidified to pH 1 with HCl to induce precipitation. The solid was separated using vacuum filtration and dried.

*2-(4-(Methylsulfinyl) phenyl)-2H-indazole* (**14**). To a solution of compound **11** (0.8 mmol) in 28 mL of CH_3_CN/CH_3_COOH (1:1), NaIO_4_ (0.8 mmol) dissolved in 2 mL of H_2_O/AcOH (4:1) was added. The reaction mixture was stirred at room temperature for 24 h. Then, the reaction was neutralized with a saturated solution of sodium bicarbonate and the product was extracted with dichloromethane (3 × 50 mL). The organic phase was dried with anhydrous sodium sulfate and concentrated under vacuum. The evaporation residue was purified by column chromatography using dichloromethane/methanol (98:2) as a mobile phase to give compound **14**.

*General procedure for the synthesis of derivatives* **15**, **21**, and **26**. NaIO_4_ (5 mmol) dissolved in 5 mL of H_2_O/AcOH (4:1) were added to a solution of the proper indazole derivative **11**, **19**, or **24** (2 mmol) in 28 mL of CH_3_CN/CH_3_COOH (1:1). The reaction mixture was stirred at reflux temperature for 12 h. Then, the mixture was neutralized with a saturated solution of sodium bicarbonate and brine solution was added until complete precipitation. The solid was separated using vacuum filtration and dried. The crude product was purified by column chromatography using dichloromethane as a mobile phase.

*General procedure for the synthesis of 2,3-diphenyl-2H-indazole derivatives* **16**–**19** and **22**–**24**. Compounds **16**–**19** and **22**–**24** were synthesized by a palladium catalyzed arylation as previously described by Ohnmacht et al. [27]. It is worth mentioning that the previously-reported methodology was scaled up to 0.5 g of starting 2-phenyl-2*H*-indazole. Whereas compounds **16**–**19**, **22**, and **23**, were synthesized using the proper 2-phenyl-2*H*-indazole and the substituted 4-iodobenzene, only compound **24** was synthesized from 2-phenyl-2*H*-indazole and 4-bromothioanisole.

*1-(2-Nitrophenyl)-N-phenylmethanimine* (**2**). Yellow solid (93% yield); m.p.: 64.1–64.9 °C (lit [24]: 63–64 °C); ^1^H-NMR (600 MHz, CDCl_3_) δ 8.94 (s, 1H), 8.31 (dd, *J* = 7.8, 1.4 Hz, 1H), 8.07 (dd, *J* = 8.2, 1.1 Hz, 1H), 7.74 (t, *J* = 7.6 Hz, 1H), 7.64–7.60 (m, 1H), 7.45–7.40 (m, 2H), 7.31–7.27 (m, 3H); ^13^C-NMR (151 MHz, CDCl_3_) δ 155.84, 151.07, 149.34, 133.58, 131.18, 131.12, 129.75, 129.28, 126.92, 124.54, 121.18.

*N-(4-Chlorophenyl)-1-(2-nitrophenyl) methanimine* (**3**). Dark yellow solid (91% yield); m.p.: 91.2–92.2 °C (lit [36]: 91–92 °C). ^1^H-NMR (600 MHz, CDCl_3_) δ 8.93 (s, 1H), 8.29 (dd, *J* = 7.8, 1.5 Hz, 1H), 8.08 (dd, *J* = 8.2, 1.2 Hz, 1H), 7.78–7.72 (m, 1H), 7.67–7.61 (m, 1H), 7.41–7.36 (m, 2H), 7.25–7.20 (m, 2H); ^13^C-NMR (151 MHz, CDCl_3_) δ 156.24, 149.49, 149.32, 133.64, 132.58, 131.40, 130.87, 129.72, 129.40, 124.61, 122.54.

*N-(4-Methoxyphenyl)-1-(2-nitrophenyl) methanimine* (**4**). Yellow solid (92% yield); m.p.: 79.1–79.9 °C (lit [36]: 81–82 °C); ^1^H-NMR (600 MHz, CDCl_3_) δ 8.97 (s, 1H), 8.32 (dd, *J* = 7.8, 1.4 Hz, 1H), 8.06 (dd, *J* = 8.2, 1.1 Hz, 1H), 7.75–7.70 (m, 1H), 7.62–7.57 (m, 1H), 7.35–7.29 (m, 2H), 6.98–6.94 (m, 2H), 3.85 (s, 3H); ^13^C-NMR (151 MHz, CDCl_3_) δ 159.09, 153.31, 143.88, 133.48, 131.36, 130.81, 129.55, 124.53, 122.78, 114.50, 55.53.

*Methyl 4-((2-nitrobenzylidene) amino)benzoate* (**5**) Pale yellow solid (73% yield); m.p.: 122.7–124.4 °C; ^1^H-NMR (600 MHz, CDCl_3_) δ 8.93 (s, 1H), 8.30 (dd, *J* = 7.7, 1.0 Hz, 1H), 8.10 (d, *J* = 8.4 Hz, 3H), 7.76 (t, *J* = 7.6 Hz, 1H), 7.68–7.63 (m, 1H), 7.30–7.25 (m, 2H), 3.93 (s, 3H); ^13^C-NMR (151 MHz, CDCl_3_) δ 166.66, 157.50, 155.14, 149.39, 133.71, 131.66, 130.95, 130.70, 129.84, 128.26, 124.65, 120.93, 52.15; MS (HR-ESI) for C_15_H_12_N_2_O_4_ [M + H]^+^, calcd: *m*/*z* 285.0870, found: *m*/*z* 285.0861.

*N-(4-(Methylthio)phenyl)-1-(2-nitrophenyl)methanimine* (**6**). Burnt orange solid (92% yield); m.p.: 69.3–70.4 °C; ^1^H-NMR (600 MHz, CDCl_3_) δ 8.96 (s, 1H), 8.31 (dd, *J* = 7.8, 1.4 Hz, 1H), 8.07 (dd, *J* = 8.2, 1.1 Hz, 1H), 7.73 (t, *J* = 7.5 Hz, 1H), 7.63–7.58 (m, 1H), 7.33–7.22 (m, 4H), 2.52 (s, 3H); ^13^C-NMR (151 MHz, CDCl_3_) δ 154.86, 149.25, 148.06, 137.45, 133.51, 131.07, 129.63, 127.37, 124.53, 121.89, 16.06; MS (HR-ESI) for C_14_H_12_N_2_O_2_S [M + H]^+^, calcd: *m*/*z* 273.0692, found: *m*/*z* 273.0683.

*2-Phenyl-2H-indazole* (**7**). White solid (64% yield); m.p.: 81.2–81.6 °C (lit [24]: 81–82 °C); the spectroscopic data matched previously reported data [37]: ^1^H-NMR (600 MHz, CDCl_3_) δ 8.40 (d, *J* = 0.9 Hz, 1H), 7.91–7.88 (m, 2H), 7.79 (dd, *J* = 8.8, 0.9 Hz, 1H), 7.70 (dt, *J* = 8.5, 1.0 Hz, 1H), 7.54–7.50 (m, 2H), 7.41–7.37 (m, 1H), 7.32 (ddd, *J* = 8.8, 6.6, 1.0 Hz, 1H), 7.11 (ddd, *J* = 8.4, 6.6, 0.7 Hz, 1H); ^13^C-NMR (151 MHz, CDCl_3_) δ (ppm): 149.78, 140.52, 129.54, 127.88, 126.81, 122.76, 122.44, 120.99, 120.39, 120.37, 117.94.

*2-(4-Chlorophenyl)-2H-indazole* (**8**). White solid (57% yield); m.p.: 143.0–145.5 °C (lit [38]: 138–140 °C); the spectroscopic data matched previously reported data [38]: ^1^H-NMR (600 MHz, CDCl_3_) δ 8.37 (d, *J* = 1.0 Hz, 1H), 7.87–7.82 (m, 2H), 7.77 (dq, *J* = 8.8, 0.9 Hz, 1H), 7.69 (dt, *J* = 8.5, 1.0 Hz, 1H), 7.51–7.47 (m, 2H), 7.33 (ddd, *J* = 8.8, 6.6, 1.1 Hz, 1H), 7.12 (ddd, *J* = 8.5, 6.6, 0.8 Hz, 1H); ^13^C-NMR (151 MHz, CDCl_3_) δ 149.89, 139.02, 133.55, 129.67, 127.09, 122.87, 122.71, 122.00, 120.29, 117.90.

*2-(4-Methoxyphenyl)-2H-indazole* (**9**). Beige solid (56 % yield); m.p.: 133.2–135.8 °C (lit [39]: 130–131 °C); the spectroscopic data matched previously reported data [40]: ^1^H-NMR (600 MHz, CDCl_3_) δ 8.30 (d, *J* = 0.9 Hz, 1H), 7.82–7.76 (m, 3H), 7.69 (dt, *J* = 8.4, 1.0 Hz, 1H), 7.31 (ddd, *J* = 8.7, 6.6, 1.0 Hz, 1H), 7.10 (ddd, *J* = 8.4, 6.6, 0.8 Hz, 1H), 7.05–6.99 (m, 2H), 3.86 (s, 3H); ^13^C-NMR (151 MHz, CDCl_3_) δ 159.28, 149.58, 134.12, 126.53, 122.70, 122.41, 122.22, 120.30, 120.25, 117.77, 114.63, 55.60.

*Methyl 4-(2H-indazol-2-yl) benzoate* (**10**). White solid (52% yield); m.p.: 185.8–186.2 °C (lit [41]: 186–187 °C); the spectroscopic data matched previously reported data [40]: ^1^H-NMR (600 MHz, CDCl_3_) δ 8.47 (d, *J* = 0.7 Hz, 1H), 8.22–8.18 (m, 2H), 8.02–7.99 (m, 2H), 7.77 (dd, *J* = 8.8, 0.8 Hz, 1H), 7.69 (d, *J* = 8.5 Hz, 1H), 7.33 (ddd, *J* = 8.8, 6.6, 1.0 Hz, 1H), 7.14–7.10 (m, 1H), 3.95 (s, 3H); ^13^C-NMR (151 MHz, CDCl_3_) δ 166.19, 150.19, 143.64, 131.16, 129.27, 127.45, 123.01, 122.98, 120.47, 120.26, 118.06, 52.33.

*2-(4-(Methylthio) phenyl)-2H-indazole* (**11**). Pale yellow solid (61% yield); m.p.: 148.3–149.7 °C (lit [38]: 137–139 °C); the spectroscopic data matched previously reported data [38]: ^1^H-NMR (600 MHz, CDCl_3_) δ 8.35 (d, *J* = 0.8 Hz, 1H), 7.84–7.80 (m, 2H), 7.79–7.76 (m, 1H), 7.68 (dt, *J* = 8.5, 0.9 Hz, 1H), 7.39–7.35 (m, 2H), 7.31 (ddd, *J* = 8.7, 6.6, 1.0 Hz, 1H), 7.10 (ddd, *J* = 8.4, 6.6, 0.8 Hz, 1H), 2.53 (s, 3H); ^13^C-NMR (151 MHz, CDCl_3_) δ 149.72, 138.63, 137.78, 127.27, 126.82, 122.77, 122.46, 121.26, 120.30, 120.12, 117.84, 15.88.

*4-(2H-Indazol-2-yl) phenol* (**12**). Beige solid (64% yield); m.p.: 179–181 °C (lit [25]: 193–194 °C); the spectroscopic data matched previously reported data [42]: ^1^H-NMR (600 MHz, DMSO-*d*_6_) δ 9.85 (s, 1H), 8.91 (d, *J* = 0.9 Hz, 1H), 7.91–7.84 (m, 2H), 7.75 (dt, *J* = 8.4, 1.0 Hz, 1H), 7.69 (dq, *J* = 8.8, 0.9 Hz, 1H), 7.29 (ddd, *J* = 8.7, 6.6, 1.1 Hz, 1H), 7.08 (ddd, *J* = 8.3, 6.6, 0.8 Hz, 1H), 6.98–6.92 (m, 2H); ^13^C-NMR (151 MHz, DMSO-*d*_6_) δ 157.09, 148.47, 132.11, 126.10, 122.24, 121.75, 121.57, 120.78, 120.58, 117.12, 115.81.

*4-(2H-Indazol-2-yl) benzoic acid* (**13**). White solid (96% yield); m.p.: 288.3–288.5 °C (lit [41]: 286–288 °C); ^1^H-NMR (600 MHz, DMSO-*d*_6_) δ 9.23 (s, 1H), 8.29–8.23 (m, 2H), 8.18–8.12 (m, 2H), 7.79 (dt, *J* = 8.5, 1.0 Hz, 1H), 7.73 (dq, *J* = 8.8, 0.9 Hz, 2H), 7.35 (ddd, *J* = 8.8, 6.5, 1.1 Hz, 1H), 7.13 (ddd, *J* = 8.5, 6.6, 0.8 Hz, 1H); ^13^C-NMR (151 MHz, DMSO-*d*_6_) δ 166.46, 149.22, 142.83, 130.82, 129.65, 127.28, 122.54, 122.43, 122.04, 120.99, 119.86, 117.48.

*2-(4-(Methylsulfinyl) phenyl)-2H-indazole* (**14**). White solid (92% yield); m.p.: 150.1–152.7 °C; ^1^H-NMR (600 MHz, CDCl_3_) δ 8.47 (d, *J* = 0.9 Hz, 1H), 8.13–8.07 (m, 2H), 7.83–7.75 (m, 3H), 7.70 (dt, *J* = 8.5, 1.0 Hz, 1H), 7.34 (ddd, *J* = 8.8, 6.6, 1.1 Hz, 1H), 7.13 (ddd, *J* = 8.5, 6.6, 0.8 Hz, 1H), 2.78 (s, 3H); ^13^C-NMR (151 MHz, CDCl_3_) δ 150.14, 145.05, 142.47, 127.45, 125.03, 123.01, 121.49, 120.46, 120.43, 118.01, 44.10; MS (HR-ESI) for C_14_H_12_N_2_OS [M + Na]^+^, calcd: *m*/*z* 279.0562, found: *m*/*z* 279.0481.

*2-(4-(Methylsulfonyl) phenyl)-2H-indazole* (**15**). White solid (68% yield); m.p.: 200.6–201.5 °C; ^1^H-NMR (600 MHz, CDCl_3_) δ 8.50 (d, *J* = 0.8 Hz, 1H), 8.19–8.05 (m, 4H), 7.76 (m, 1H), 7.70 (m, 1H), 7.35 (ddd, *J* = 8.8, 6.6, 1.0 Hz, 1H), 7.14 (ddd, *J* = 8.5, 6.6, 0.7 Hz, 1H), 3.11 (s, 3H); ^13^C-NMR (151 MHz, CDCl_3_) δ 150.43, 144.23, 139.27, 129.18, 127.87, 123.36, 123.18, 120.99, 120.57, 120.54, 118.11, 44.62; MS (HR-ESI) for C_14_H_12_N_2_O_2_S [M + H]^+^, calcd: *m*/*z* 273.0692, found: *m*/*z* 273.0659.

*2,3-Diphenyl-2H-indazole* (**16**). White solid (77% yield); mp: 107.4–107.9 °C (lit [27]: 102–103 °C); ^1^H-NMR (600 MHz, CDCl_3_) δ 7.82–7.79 (m, 1H), 7.73–7.70 (m, 1H), 7.45–7.42 (m, 2H), 7.41–7.34 (m, 9H), 7.14 (ddd, *J* = 8.4, 6.6, 0.8 Hz, 1H); ^13^C-NMR (151 MHz, CDCl_3_) δ 148.99, 140.24, 135.41, 129.91, 129.69, 128.97, 128.76, 128.30, 128.25, 126.98, 126.02, 122.50, 121.74, 120.52, 117.76.

*2-(4-Chlorophenyl)-3-phenyl-2H-indazole* (**17**). White solid (45% yield); m.p.: 124.4–125.0 °C (lit [43]: 126 °C); ^1^H-NMR (600 MHz, CDCl_3_) δ 7.78 (dt, *J* = 8.8, 0.9 Hz, 1H), 7.68–7.69 (dt, *J* = 8.5, 0.9 Hz, 1H), 7.45–7.32 (m, 10H), 7.14 (ddd, *J* = 8.4, 6.6, 0.8 Hz, 1H); ^13^C-NMR (151 MHz, CDCl_3_) δ 149.12, 138.75, 135.47, 134.09, 129.67, 129.63, 129.18, 128.94, 128.55, 127.26, 127.10, 122.73, 121.86, 120.49, 117.72; MS (HR-ESI) for C_19_H_13_ClN_2_ [M + H]^+^, calcd: *m*/*z* 305.0840, found: *m*/*z* 305.0736.

*Methyl 4-(3-phenyl-2H-indazol-2-yl) benzoate* (**18**). Pale yellow solid (40% yield); m.p.: 152.4–154.9 °C; ^1^H-NMR (600 MHz, CDCl_3_) δ 8.07–8.04 (m, 2H), 7.80 (dt, *J* = 8.8, 0.8 Hz, 1H), 7.69 (dt, *J* = 8.6, 1.0 Hz, 1H), 7.55–7.52 (m, 2H), 7.44–7.34 (m, 6H), 7.15 (ddd, *J* = 8.5, 6.6, 0.8 Hz, 1H), 3.93 (s, 3H); ^13^C-NMR (151 MHz, CDCl_3_) δ 166.21, 149.34, 143.76, 135.69, 130.42, 129.70, 129.62, 128.98, 128.66, 127.46, 125.69, 122.89, 122.08, 120.53, 117.81, 52.33; MS (HR-ESI) for C_21_H_16_N_2_O_2_ [M + H]^+^, calcd: *m*/*z* 329.1285, found: *m*/*z* 329.1103.

*2-(4-(Methylthio) phenyl)-3-phenyl-2H-indazole* (**19**). Pale yellow solid (71% yield) m.p.: 87.7–89.0 °C; ^1^H-NMR (600 MHz, CDCl_3_) δ 7.79 (dt, *J* = 8.9, 1.0 Hz, 1H), 7.70 (dt, *J* = 8.6, 1.0 Hz, 1H), 7.43–7.34 (m, 8H), 7.24–7.21 (m, 2H), 7.13 (ddd, *J* = 8.4, 6.6, 0.8 Hz, 1H), 2.49 (s, 3H); ^13^C-NMR (151 MHz, CDCl_3_) δ 148.97, 139.16, 137.23, 135.26, 129.88, 129.68, 128.83, 128.35, 127.00, 126.40, 126.19, 122.50, 121.78, 120.46, 117.68, 15.58; MS (HR-ESI) for C_20_H_16_N_2_S [M + H]^+^, calcd: *m*/*z* 317.1107, found: *m*/*z* 317.1108.

*4-(3-Phenyl-2H-indazol-2-yl) benzoic acid* (**20**). White solid (70% yield); m.p.: 129.2–130.1 °C; ^1^H-NMR (600 MHz, DMSO-*d*_6_) δ 8.04–7.99 (m, 2H), 7.77 (d, *J* = 8.8 Hz, 1H), 7.69 (d, *J* = 8.5 Hz, 1H), 7.59–7.55 (m, 2H), 7.51–7.37 (m, 6H), 7.18 (dd, *J* = 8.4, 6.6 Hz, 1H); ^13^C-NMR (151 MHz, DMSO-*d*_6_) δ 166.41, 148.44, 143.00, 135.18, 130.45, 130.07, 129.44, 128.95, 128.87, 128.63, 127.18, 125.91, 122.73, 121.30, 120.32, 117.41; MS (HR-ESI) for C_20_H_14_N_2_O_2_ [M + H]^+^, calcd: *m*/*z* 315.1128, found: *m*/*z* 315.1142.

*2-(4-(Methylsulfonyl) phenyl)-3-phenyl-2H-indazole* (**21**). Pale yellow solid (77% yield), m.p.: 101.8–102.7 °C; ^1^H-NMR (600 MHz, CDCl_3_) δ 7.98–7.94 (m, 2H), 7.78 (dt, *J* = 8.9, 0.8 Hz, 1H), 7.70–7.66 (m, 3H), 7.48–7.42 (m, 3H), 7.39 (ddd, *J* = 8.8, 6.5, 1.0 Hz, 1H), 7.38–7.35 (m, 2H), 7.16 (ddd, *J* = 8.4, 6.5, 0.7 Hz, 1H), 3.08 (s, 3H); ^13^C-NMR (151 MHz, CDCl_3_) δ 149.58, 144.52, 139.70, 135.94, 129.70, 129.28, 129.23, 129.00, 128.41, 127.85, 126.45, 123.23, 122.29, 120.58, 117.81, 44.52; MS (HR-ESI) for C_20_H_16_N_2_O_2_S [M + H]^+^, calcd: *m*/*z* 349.1005, found: *m*/*z* 349.1005.

*3-(4-Chlorophenyl)-2-phenyl-2H-indazole* (**22**). White solid (67% yield); m.p.: 141.1–142.8 °C (lit [27]: 134–135 °C); the spectroscopic data matched previously reported data [27,44]: ^1^H-NMR (600 MHz, CDCl_3_) δ 7.80 (dt, *J* = 8.8, 0.8 Hz, 1H), 7.67 (dt, *J* = 8.6, 1.0 Hz, 1H), 7.44–7.35 (m, 8H), 7.30–7.27 (m, 2H), 7.16 (ddd, *J* = 8.4, 6.5, 0.7 Hz, 1H); ^13^C-NMR (151 MHz, CDCl_3_) δ 149.00, 139.98, 134.45, 134.08, 130.84, 129.14, 129.12, 128.48, 128.38, 127.08, 126.01, 122.86, 121.71, 120.11, 117.91.

*Methyl 4-(2-phenyl-2H-indazol-3-yl) benzoate* (**23**). Pale yellow solid (76% yield): m.p.: 164.5–166.3 °C; the spectroscopic data matched previously reported data [45]: ^1^H-NMR (600 MHz, CDCl_3_) δ 8.08–8.04 (m, 2H), 7.84–7.80 (m, 1H), 7.72 (dt, *J* = 8.5, 0.9 Hz, 1H), 7.45–7.37 (m, 8H), 7.19 (ddd, *J* = 8.5, 6.5, 0.6 Hz, 1H), 3.93 (s, 3H); ^13^C-NMR (151 MHz, CDCl_3_) δ 166.55, 149.08, 139.99, 134.37, 134.13, 129.97, 129.66, 129.49, 129.18, 128.59, 127.14, 126.04, 123.18, 121.90, 120.09, 118.02, 52.29.

*3-(4-(Methylthio) phenyl)-2-phenyl-2H-indazole* (**24**). White solid, (36% yield); m.p.: 119.3–121.4 °C; the spectroscopic data matched previously reported data [45]: ^1^H-NMR (600 MHz, CDCl_3_) δ 7.79 (dt, *J* = 8.8, 0.9 Hz, 1H), 7.70 (dt, *J* = 8.5, 1.0 Hz, 1H), 7.46–7.43 (m, 2H), 7.42–7.34 (m, 4H), 7.29–7.23 (m, 4H), 7.14 (ddd, *J* = 8.5, 6.6, 0.8 Hz, 1H), 2.50 (s, 3H); ^13^C-NMR (151 MHz, CDCl_3_) δ 149.01, 140.22, 139.32, 134.94, 129.90, 129.06, 128.29, 126.99, 126.27, 126.16, 126.02, 122.50, 121.66, 120.43, 117.80, 15.26.

*4-(2-Phenyl-2H-indazol-3-yl) benzoic acid* (**25**). White solid (87% yield); mp: 296.2–298.2 °C; ^1^H-NMR (600 MHz, DMSO-*d*_6_) δ 7.94–7.90 (m, 2H), 7.75 (d, *J* = 8.7 Hz, 1H), 7.71 (d, *J* = 8.5 Hz, 1H), 7.49–7.42 (m, 5H), 7.38 (ddd, *J* = 8.7, 6.6, 0.9 Hz, 1H), 7.30–7.26 (m, 2H), 7.16 (ddd, *J* = 8.4, 6.6, 0.6 Hz, 1H); ^13^C-NMR (151 MHz, DMSO-*d*_6_) δ 169.13, 148.11, 140.15, 139.79, 135.10, 129.32, 129.17, 128.98, 128.37, 128.26, 126.77, 125.88, 122.34, 121.02, 120.38, 117.29; MS (HR-ESI) for C_20_H_14_N_2_O_2_ [M + H]^+^, calcd: *m*/*z* 315.1128, found: *m*/*z* 315.1139.

*3-(4-(Methylsulfonyl) phenyl)-2-phenyl-2H-indazole* (**26**). Pale yellow solid (60% yield), mp: 206.9–208.8 °C; ^1^H-NMR (600 MHz, CDCl_3_) δ 7.98–7.94 (m, 2H), 7.84 (dt, *J* = 8.7, 0.9 Hz, 1H), 7.71 (dt, *J* = 8.5, 1.0 Hz, 1H), 7.57–7.54 (m, 2H), 7.45–7.39 (m, 6H), 7.22 (ddd, *J* = 8.5, 6.6, 0.9 Hz, 1H), 3.11 (s, 3H); ^13^C-NMR (151 MHz, CDCl_3_) δ 149.10, 139.83, 139.71, 135.46, 132.95, 130.24, 129.40, 128.90, 127.86, 127.27, 126.06, 123.70, 122.03, 119.64, 118.22, 44.42; MS (HR-ESI) for C_20_H_16_N_2_O_2_S [M + H]^+^, calcd: *m*/*z* 349.1005, found: *m*/*z* 349.1005.

### 3.3. Biological Assays

#### 3.3.1. Antiprotozoal Activity Assays

*Trichomonas vaginalis* strain GT3, *Giardia intestinalis* isolate IMSS:0981:1, and *Entamoeba histolytica* strain HM1-IMSS were used. Trophozoites of *G. intestinalis* were maintained in a TYI-S-33 medium supplemented with 10% calf serum and bovine bile. *E. histolytica* and *T. vaginalis* trophozoites were maintained in TYI-S-33 medium supplemented with 10% bovine serum. Briefly, 5 × 10^4^ trophozoites of *G. intestinalis* or *T. vaginalis*, or 6 × 10^3^ trophozoites of *E. histolytica* were incubated for 48 h at 37 °C with different concentrations of the compound to be tested, each added as solutions in DMSO. As a negative control, parasite cultures received an equivalent amount of DMSO only, while albendazole and metronidazole were included as positive controls. At the end of the treatment period, the cells were washed and subcultured for another 48 h in a fresh medium to which no drug was added. The trophozoites were then counted with a haemocytometer and the 50% inhibitory concentration (IC_50_), together with the respective 95% confidence limit was calculated by Probit analysis. Experiments were carried out in triplicate and repeated at least twice.

#### 3.3.2. Antibacterial and Anticandidal Assays

*Escherichia coli* strain EDL933 (EHEC), *Escherichia coli* strain 042 (EAEC), *Salmonella enterica* serovar Typhi, *Candida albicans*, and *Candida glabrata* were used. The susceptibility assays were carried out using the disk diffusion test, in accordance with the outlined by CLSI (M02-A12) [18]. The inoculum was adjusted to 1.5 × 10^8^ CFU/mL (0.5 McFarland). Sensi-Discs (Becton Dickinson and Company, Sparks, MD, USA) were used as growth inhibition controls. Ciprofloxacin (10 µg) and ampicillin (25 µg) were used as antibiotics for the positive control; whereas ketoconazole (50 μg) was used as antimycotic. Each compound was tested at 5 mg per disc. Petri dishes with the compounds and the positive control were incubated at 37 °C for 24 h. The degree of effectiveness was measured by determining the zone of inhibition in millimeters resulting from the compounds. Experiments were carried out in triplicate and the results are reported as average values.

#### 3.3.3. Cytotoxicity Assays in Human Cells

HeLa (human cervical carcinoma) and HaCaT (immortalized human keratinocytes) cells were grown in DMEM (Invitrogen Corporation, Carlsbad, CA, USA) supplemented with 10% FBS (BioWest, Riverside, MO, USA), and maintained in standard culture conditions (37 °C, 95% humidity, and 5% CO_2_). Cells were allowed to grow to a density of 80% and then were harvested using sterile PBS/EDTA (pH 7.4) before starting every experiment. Cells were seeded in 96-well plates (7000 cells/well in 200 µL of DMEM). After 24 h the cells were exposed to test compounds dissolved in DMSO (J.T. Baker, Phillipsburg, NJ, USA) at different concentrations and diluted in 50 µL of DMEM, to reach 250 µL in the well. The exposure time was 48 h, and then viability was determined by MTT assay. The absorbance of formazan was determined for each well and its viability was related to the vehicle (100%). The IC_50_ was calculated from dose-response curve by non-linear fit using OriginPro 7.0 software (RockWare, Golden, CO, USA).

#### 3.3.4. Cyclooxygenase Assays

The in vitro assays on human recombinant cyclooxygenase-2 were performed using the COX inhibitor screening assay kit manufactured by Cayman Chemical, catalog number 560131 (Ann Arbor, MI, USA). Assays were carried out by duplicate following the instructions provided by the manufacturer. Compounds were tested at 10 µM, whereas the reference compound celecoxib (purchased from Sigma-Aldrich, catalog number PHR1683) was tested at 1 µM because of its high inhibition at 10 µM.

### 3.4. Molecular Docking

The crystal structure of human COX-2 was retrieved from the Protein Data Bank (www.rcsb.org; www.wwpdb.org) [46,47], PDB ID: 5KIR [33]. The protein structure was prepared using Maestro 9.1 (Schrödinger, Cambridge, MA, USA) [48]; first, chain B was selected and solvent molecules were removed. Then, the pdb structure was submitted for minimization using the YASARA web server (YASARA, Vienna, Austria) [49]. The protein was exported to Autodock Tools 1.5.6 (TSRI, La Jolla, CA, USA) and the grid coordinates and the pdbqt files were generated [50,51,52]. Ligands were constructed and minimized using the universal force field implemented in Maestro 9.1 [53] and exported to Autodock Tools 1.5.6 to generate the pdbqt files. Docking calculations were carried out using Autodock Vina (TSRI, La Jolla, CA, USA) employing a grid box of 40 × 40 × 40 centered on the co-crystalized ligand binding site (rofecoxib) and an exhaustiveness value of 500 [31]. The docking protocol was validated by comparison of docked rofecoxib and the co-crystalized rofecoxib. Molecular graphics and analyses were performed with the UCSF Chimera package version 1.10.2 (RBVI, San Francisco, CA, USA) [54].

## Figures and Tables

**Table 1:**
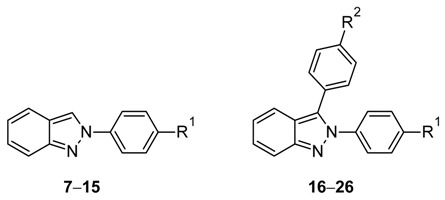
Antiprotozoal activity of 2-pheny-2*H*-indazole derivatives and 2,3-diphenyl-2*H*-indazole derivatives.

Compound	R^1^	R^2^	IC_50_ (µM) *G. intestinalis*	IC_50_ (µM) *E. histolytica*	IC_50_ (µM) *T. vaginalis*
**7**	H	–	0.1133 ± 0.0218	0.0798 ± 0.0036	0.1184 ± 0.0218
**8**	Cl	–	0.0634 ± 0.0031	0.0415 ± 0.0031	0.1071 ± 0.0031
**9**	OCH_3_	–	0.2051 ± 0.0063	0.1538 ± 0.0158	0.3723 ± 0.0158
**10**	COOCH_3_	–	0.0634 ± 0.0056	0.0218 ± 0.0028	0.1070 ± 0.0056
**11**	SCH_3_	–	0.2185 ± 0.0088	0.0978 ± 0.0147	0.2725 ± 0.0147
**12**	OH	–	0.1189 ± 0.0067	0.0737 ± 0.0101	0.1570 ± 0.0135
**13**	COOH	–	0.1931 ± 0.0119	0.0965 ± 0.0059	0.3274 ± 0.0178
**14**	SOCH_3_	–	0.1678 ± 0.0110	0.0878 ± 0.0083	0.3121 ± 0.0110
**15**	SO_2_CH_3_	–	0.0900 ± 0.0234	0.1359 ± 0.0052	0.1450 ± 0.0026
**16**	H	H	0.0518 ± 0.0052	0.3033 ± 0.0105	0.0573 ± 0.0026
**17**	Cl	H	0.0607 ± 0.0023	0.0213 ± 0.0023	0.1034 ± 0.0023
**18**	COOCH_3_	H	0.0959 ± 0.0022	0.0502 ± 0.0022	0.1020 ± 0.0151
**20**	COOH	H	0.0795 ± 0.0045	0.0445 ± 0.0045	0.1113 ± 0.0180
**21**	SO_2_CH_3_	H	0.1242 ± 0.0122	0.2081 ± 0.0061	0.2138 ± 0.0101
**22**	H	Cl	0.1132 ± 0.0070	0.0394 ± 0.0000	0.1181 ± 0.0046
**23**	H	COOCH_3_	0.1188 ± 0.0086	0.0731 ± 0.0086	0.1431 ± 0.0043
**25**	H	COOH	0.1209 ± 0.0090	0.0509 ± 0.0000	0.2402 ± 0.0067
**26**	H	SO_2_CH_3_	0.1062 ± 0.0081	0.0459 ± 0.0081	0.1837 ± 0.0162
MTZ	–	–	1.2260 ± 0.1250	0.3798 ± 0.1461	0.2360 ± 0.0160
ABZ	–	–	0.0370 ± 0.0030	56.5334 ± 18.8445	1.5905 ± 0.0113

**Table 2:** Antimycotic effect for selected compounds.

Compound	MIC (mM) *C. albicans*	MIC (mM) *C. glabrata*
**18**	3.807	15.227
**23**	3.807	15.227
Ketoconazole	0.045	0.079

**Table 3:** Results for the in silico and in vitro evaluations against human COX-2 for selected compounds.

Compound	Docking Score (Lowest Energy Conformation)	% of Inhibition of COX-2
**7** ^1^	−8.0	Inactive
**16**	−9.7	Inactive
**18**	−9.5	50.01 ± 9.49
**21**	−10.1	44.45 ± 2.65
**23**	−10.0	36.35 ± 1.7
**26**	−11.1	41.22 ± 5.93
Celecoxib ^2^	−11.7	64.92 ± 2.36

^1^ Compounds **7**, **16**, **18**, **21**, **23**, and **26** were tested at 10 µM. ^2^ Reference tested at 1 µM.

**Table 4:** Results for cytotoxicity assays in HaCaT and HeLa cell lines.

Compound	% Viability (10 µM) HaCaT Cells ^1^	% Viability (10 µM) HeLa Cells	IC_50_ (µM) HaCaT cells ^2^	IC_50_ (µM) HeLa Cells
**16**	95.01 ± 2.44	93.04 ± 4.57	93.65 ± 17.30	125.00 ± 29.60
**18**	96.25 ± 4.14	94.14 ± 3.31	-	-
**23**	97.83 ± 5.19	93.72 ± 7.48	-	-

^1^ Percent of viability for selected compounds at 10 μM. ^2^ Half maximal inhibitory concentration for a selected compound.

## Supplementary Materials

The following are available online. **Figures S1–S49**: ^1^H-NMR and ^13^C-NMR spectra of compounds **7**–**26**, and HRMS spectra of compounds **14**, **15**, **17**–**21**, **25**, and **26**. **Table S1.** Antibacterial and antimycotic effect for selected compounds.
