# Supplementary material for: Synthesis and Biological Evaluation of 2H-Indazole Derivatives: Towards Antimicrobial and Anti-Inflammatory Dual Agents
Source: Molecules. 2017 Oct 31;22(11):1864. doi: 10.3390/molecules22111864 (PMC6150295; doi:10.3390/molecules22111864)
Supplement: Supplementary file 1 [file molecules-22-01864-s001.pdf]

Supplementary data

Synthesis and Biological Evaluation of 2*H*-indazole Derivatives: Towards Antimicrobial and Anti-inflammatory Dual Agents

Jaime Pérez-Villanueva[1,*] Lilián Yépez-Mulia[2], Ignacio González-Sánchez[3], Olivia Soria-Arteche[1], Teresita del Rosario Sainz-Espuñes[1], Marco A. Cerbón[4], Juan Francisco Palacios-Espinosa[1], Karen Rodríguez-Villar[1], Ana Karina Rodríguez-Vicente[1], Miguel Cortés-Gines[1], Zeltzin Custodio-Galván[1], Dante B. Estrada-Castro[1]

[1]*Departamento de Sistemas Biológicos, División de Ciencias Biológicas y de la Salud, Universidad Autónoma Metropolitana-Xochimilco (UAM-X), Ciudad de México 04960, México*

[2]*Unidad de Investigación Médica en Enfermedades Infecciosas y Parasitarias, IMSS, Ciudad de México 06720, México*

[3]*Catedrático CONACYT comisionado a Departamento de Sistemas Biológicos, División de Ciencias Biológicas y de la Salud, Universidad Autónoma Metropolitana-Xochimilco (UAM-X), Ciudad de México 04960, México*

[4]*Facultad de Química, Departamento de Biología, Universidad Nacional Autónoma de México, Ciudad de México, México.*

*Correspondence. jpvillanueva@correo.xoc.uam.mx (J.P.-V.); Tel.: +525 54 83 72 59; fax: +525 55 94 79 29.

Table of Contents:

	Page
**Figure S1**. ^{1}H NMR (600 MHz, $CDCl_3$) for 2-phenyl-2*H*-indazole (**7**)	S5
**Figure S2**. ^{13}C NMR (151 MHz, $CDCl_3$) for 2-phenyl-2*H*-indazole (**7**)	S6
**Figure S3**. ^{1}H NMR (600 MHz, $CDCl_3$) for 2-(4-chlorophenyl)-2*H*-indazole (**8**)	S7
**Figure S4**. ^{13}C NMR (151 MHz, $CDCl_3$) for 2-(4-chlorophenyl)-2*H*-indazole (**8**)	S8
**Figure S5**. ^{1}H NMR (600 MHz, $CDCl_3$) for 2-(4-methoxyphenyl)-2*H*-indazole (**9**)	S9
**Figure S6**. ^{13}C NMR (151 MHz, $CDCl_3$) for 2-(4-methoxyphenyl)-2*H*-indazole (**9**)	S10
**Figure S7**. ^{1}H NMR (600 MHz, $CDCl_3$) for methyl 4-(2*H*-indazol-2-yl)benzoate (**10**)	S11
**Figure S8**. ^{13}C NMR (151 MHz, $CDCl_3$) for methyl 4-(2*H*-indazol-2-yl)benzoate (**10**)	S12
**Figure S9**. ^{1}H NMR (600 MHz, $CDCl_3$) for 2-(4-(methylthio)phenyl)-2*H*-indazole (**11**)	S13
**Figure S1**. ^{13}C NMR (151 MHz, $CDCl_3$) for 2-(4-(methylthio)phenyl)-2*H*-indazole (**11**)	S14
**Figure S11**. ^{1}H NMR (600 MHz, DMSO-d_6) for 4-(2*H*-indazol-2-yl)phenol (**12**)	S15
**Figure S12**. ^{13}C NMR (151 MHz, DMSO-d_6) for 4-(2*H*-indazol-2-yl)phenol (**12**)	S16
**Figure S13**. ^{1}H NMR (600 MHz, DMSO-d_6) for 4-(2*H*-indazol-2-yl)benzoic acid (**13**)	S17
**Figure S14**. ^{13}C NMR (151 MHz, DMSO-d_6) for 4-(2*H*-indazol-2-yl)benzoic acid (**13**)	S18
**Figure S15**. ^{1}H NMR (600 MHz, $CDCl_3$) for 2-(4-(methylsulfinyl)phenyl)-2*H*-indazole (**14**)	S19
**Figure S16**. ^{13}C NMR (151 MHz, $CDCl_3$) for 2-(4-(methylsulfinyl)phenyl)-2*H*-indazole (**14**)	S20
**Figure S17**. MS (HR-ESI) for 2-(4-(methylsulfinyl)phenyl)-2*H*-indazole (**14**)	S21
**Figure S18**. ^{1}H NMR (600 MHz, $CDCl_3$) for 2-(4-(methylsulfonyl)phenyl)-2*H*-indazole (**15**)	S22

Figure S19. ^{13}C NMR (151 MHz, $CDCl_3$) for 2-(4-(methylsulfonyl)phenyl)-2*H*-indazole (**15**) S23

Figure S20. MS (HR-ESI) for 2-(4-(methylsulfonyl)phenyl)-2*H*-indazole (**15**) S24

Figure S21. ^{1}H NMR (600 MHz, $CDCl_3$) for 2,3-diphenyl-2*H*-indazole (**16**) S25

Figure S22. ^{13}C NMR (151 MHz, $CDCl_3$) for 2,3-diphenyl-2*H*-indazole (**16**) S26

Figure S23. ^{1}H NMR (600 MHz, $CDCl_3$) for 2-(4-chlorophenyl)-3-phenyl-2*H*-indazole (**17**) S27

Figure S24. ^{13}C NMR (151 MHz, $CDCl_3$) for 2-(4-chlorophenyl)-3-phenyl-2*H*-indazole (**17**) S28

Figure S25. MS (HR-ESI) for 2-(4-chlorophenyl)-3-phenyl-2*H*-indazole (**17**) S29

Figure S26. ^{1}H NMR (600 MHz, $CDCl_3$) for methyl 4-(3-phenyl-2*H*-indazol-2-yl)benzoate (**18**) S30

Figure S27. ^{13}C NMR (151 MHz, $CDCl_3$) for methyl 4-(3-phenyl-2*H*-indazol-2-yl)benzoate (**18**) S31

Figure S28. MS (HR-ESI) for methyl 4-(3-phenyl-2*H*-indazol-2-yl)benzoate (**18**) S32

Figure S29. ^{1}H NMR (600 MHz, $CDCl_3$) for 2-(4-(methylthio)phenyl)-3-phenyl-2*H*-indazole (**19**) S33

Figure S30. ^{13}C NMR (151 MHz, $CDCl_3$) for 2-(4-(methylthio)phenyl)-3-phenyl-2*H*-indazole (**19**) S34

Figure S31. MS (HR-ESI) for 2-(4-(methylthio)phenyl)-3-phenyl-2*H*-indazole (**19**) S35

Figure S32. ^{1}H NMR (600 MHz, DMSO-d_6) for 4-(3-phenyl-2*H*-indazol-2-yl)benzoic acid (**20**) S36

Figure S33. ^{13}C NMR (151 MHz, DMSO-d_6) for 4-(3-phenyl-2*H*-indazol-2-yl)benzoic acid (**20**) S37

Figure S34. MS (HR-ESI) for 4-(3-phenyl-2*H*-indazol-2-yl)benzoic acid (**20**) S38

Figure S35. ^{1}H NMR (600 MHz, $CDCl_3$) for 2-(4-(methylsulfonyl)phenyl)-3-phenyl-2*H*-indazole (**21**) S39

Figure S36. ^{13}C NMR (151 MHz, $CDCl_3$) for 2-(4-(methylsulfonyl)phenyl)-3-phenyl-2*H*-indazole (**21**) S40

Figure S37. MS (HR-ESI) for 2-(4-(methylsulfonyl)phenyl)-3-phenyl-2*H*-indazole (**21**) S41

Figure S38. ^{1}H NMR (600 MHz, $CDCl_3$) for 3-(4-chlorophenyl)-2-phenyl-2*H*-indazole (**22**) S42

Figure S39. ^{13}C NMR (151 MHz, $CDCl_3$) for 3-(4-chlorophenyl)-2-phenyl-2*H*-indazole (**22**) S43

Figure S40. ^{1}H NMR (600 MHz, $CDCl_3$) for methyl 4-(2-phenyl-2*H*-indazol-3-yl)benzoate (**23**) S44

Figure S41. ^{13}C NMR (151 MHz, $CDCl_3$) for methyl 4-(2-phenyl-2*H*-indazol-3-yl)benzoate (**23**) S45

Figure S42. ^{1}H NMR (600 MHz, $CDCl_3$) for 3-(4-(methylthio)phenyl)-2-phenyl-2*H*-indazole (**24**) S46

Figure S43. ^{13}C NMR (151 MHz, $CDCl_3$) for 3-(4-(methylthio)phenyl)-2-phenyl-2*H*-indazole (**24**) S47

Figure S44. ^{1}H NMR (600 MHz, DMSO-d_6) for 4-(2-phenyl-2*H*-indazol-3-yl)benzoic acid (**25**) S48

Figure S45. ^{13}C NMR (151 MHz, DMSO-d_6) for 4-(2-phenyl-2*H*-indazol-3-yl)benzoic acid (**25**) S49

Figure S46. MS (HR-ESI) for 4-(2-phenyl-2*H*-indazol-3-yl)benzoic acid (**25**) S50

Figure S47. ^{1}H NMR (600 MHz, $CDCl_3$) for 3-(4-(methylsulfonyl)phenyl)-2-phenyl-2*H*-indazole (**26**) S51

Figure S48. ^{13}C NMR (151 MHz, $CDCl_3$) for 3-(4-(methylsulfonyl)phenyl)-2-phenyl-2*H*-indazole (**26**) S52

Figure S49. MS (HR-ESI) for 3-(4-(methylsulfonyl)phenyl)-2-phenyl-2*H*-indazole (**26**) S53

Table S1. Antibacterial and antimycotic effect for selected compounds S54

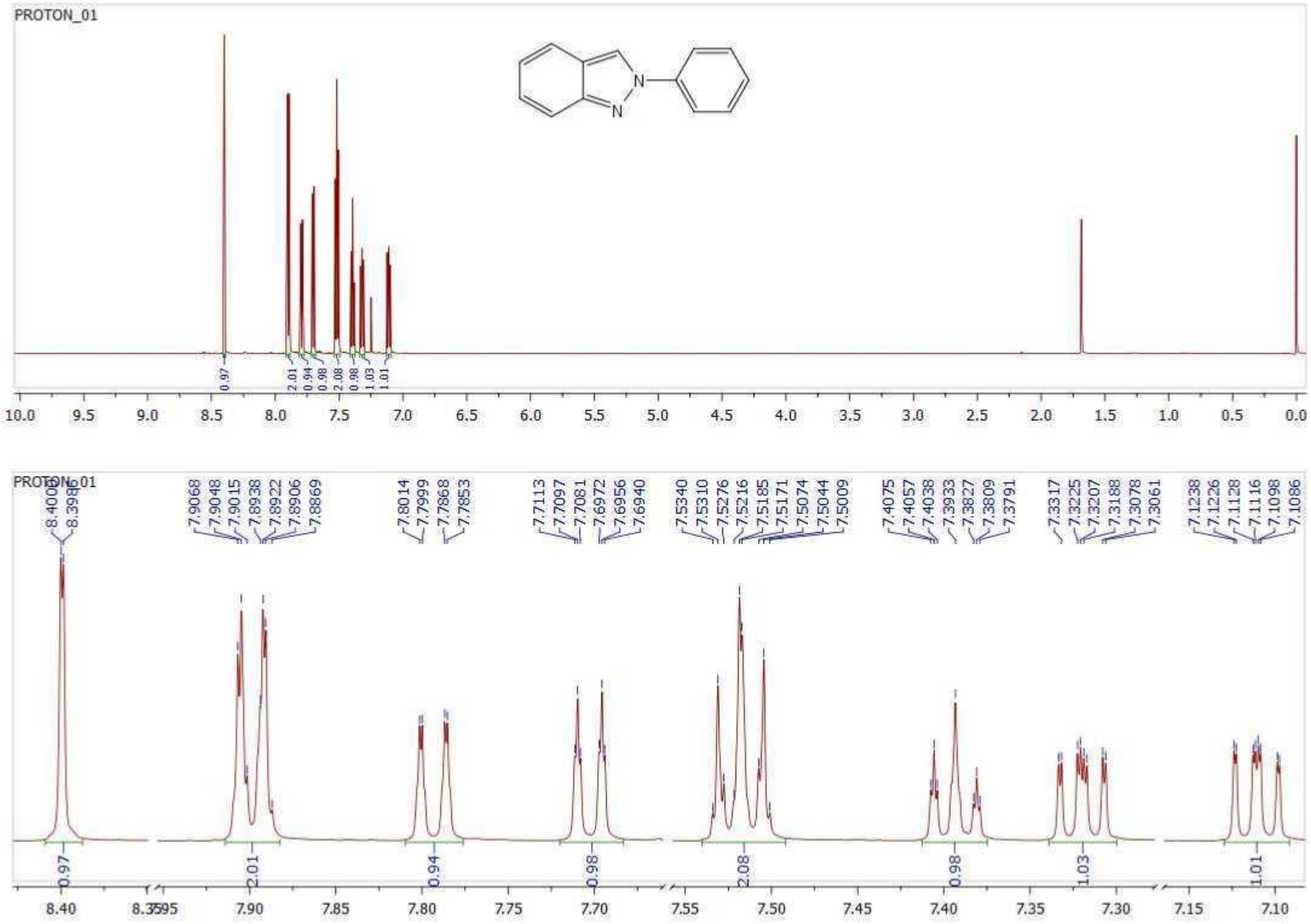

Figure S1. ^{1}H NMR (600 MHz, $CDCl_3$) for 2-phenyl-2*H*-indazole (**7**)

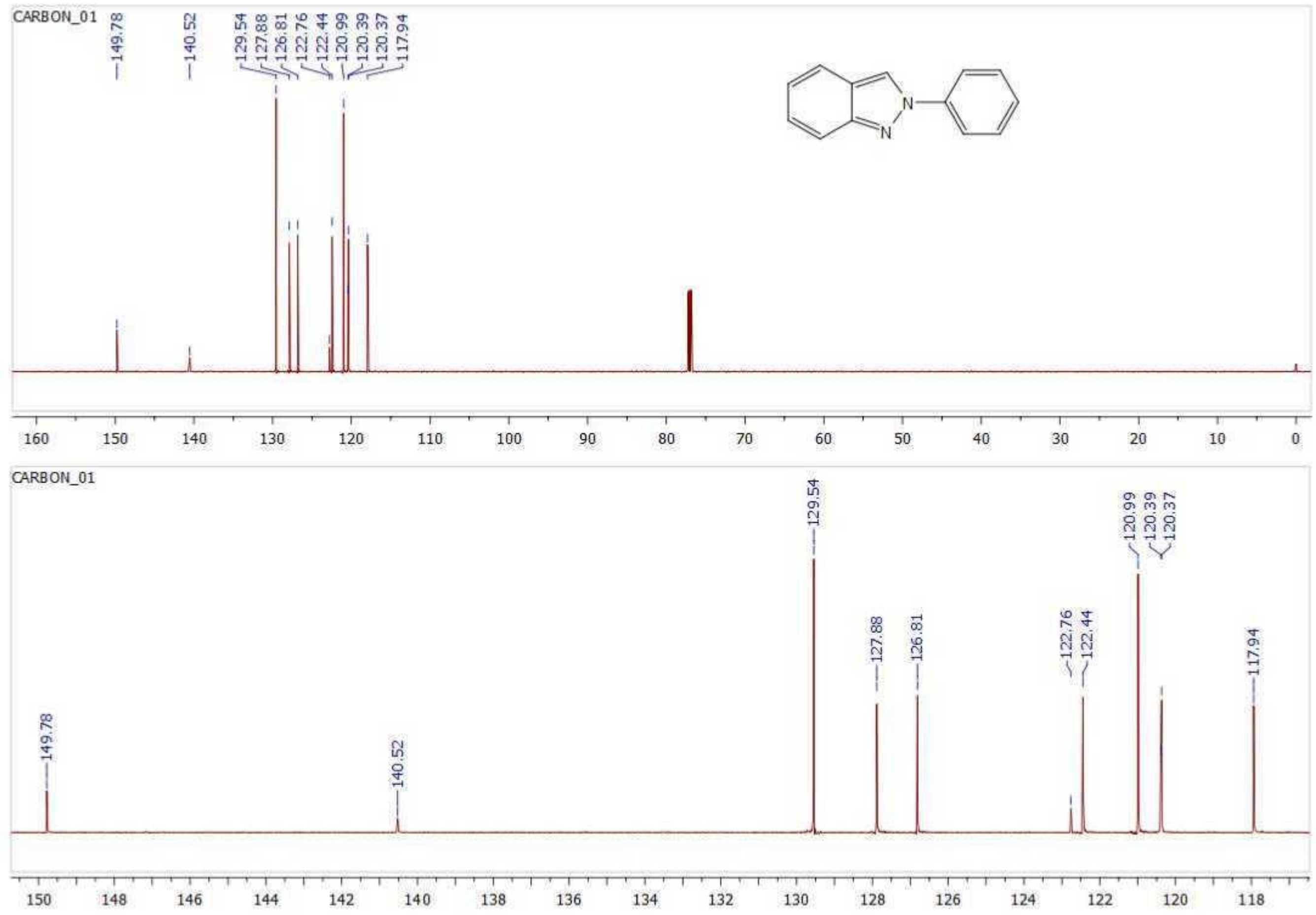

Figure S2. ^{13}C NMR (151 MHz, $CDCl_3$) for 2-phenyl-2*H*-indazole (**7**)

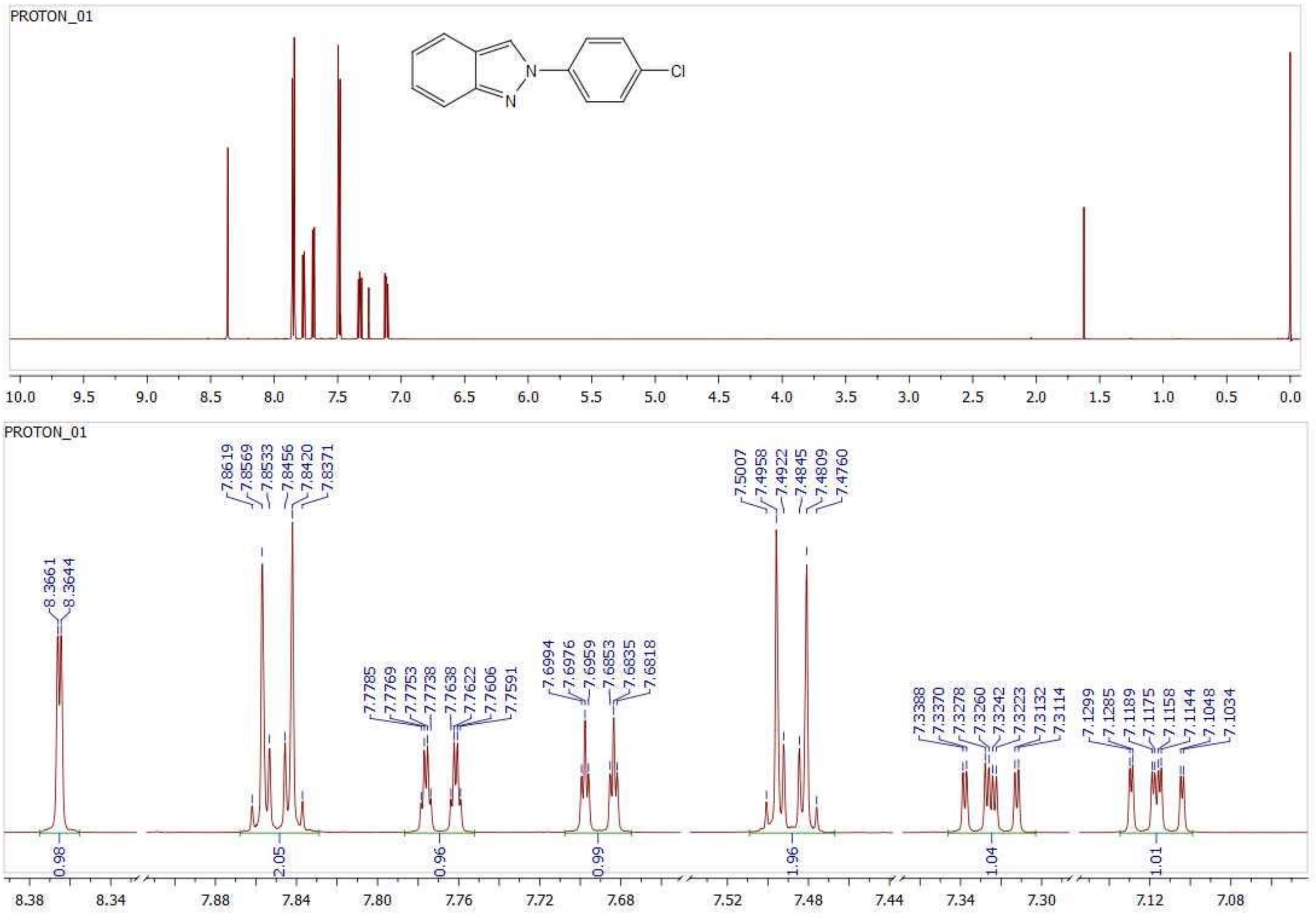

Figure S3. ¹H NMR (600 MHz, $CDCl_3$) for 2-(4-chlorophenyl)-2*H*-indazole (**8**)

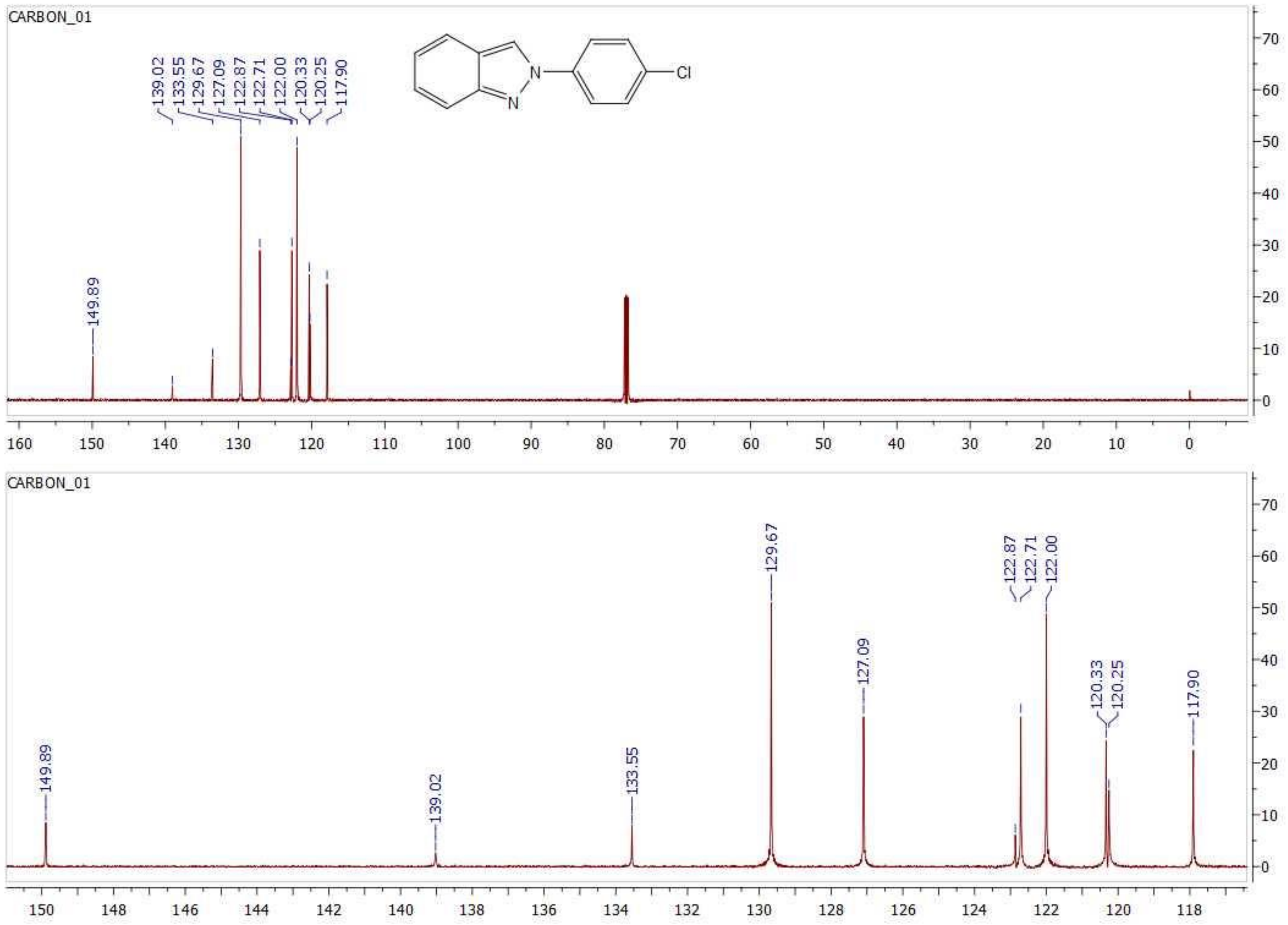

Figure S4. ^{13}C NMR (151 MHz, $CDCl_3$) for 2-(4-chlorophenyl)-2*H*-indazole (**8**)

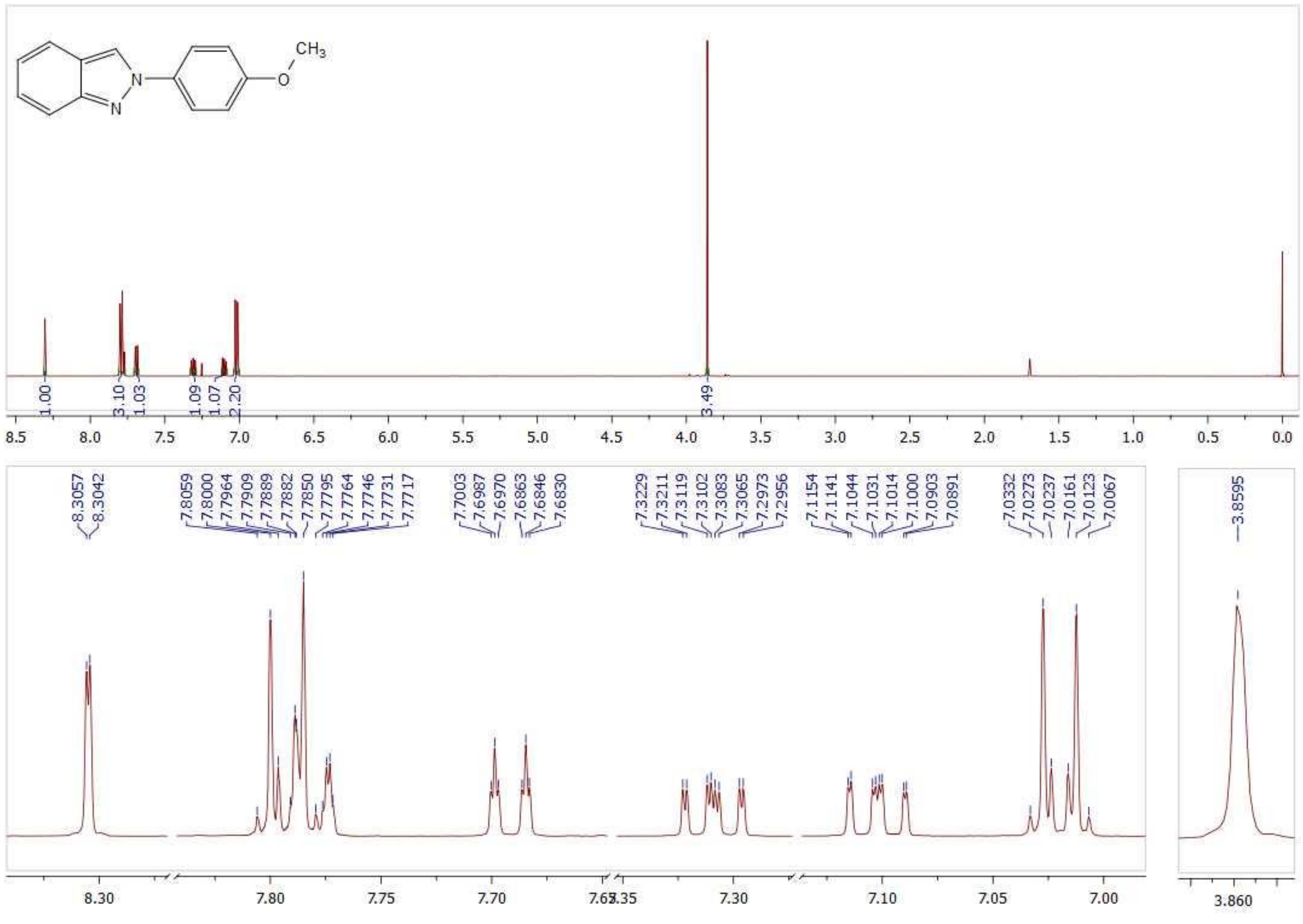

Figure S5. ^{1}H NMR (600 MHz, $CDCl_3$) for 2-(4-methoxyphenyl)-2*H*-indazole (**9**)

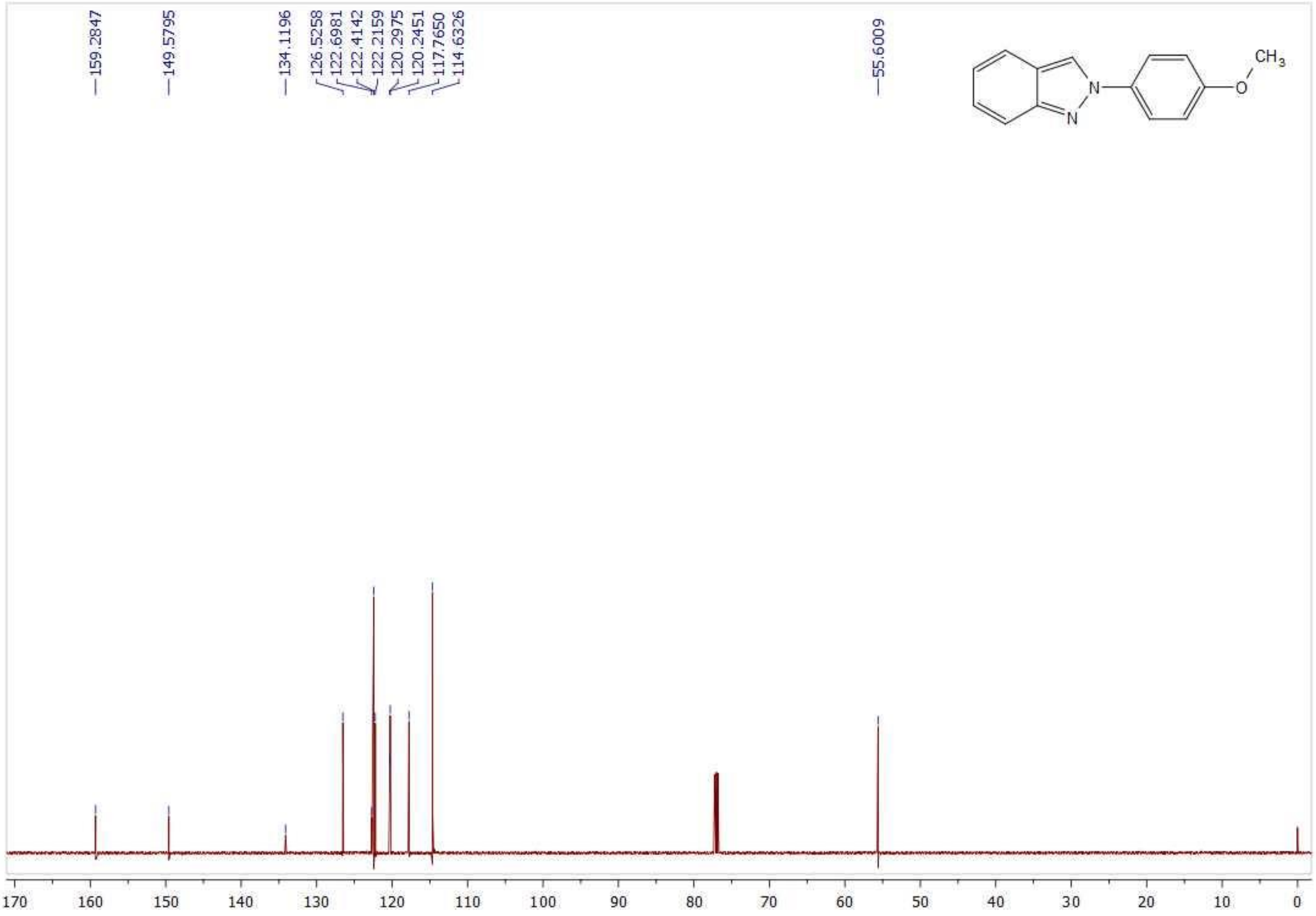

Figure S6. ^{13}C NMR (151 MHz, $CDCl_3$) for 2-(4-methoxyphenyl)-2*H*-indazole (**9**)

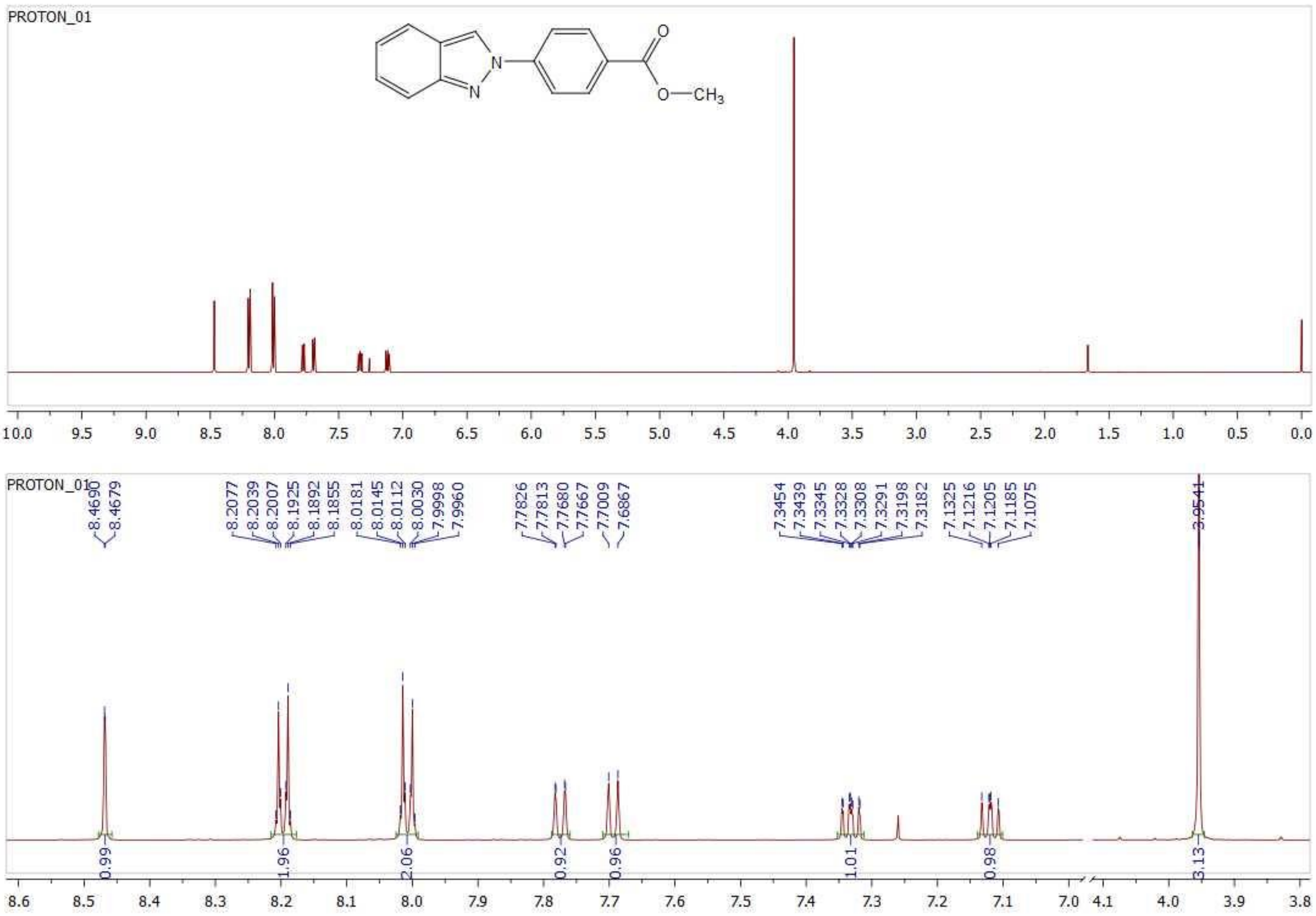

Figure S7. ^{1}H NMR (600 MHz, $CDCl_3$) for methyl 4-(2*H*-indazol-2-yl)benzoate (**10**)

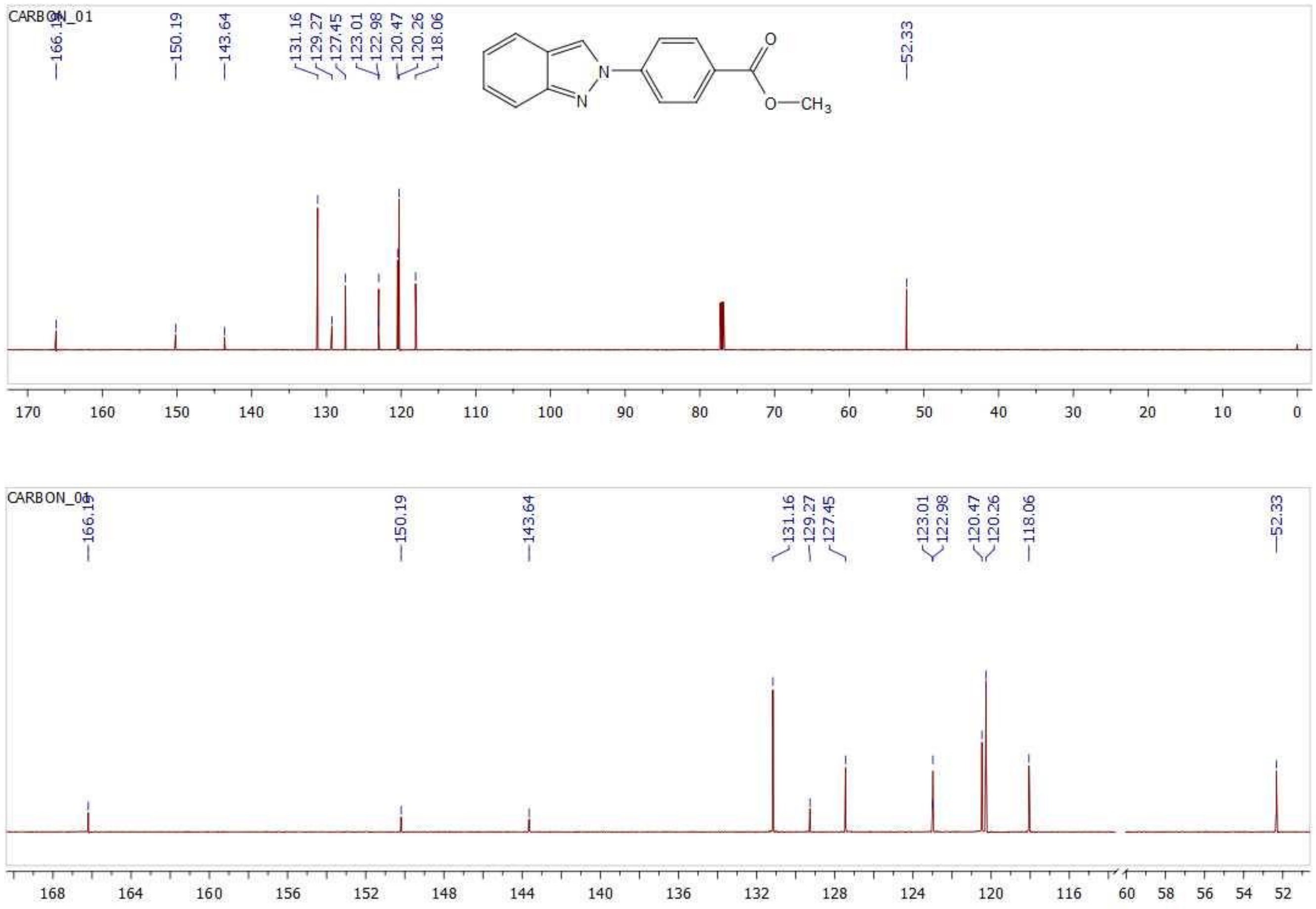

Figure S8. ^{13}C NMR (151 MHz, $CDCl_3$) for methyl 4-(2*H*-indazol-2-yl)benzoate (**10**)

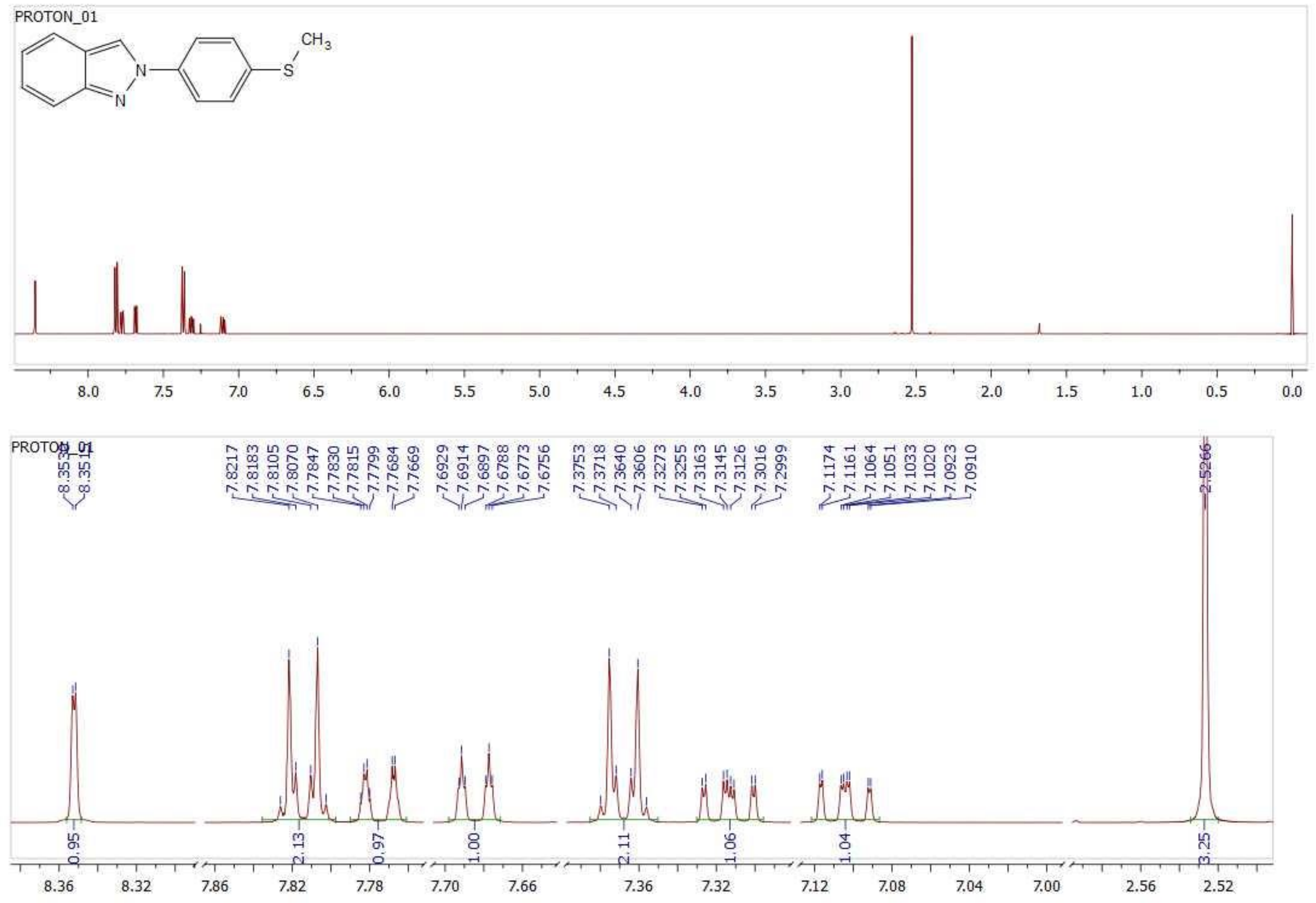

Figure S9. ^{1}H NMR (600 MHz, $CDCl_3$) for 2-(4-(methylthio)phenyl)-2*H*-indazole (**11**)

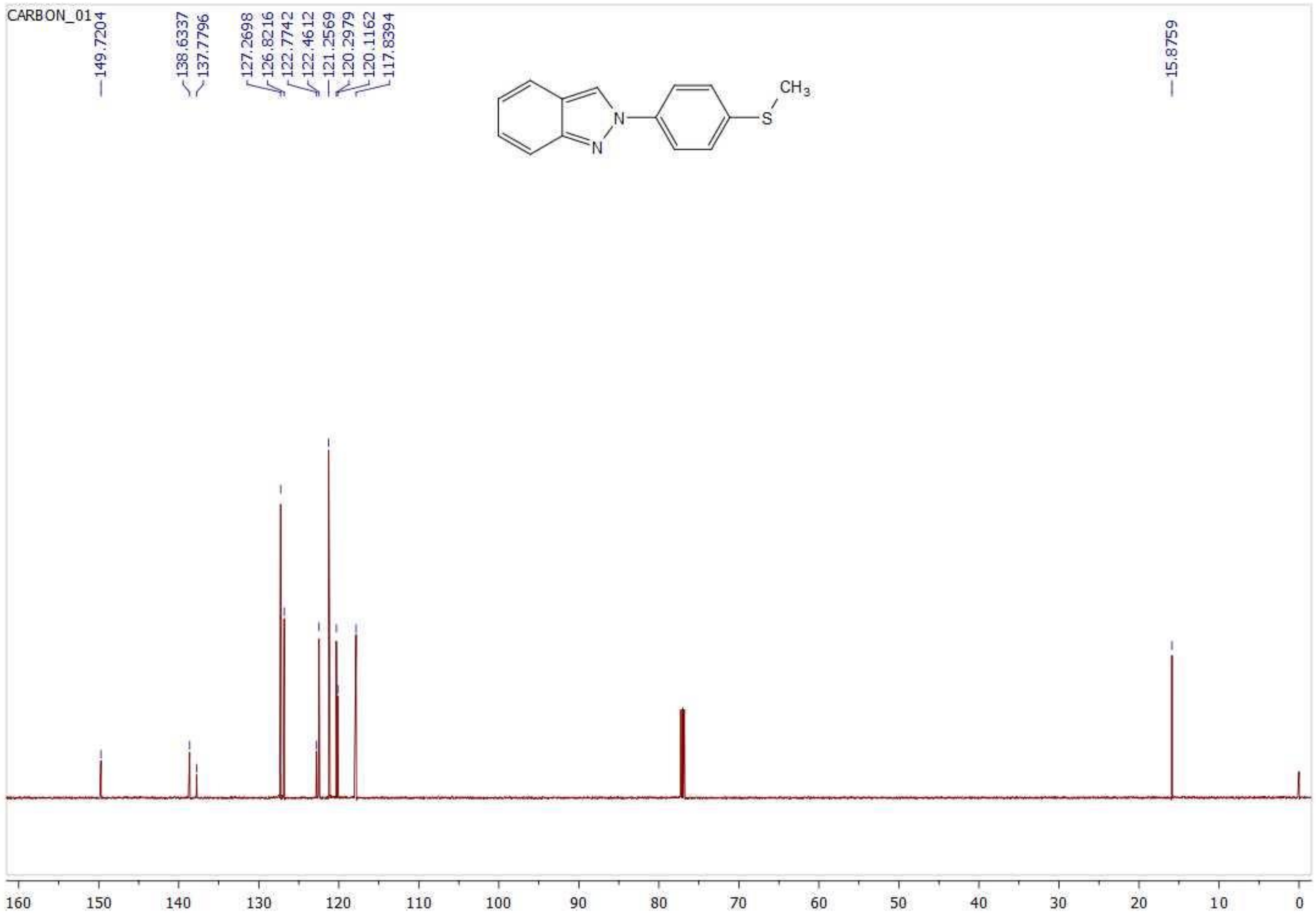

Figure S10. ^{13}C NMR (151 MHz, $CDCl_3$) for 2-(4-(methylthio)phenyl)-2*H*-indazole (**11**)

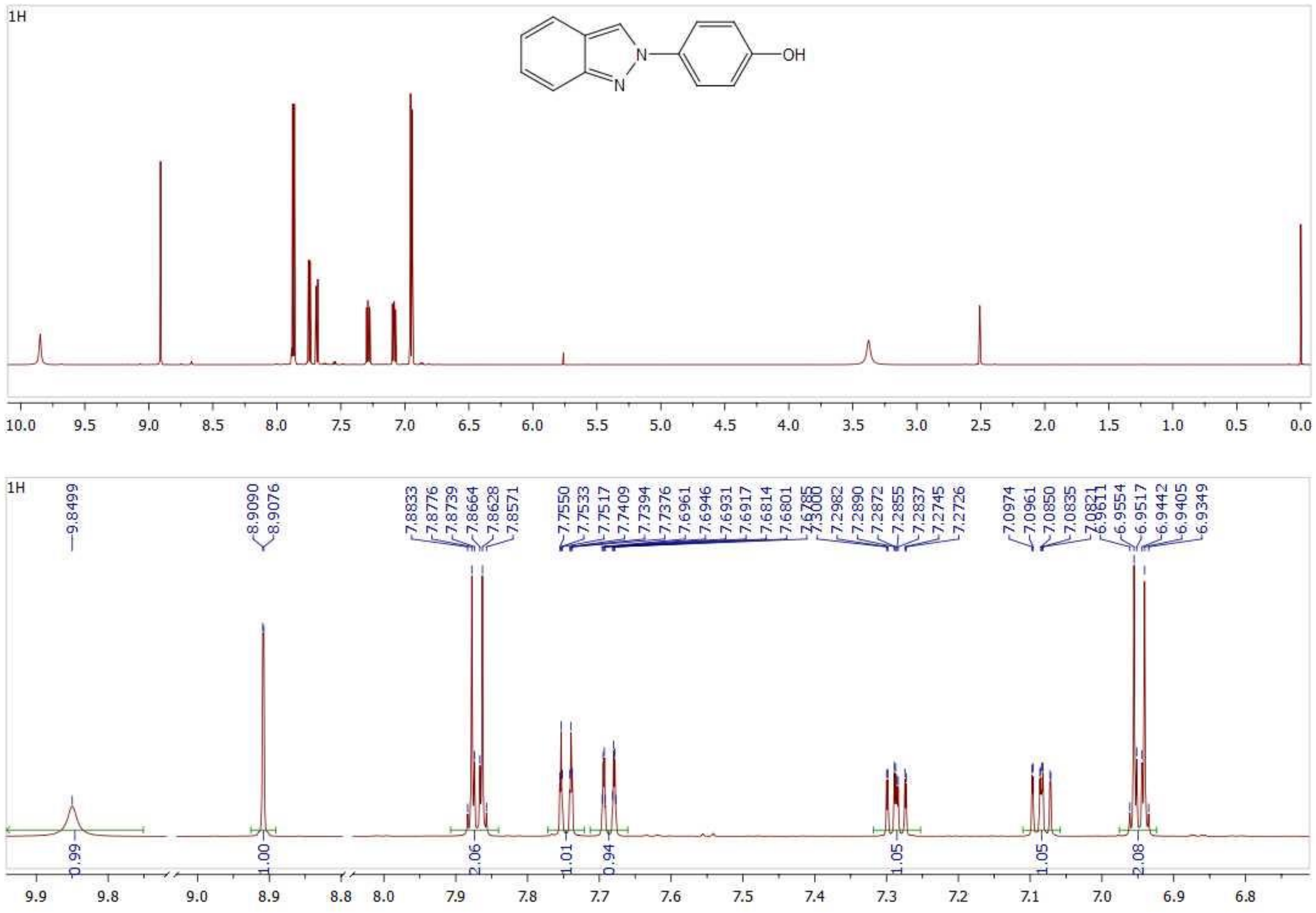

Figure S11. ^{1}H NMR (600 MHz, DMSO-d_6) for 4-(2*H*-indazol-2-yl)phenol (**12**)

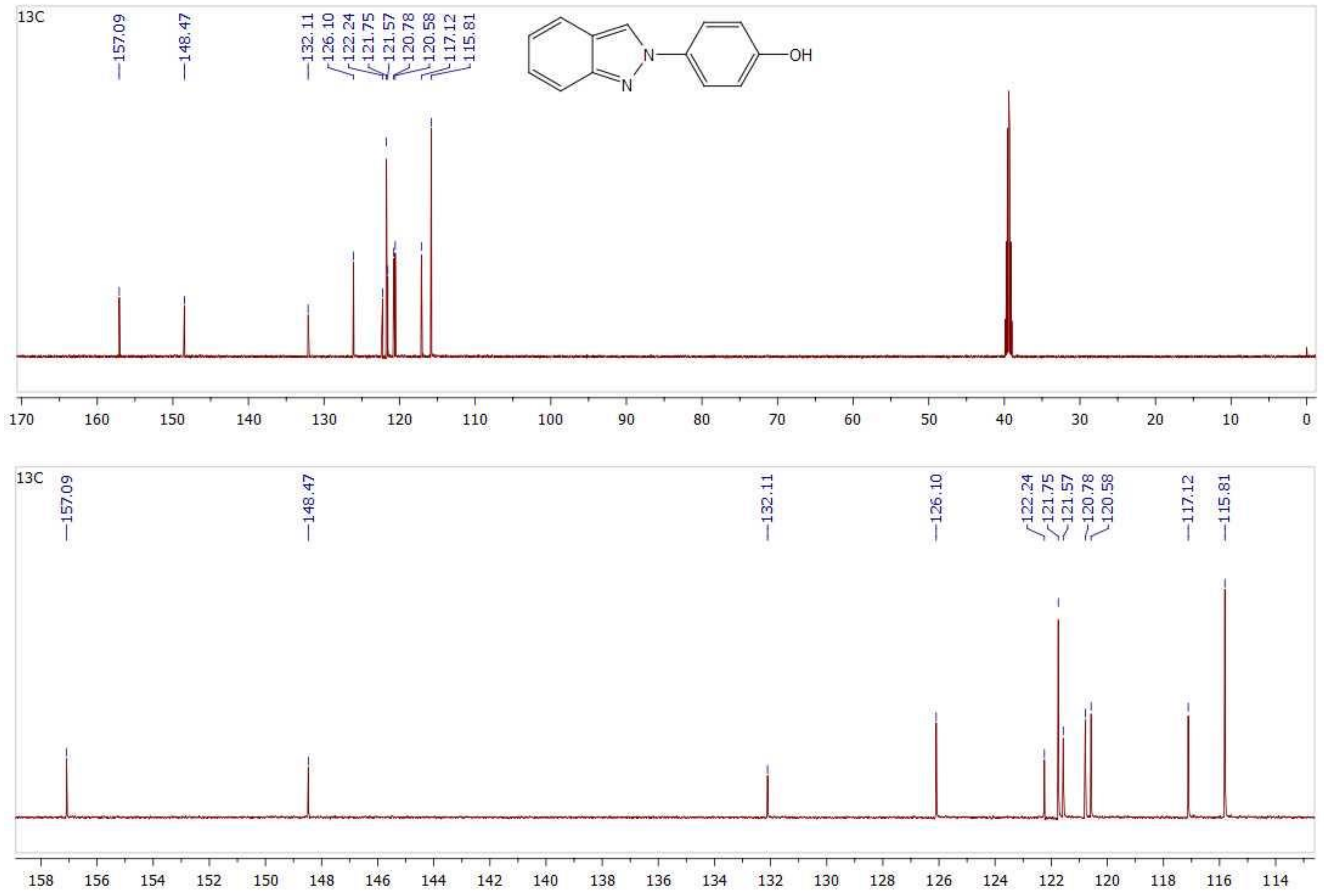

Figure S12. ^{13}C NMR (151 MHz, DMSO-d_6) for 4-(2*H*-indazol-2-yl)phenol (**12**)

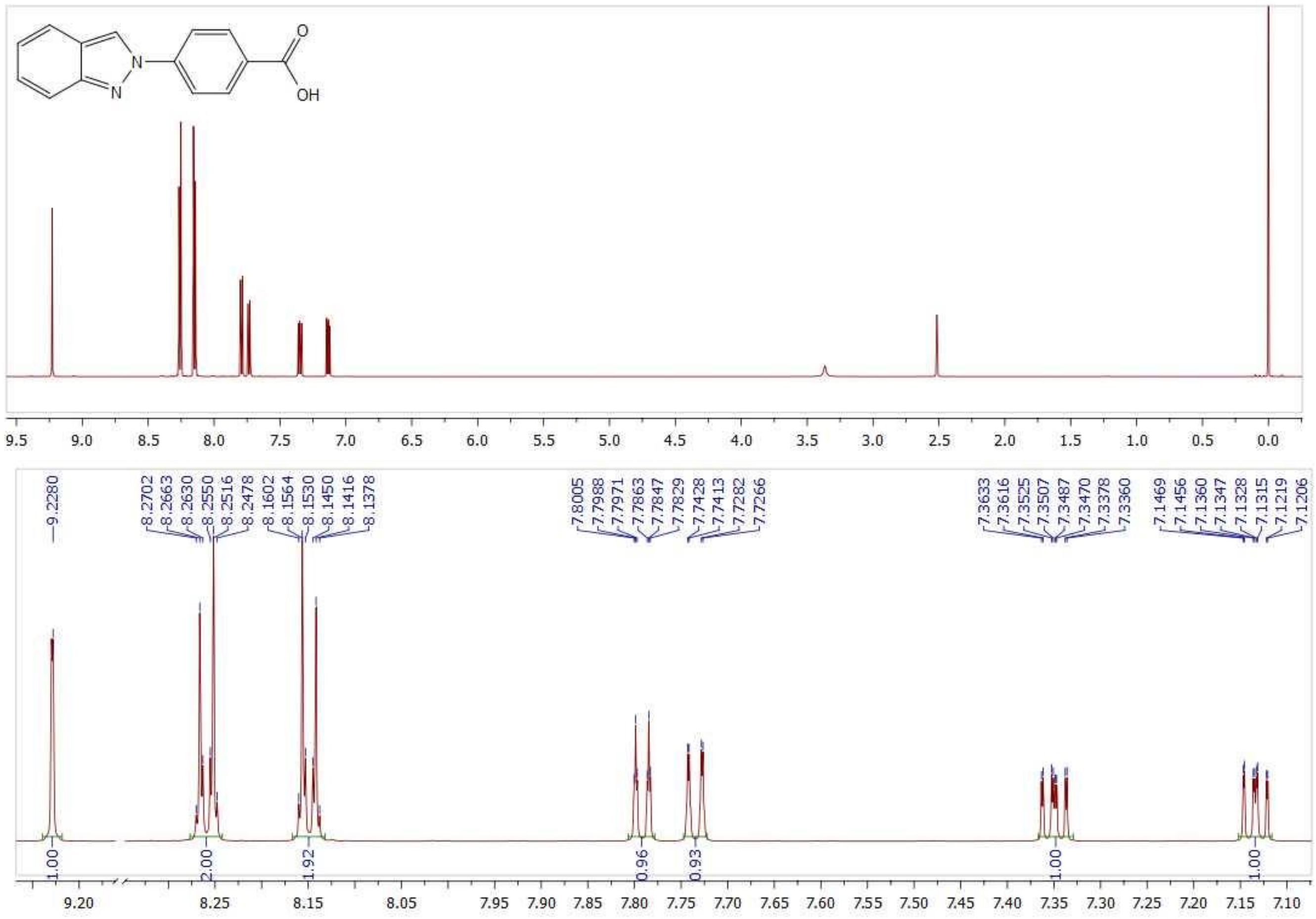

Figure S13. ^{1}H NMR (600 MHz, DMSO-d_6) for 4-(2*H*-indazol-2-yl)benzoic acid (**13**)

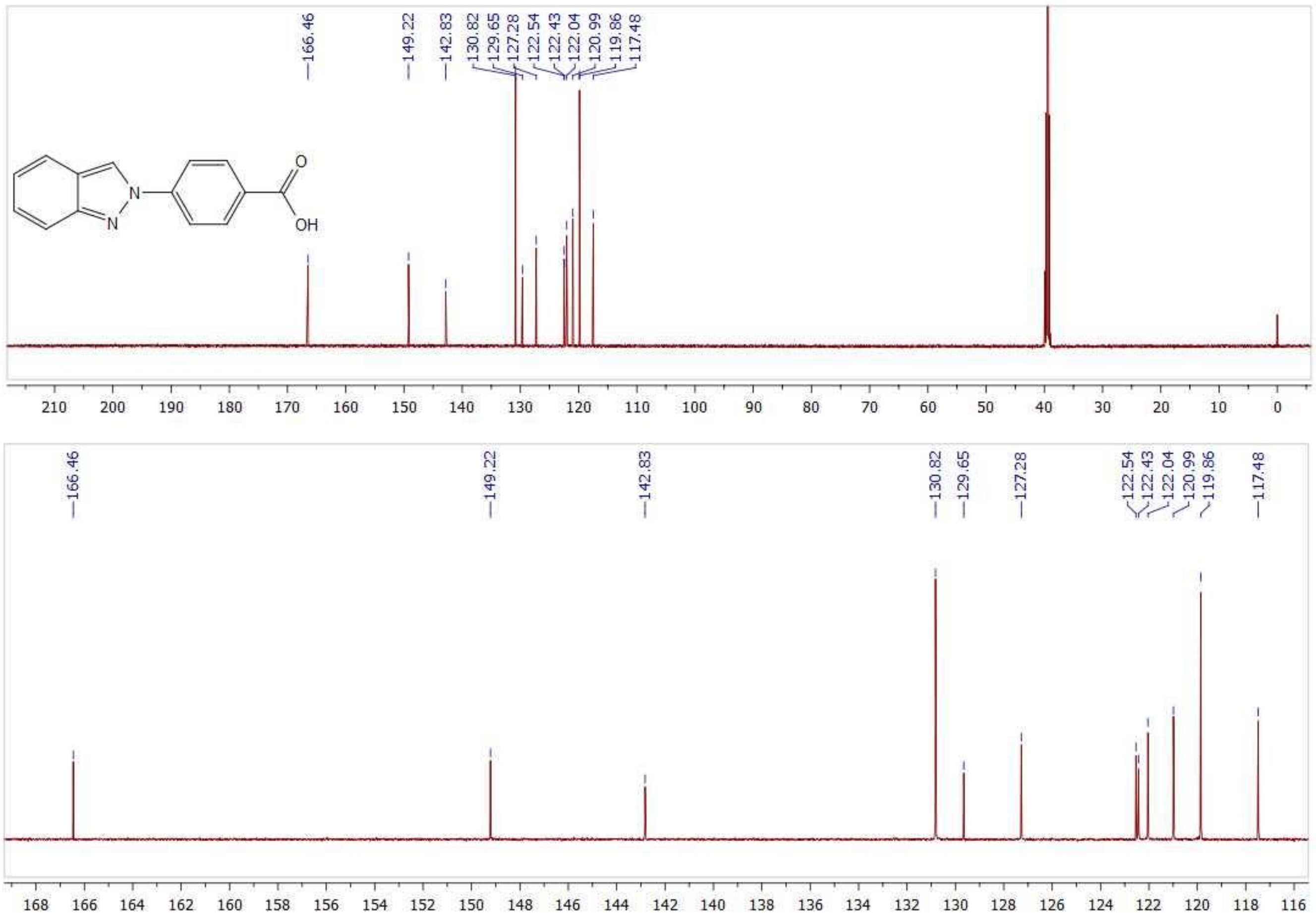

Figure S14. ${}^{13}C$ NMR (151 MHz, DMSO-d_6) for 4-(2*H*-indazol-2-yl)benzoic acid (**13**)

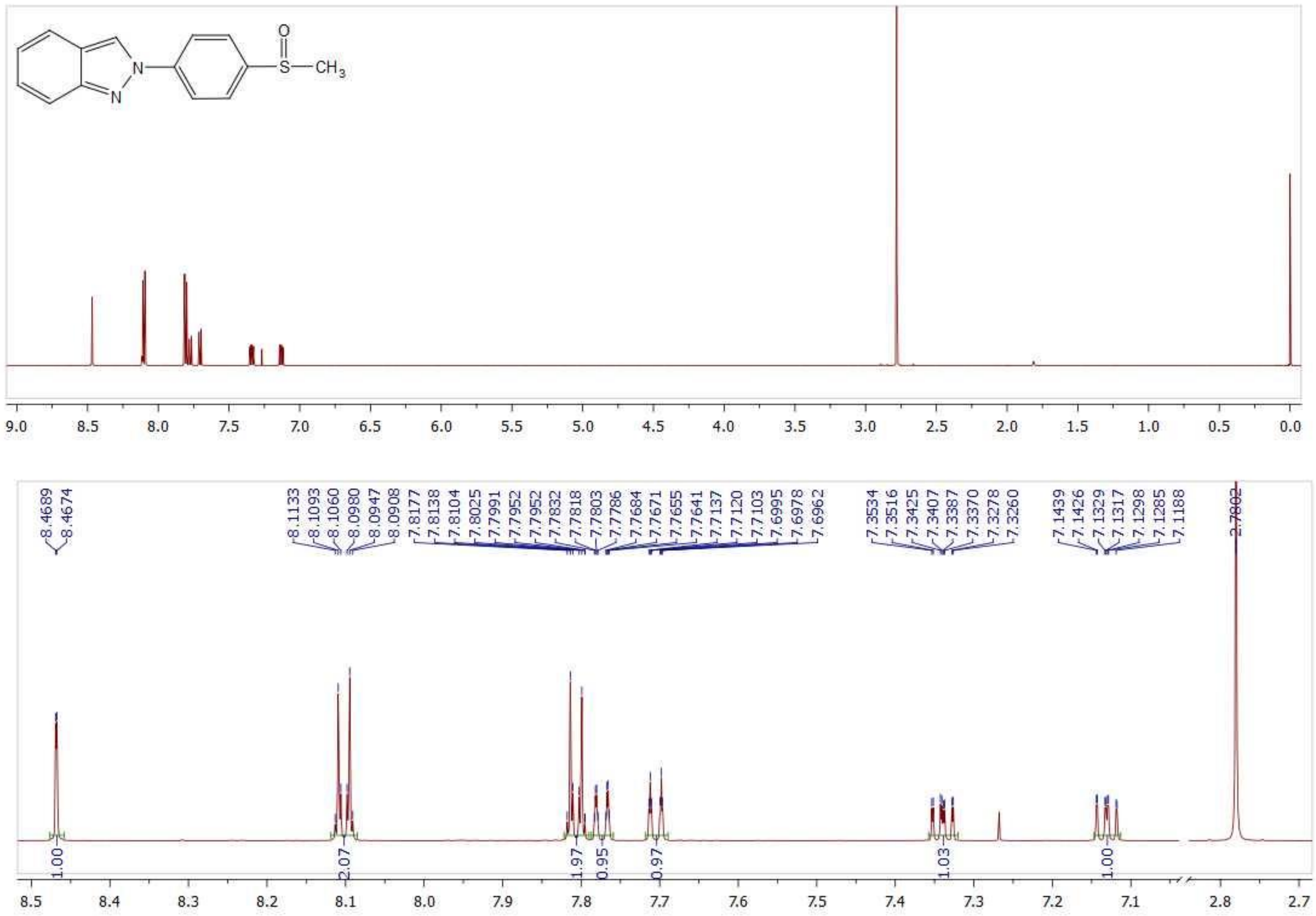

Figure S15. ^{1}H NMR (600 MHz, $CDCl_3$) for 2-(4-(methylsulfinyl)phenyl)-2*H*-indazole (**14**)

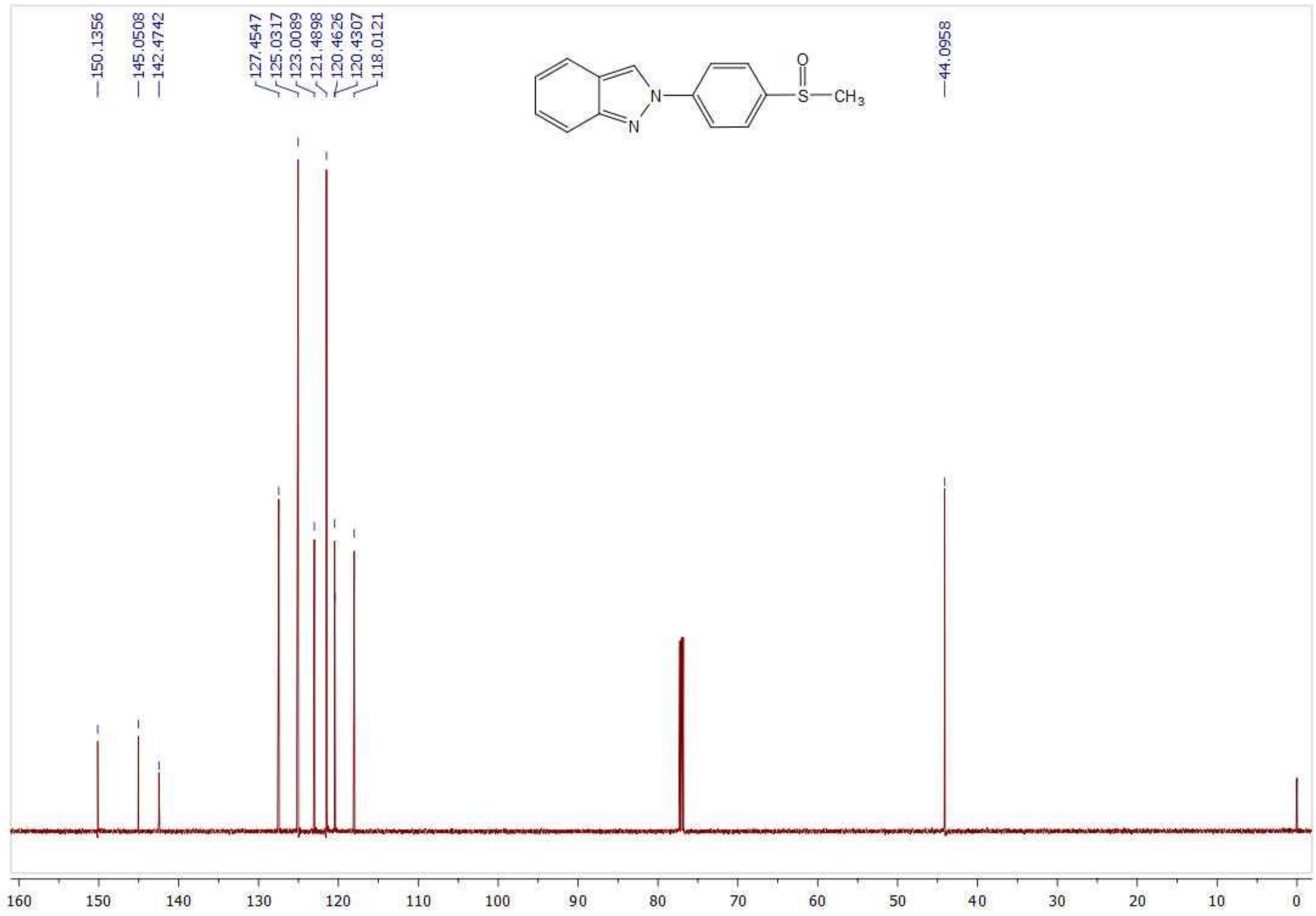

Figure S16. ^{13}C NMR (151 MHz, $CDCl_3$) for 2-(4-(methylsulfinyl)phenyl)-2*H*-indazole (**14**)

Acquisition Parameter

Source Type	ESI	Ion Polarity	Positive	Set Nebulizer	0.3 Bar
Focus	Active	Set Capillary	4500 V	Set Dry Heater	180 °C
Scan Begin	50 m/z	Set End Plate Offset	-500 V	Set Dry Gas	4.0 l/min
Scan End	3000 m/z	Set Charging Voltage	0 V	Set Divert Valve	Waste
		Set Corona	0 nA	Set APCI Heater	0 °C

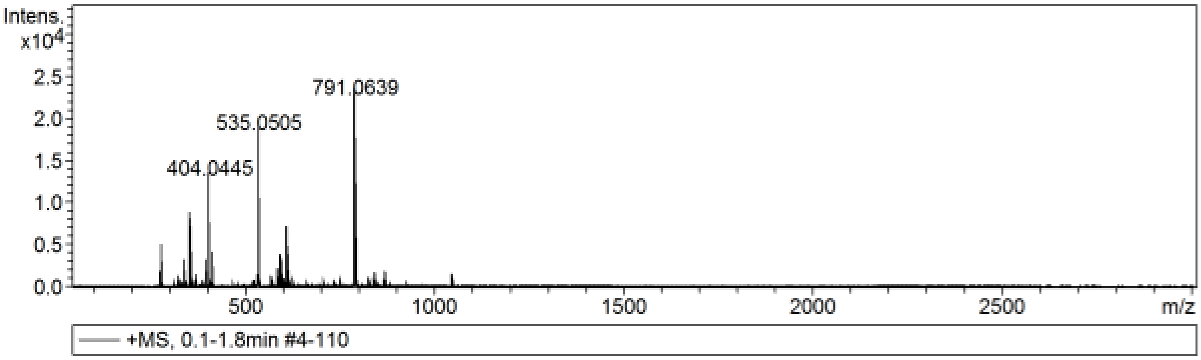

#	m/z	Res.	S/N	I	I %	FWHM
1	276.0445	4050	74.7	2009	9.1	0.0682
2	279.0481	3963	162.9	4386	19.8	0.0704
3	339.0389	3525	93.3	2929	13.2	0.0962
4	340.0229	2300	50.9	1600	7.2	0.1478
5	355.0136	3886	247.4	8052	36.4	0.0914
6	356.0074	3689	64.4	2102	9.5	0.0965
7	357.9896	3947	50.1	1638	7.4	0.0907
8	396.0578	3711	91.0	3265	14.7	0.1067
9	396.5526	3619	75.2	2698	12.2	0.1096
10	397.0529	3625	55.4	1990	9.0	0.1095
11	404.0445	3647	348.7	12589	56.8	0.1108
12	404.5450	3648	190.2	6864	31.0	0.1109
13	405.0441	3391	104.3	3765	17.0	0.1194
14	413.2203	3612	101.2	3721	16.8	0.1144
15	535.0505	3667	406.9	17911	80.9	0.1459
16	536.0543	3420	136.6	6023	27.2	0.1567
17	537.0484	3596	70.1	3089	13.9	0.1494
18	586.9828	3283	46.8	2128	9.6	0.1788
19	595.0473	3023	80.2	3638	16.4	0.1968
20	596.0485	3337	39.4	1786	8.1	0.1786
21	611.0202	3416	144.6	6543	29.5	0.1789
22	612.0222	3445	55.9	2528	11.4	0.1777
23	613.9968	3424	80.8	3646	16.5	0.1793
24	791.0639	3478	529.5	22145	100.0	0.2274
25	792.0671	5278	288.6	12060	54.5	0.1501
26	793.0653	3551	155.1	6478	29.3	0.2234
27	794.0663	3310	53.6	2236	10.1	0.2399
28	842.9986	3486	47.0	1831	8.3	0.2418
29	870.0083	5277	48.8	1843	8.3	0.1649
30	1047.0866	3349	50.4	1588	7.2	0.3127

Figure S17. MS (HR-ESI) for 2-(4-(methylsulfinyl)phenyl)-2*H*-indazole (**14**)

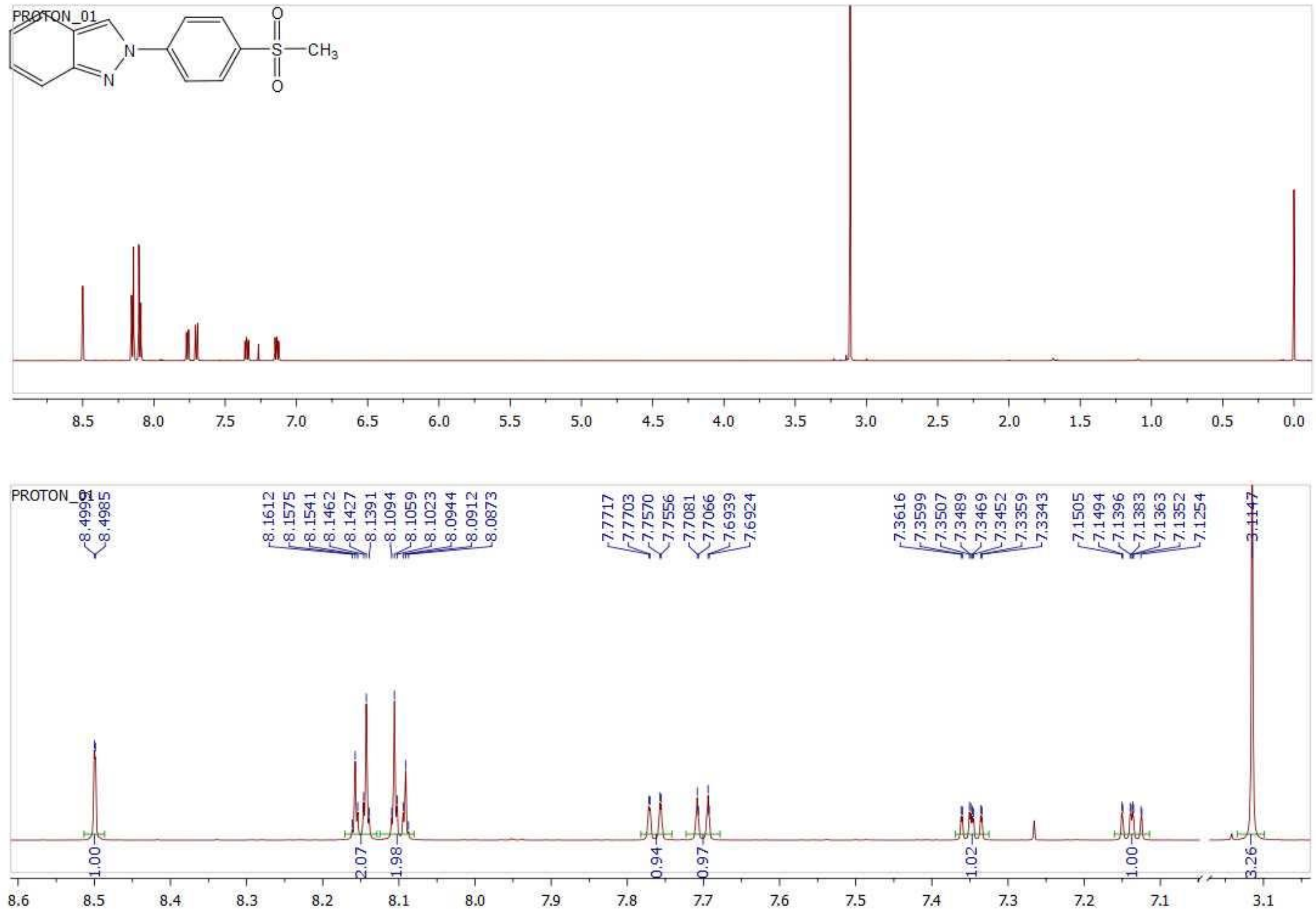

Figure S18. ^{1}H NMR (600 MHz, $CDCl_3$) for 2-(4-(methylsulfonyl)phenyl)-2*H*-indazole (**15**)

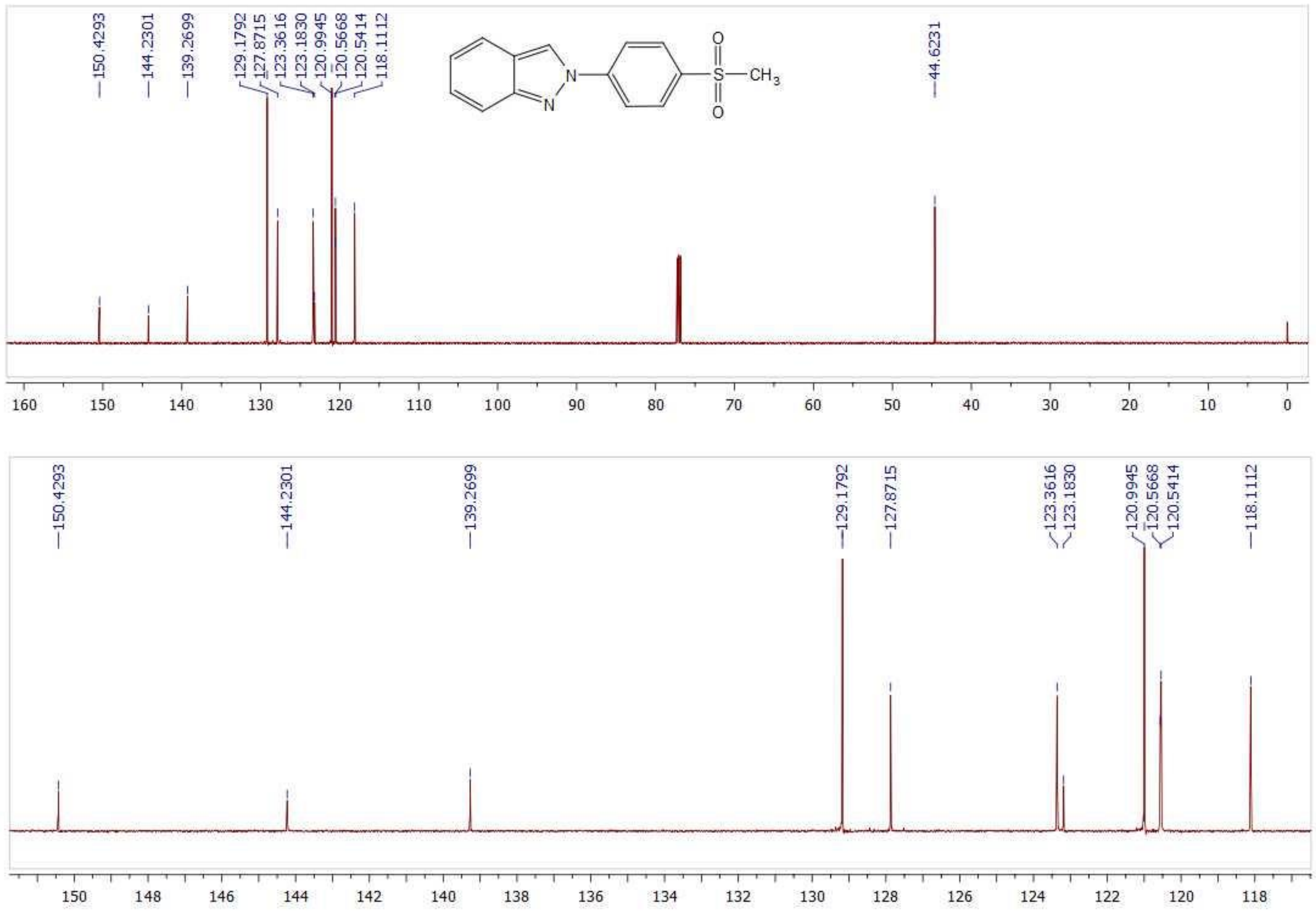

Figure S19. ^{13}C NMR (151 MHz, $CDCl_3$) for 2-(4-(methylsulfonyl)phenyl)-2*H*-indazole (**15**)

Acquisition Parameter

Source Type	ESI	Ion Polarity	Positive	Set Nebulizer	0.3 Bar
Focus	Active	Set Capillary	4500 V	Set Dry Heater	180 °C
Scan Begin	50 m/z	Set End Plate Offset	-500 V	Set Dry Gas	4.0 l/min
Scan End	3000 m/z	n/a	n/a	Set Divert Valve	Waste

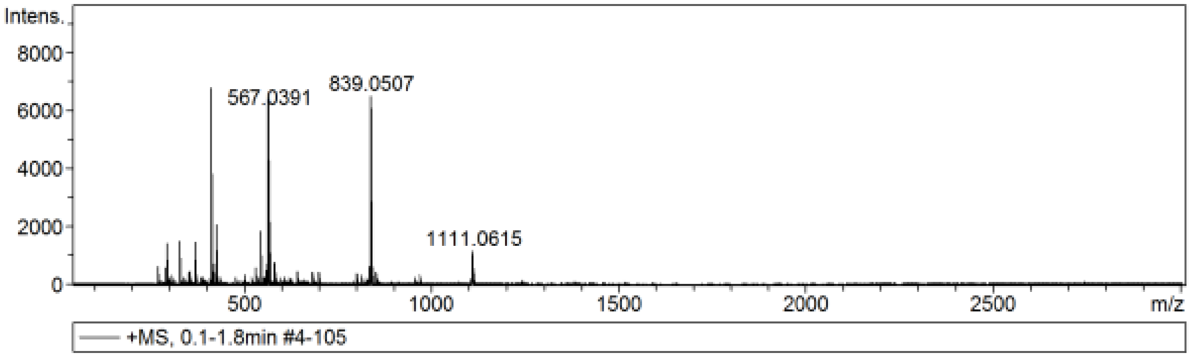

#	m/z	Res.	S/N	I	I %	FWHM
1	273.0659	3725	40.2	637	9.8	0.0733
2	292.0377	3742	31.1	502	7.7	0.0780
3	295.0428	3883	89.2	1442	22.2	0.0760
4	329.1096	3709	81.7	1371	21.1	0.0887
5	371.0088	3625	77.3	1386	21.4	0.1024
6	413.2265	3424	313.2	5902	91.0	0.1207
7	414.2294	3479	88.0	1658	25.6	0.1191
8	420.0474	3317	34.7	655	10.1	0.1266
9	420.5501	3877	26.6	502	7.7	0.1085
10	428.0360	3581	110.4	2093	32.3	0.1195
11	428.5392	3738	56.1	1064	16.4	0.1147
12	429.0370	3412	33.0	625	9.6	0.1258
13	531.1316	3255	27.6	571	8.8	0.1632
14	545.0628	3155	83.4	1732	26.7	0.1728
15	546.0637	3645	28.0	581	9.0	0.1498
16	561.1308	3876	25.7	535	8.3	0.1448
17	564.0423	3449	36.5	760	11.7	0.1636
18	564.5423	3671	26.1	543	8.4	0.1538
19	567.0391	3376	287.5	5983	92.3	0.1680
20	568.0411	3432	103.3	2149	33.1	0.1655
21	569.0414	3749	51.6	1071	16.5	0.1518
22	583.0244	2477	35.5	735	11.3	0.2353
23	836.0695	3152	35.7	618	9.5	0.2653
24	836.5590	3266	37.2	643	9.9	0.2562
25	839.0507	3358	375.4	6484	100.0	0.2499
26	840.0535	3375	193.8	3345	51.6	0.2489
27	841.0517	3184	100.8	1739	26.8	0.2641
28	842.0551	3070	37.6	648	10.0	0.2743
29	1111.0615	6065	83.8	1164	18.0	0.1832
30	1112.0707	3467	60.9	846	13.1	0.3207

Figure S20. MS (HR-ESI) for 2-(4-(methylsulfonyl)phenyl)-2*H*-indazole (**15**)

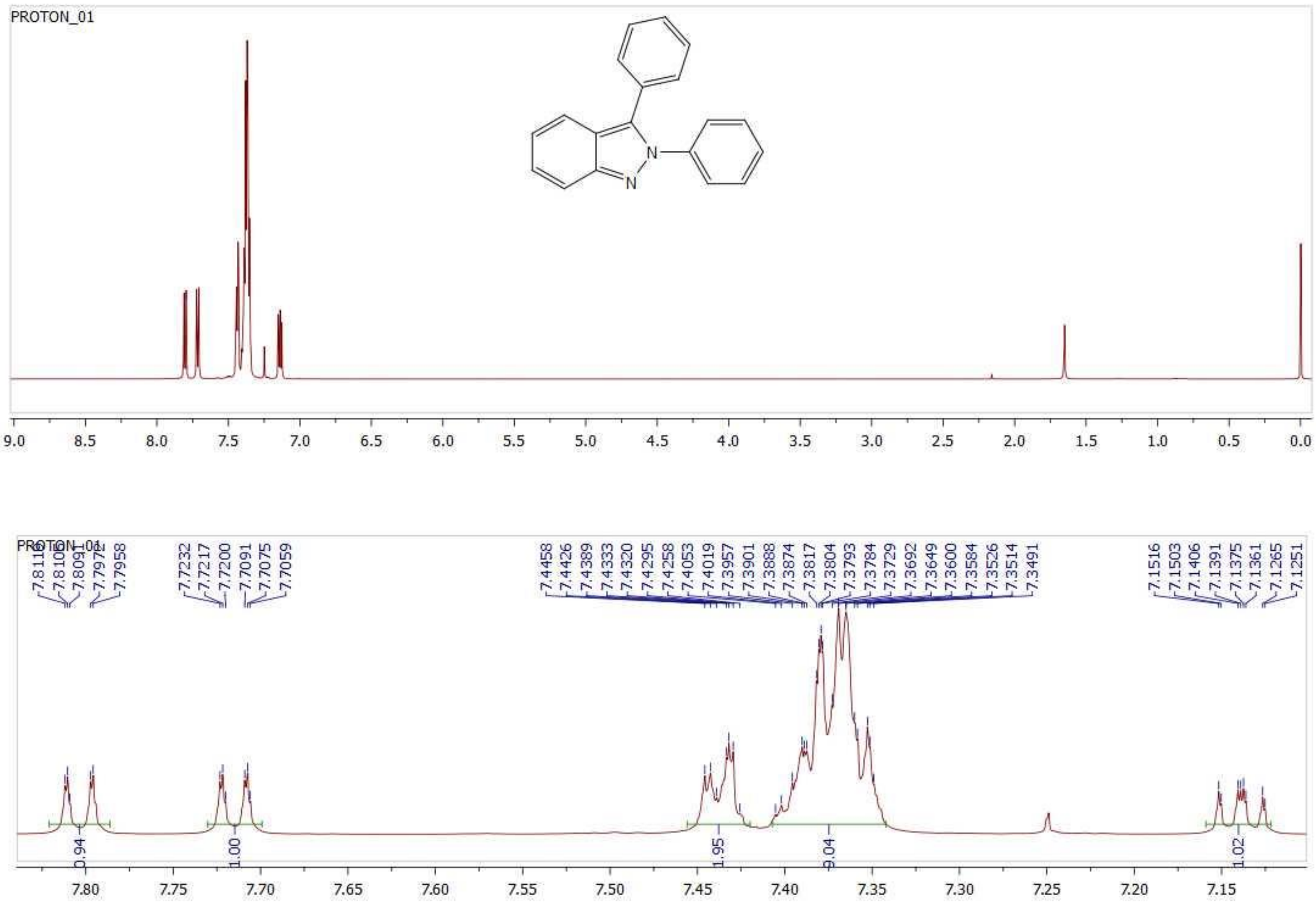

Figure S21. ^{1}H NMR (600 MHz, $CDCl_3$) for 2,3-diphenyl-2*H*-indazole (**16**)

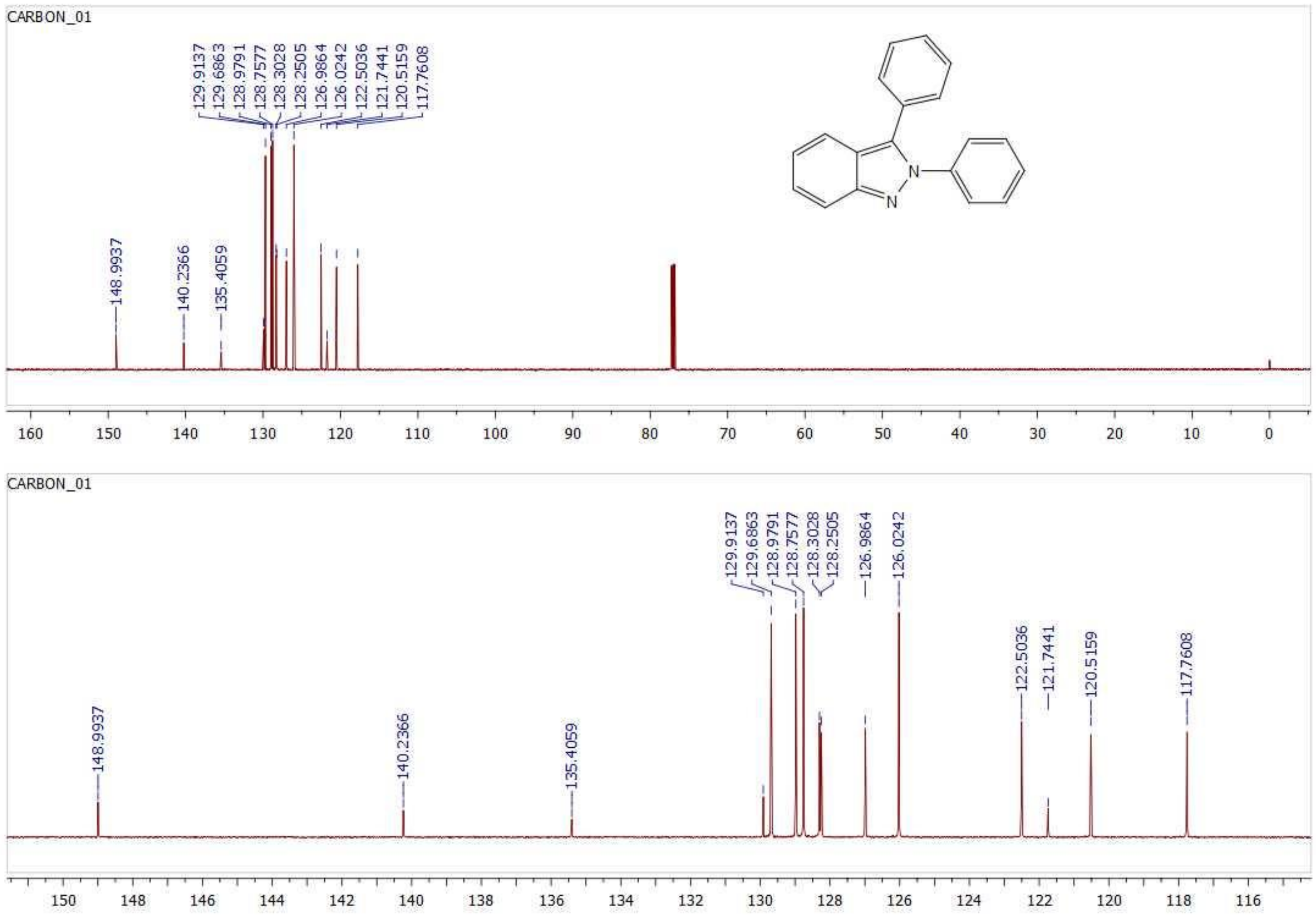

Figure S22. ^{13}C NMR (151 MHz, $CDCl_3$) for 2,3-diphenyl-2*H*-indazole (**16**)

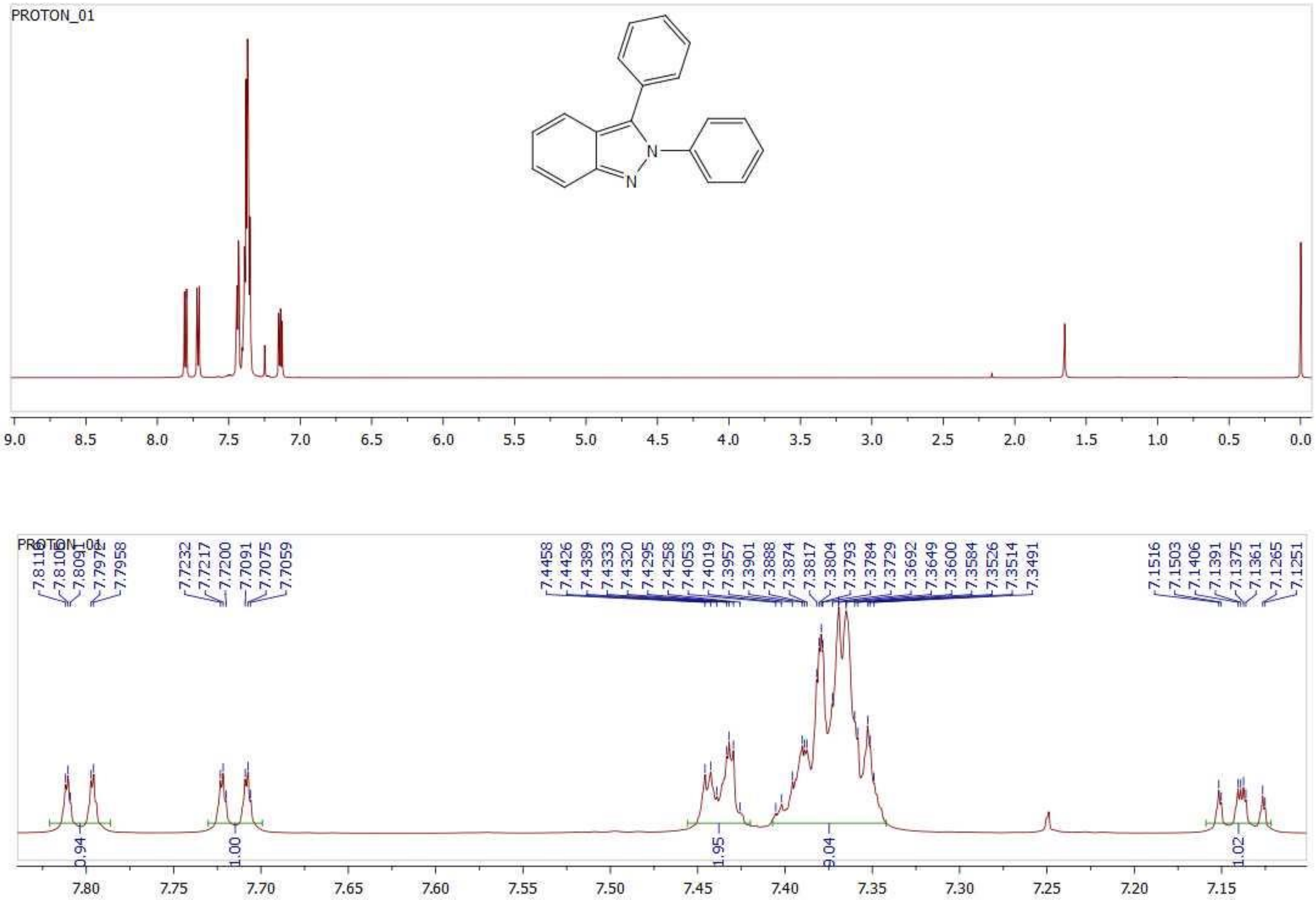

Figure S23. ^{1}H NMR (600 MHz, $CDCl_3$) for 2-(4-chlorophenyl)-3-phenyl-2*H*-indazole (**17**)

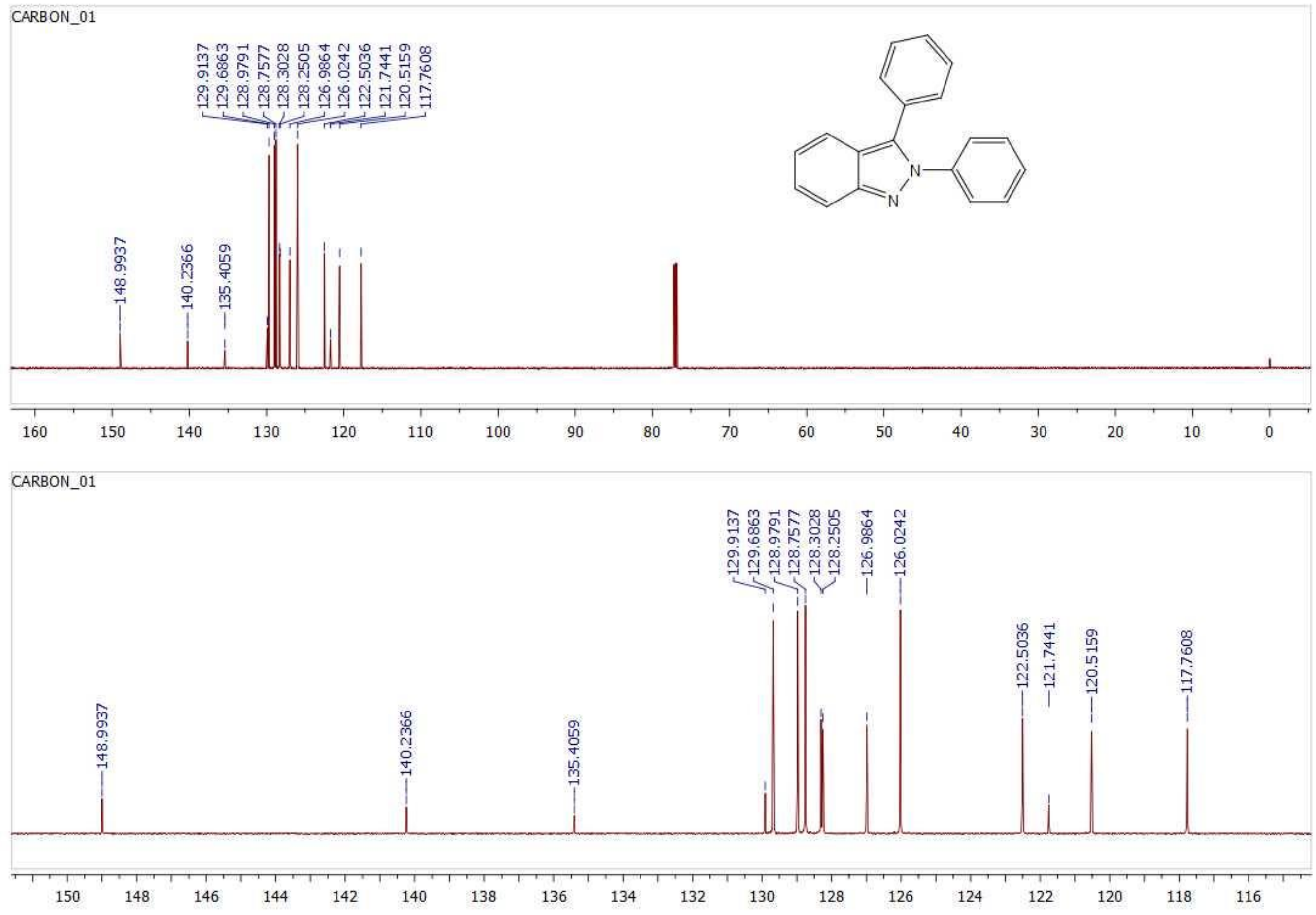

Figure S24. ^{13}C NMR (151 MHz, $CDCl_3$) for 2-(4-chlorophenyl)-3-phenyl-2*H*-indazole (**17**)

Acquisition Parameter

Source Type	ESI	Ion Polarity	Positive	Set Nebulizer	0.3 Bar
Focus	Active	Set Capillary	4500 V	Set Dry Heater	180 °C
Scan Begin	50 m/z	Set End Plate Offset	-500 V	Set Dry Gas	4.0 l/min
Scan End	3000 m/z	Set Charging Voltage	0 V	Set Divert Valve	Waste
		Set Corona	0 nA	Set APCI Heater	0 °C

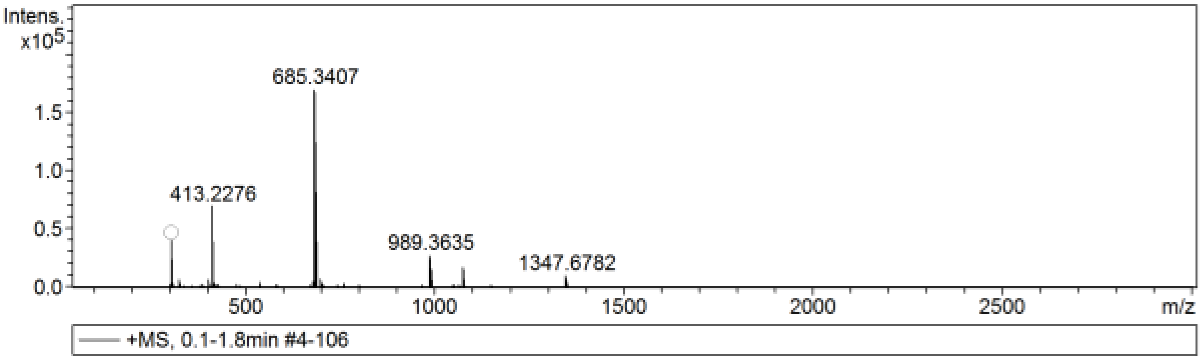

#	m/z	Res.	S/N	I	I %	FWHM
1	305.0736	3898	1148.1	40583	23.9	0.0783
2	306.0766	3834	284.5	10055	5.9	0.0798
3	307.0713	3773	435.6	15423	9.1	0.0814
4	308.0733	3500	92.7	3297	1.9	0.0880
5	327.0496	3613	169.1	6886	4.1	0.0905
6	403.0243	3026	122.8	7280	4.3	0.1332
7	405.0180	3001	49.4	2948	1.7	0.1349
8	413.2276	3854	1129.5	69304	40.8	0.1072
9	414.2312	3554	331.4	20338	12.0	0.1166
10	415.2342	3425	57.4	3531	2.1	0.1213
11	537.8206	3299	46.0	3435	2.0	0.1630
12	683.0005	6986	56.6	4491	2.6	0.0978
13	683.4643	4540	40.5	3215	1.9	0.1506
14	685.3407	6814	2140.3	169657	100.0	0.1006
15	686.3422	4418	1238.5	98127	57.8	0.1553
16	687.3471	3407	367.1	29087	17.1	0.2017
17	688.3482	3302	69.6	5508	3.2	0.2085
18	701.3140	3010	59.7	4714	2.8	0.2330
19	761.3157	3668	37.9	2891	1.7	0.2075
20	989.3635	3427	449.6	27753	16.4	0.2887
21	990.3654	3419	320.7	19789	11.7	0.2896
22	991.3661	3163	255.2	15742	9.3	0.3135
23	992.3683	3371	138.5	8539	5.0	0.2943
24	993.3799	3196	45.9	2831	1.7	0.3109
25	1075.5493	3268	311.1	17360	10.2	0.3291
26	1076.5508	3453	244.9	13656	8.0	0.3117
27	1077.5574	3338	99.0	5519	3.3	0.3228
28	1347.6782	3338	229.1	10135	6.0	0.4037
29	1348.6908	3812	213.0	9424	5.6	0.3538
30	1349.6831	3420	108.9	4821	2.8	0.3946

Figure S25. MS (HR-ESI) for 2-(4-chlorophenyl)-3-phenyl-2*H*-indazole (**17**)

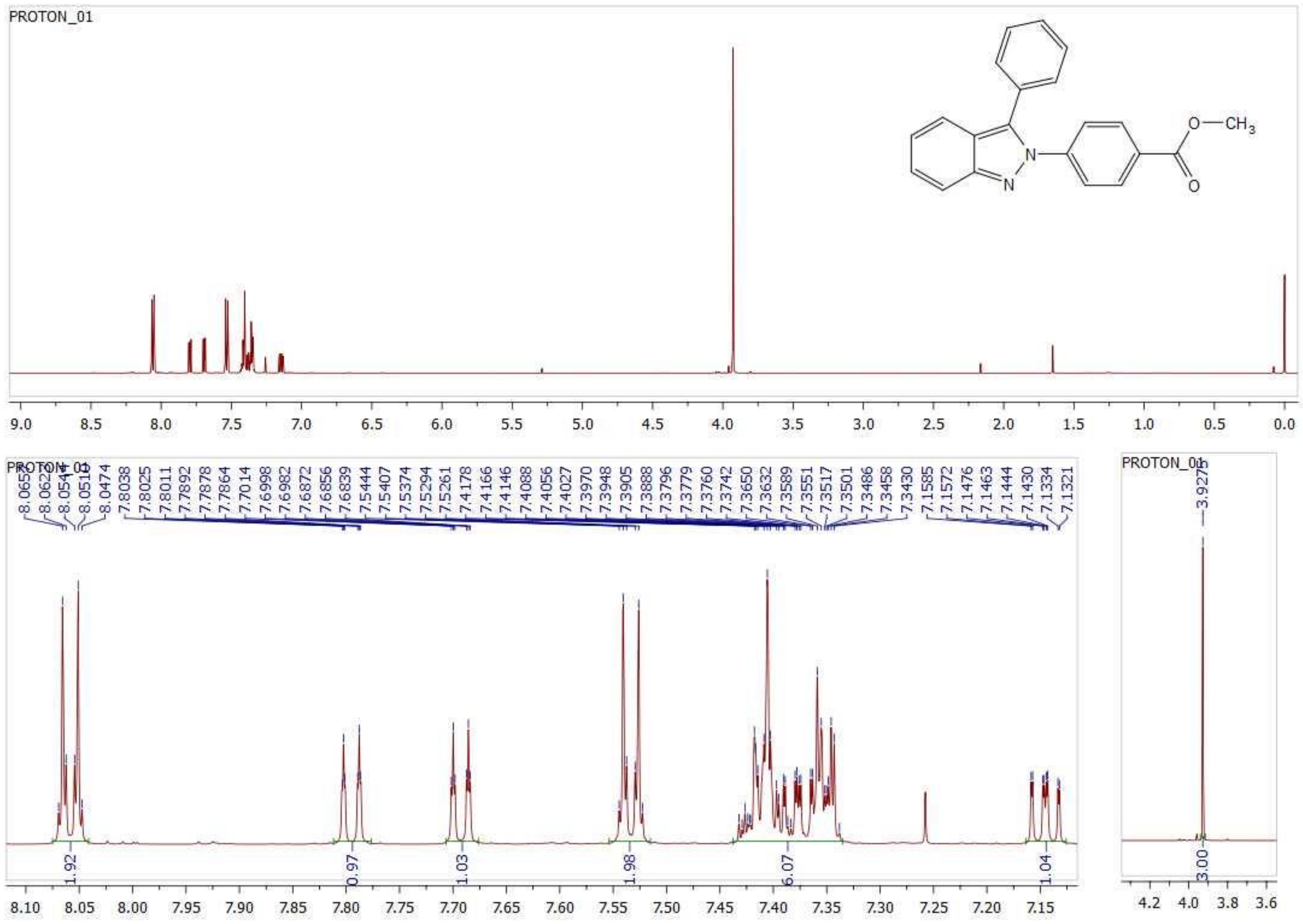

Figure S26. ^{1}H NMR (600 MHz, $CDCl_3$) for methyl 4-(3-phenyl-2*H*-indazol-2-yl)benzoate (**18**)

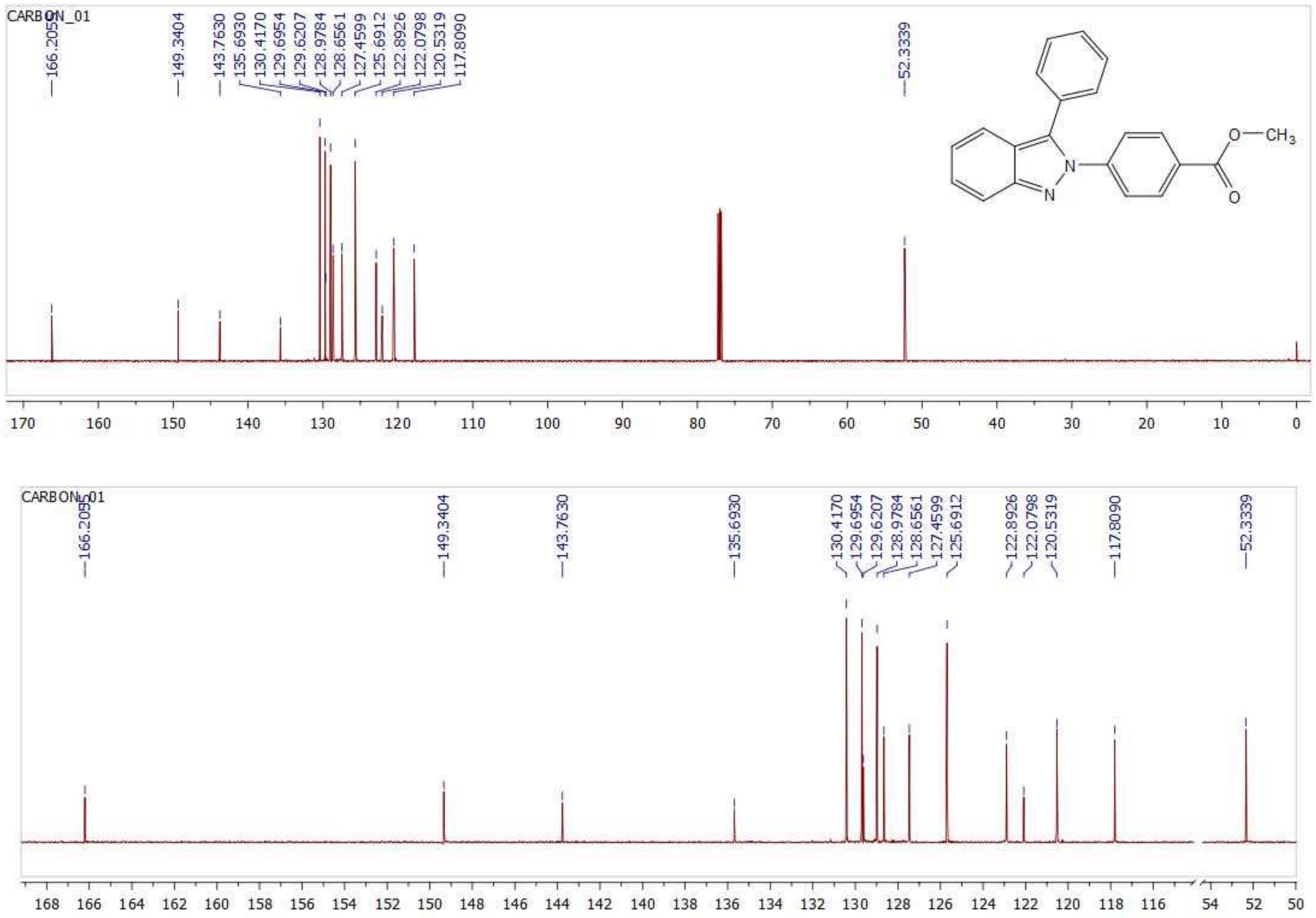

Figure S27. ^{13}C NMR (151 MHz, $CDCl_3$) for methyl 4-(3-phenyl-2*H*-indazol-2-yl)benzoate (**18**)

Acquisition Parameter

Source Type	ESI	Ion Polarity	Positive	Set Nebulizer	0.3 Bar
Focus	Active	Set Capillary	4500 V	Set Dry Heater	180 °C
Scan Begin	50 m/z	Set End Plate Offset	-500 V	Set Dry Gas	4.0 l/min
Scan End	3000 m/z	Set Charging Voltage	0 V	Set Divert Valve	Waste
		Set Corona	0 nA	Set APCI Heater	0 °C

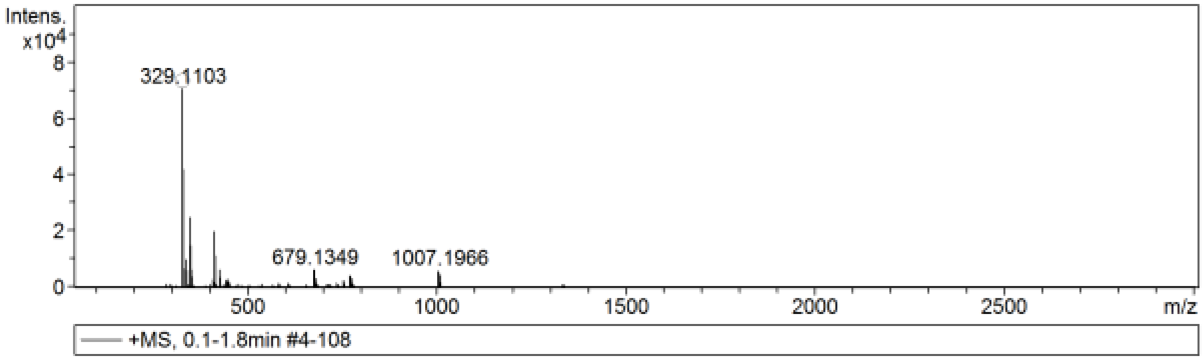

#	m/z	Res.	S/N	I	I %	FWHM
1	329.1103	4178	2684.9	70530	100.0	0.0788
2	330.1131	3817	685.2	18075	25.6	0.0865
3	331.1159	3588	97.4	2583	3.7	0.0923
4	339.1086	3782	365.7	10105	14.3	0.0897
5	340.1115	3731	101.6	2822	4.0	0.0912
6	351.0866	3644	844.8	24471	34.7	0.0963
7	352.0894	3577	212.7	6182	8.8	0.0984
8	411.0771	3482	54.6	1968	2.8	0.1181
9	413.2247	3503	488.4	17695	25.1	0.1180
10	414.2282	3471	134.9	4892	6.9	0.1194
11	427.0572	3192	165.7	6221	8.8	0.1338
12	428.0609	3170	43.3	1629	2.3	0.1350
13	443.0129	3053	38.3	1491	2.1	0.1451
14	443.9959	3152	40.0	1558	2.2	0.1409
15	445.0005	3193	64.5	2518	3.6	0.1393
16	446.9941	3282	46.0	1803	2.6	0.1362
17	451.0149	3387	59.2	2338	3.3	0.1332
18	679.1349	3480	147.3	6316	9.0	0.1951
19	680.1380	3252	70.9	3037	4.3	0.2091
20	755.1073	3303	61.7	2403	3.4	0.2286
21	771.0565	3288	39.7	1513	2.1	0.2345
22	772.0489	3277	72.4	2757	3.9	0.2356
23	773.0489	3431	103.6	3942	5.6	0.2253
24	774.0527	3176	47.3	1798	2.5	0.2437
25	775.0468	3389	81.1	3084	4.4	0.2287
26	776.0507	3278	39.1	1487	2.1	0.2368
27	777.0443	4116	40.9	1556	2.2	0.1888
28	1007.1966	3445	247.4	6171	8.7	0.2924
29	1008.2003	3313	180.1	4488	6.4	0.3043
30	1009.2018	3294	69.0	1720	2.4	0.3064

Figure S28. MS (HR-ESI) for methyl 4-(3-phenyl-2*H*-indazol-2-yl)benzoate (**18**)

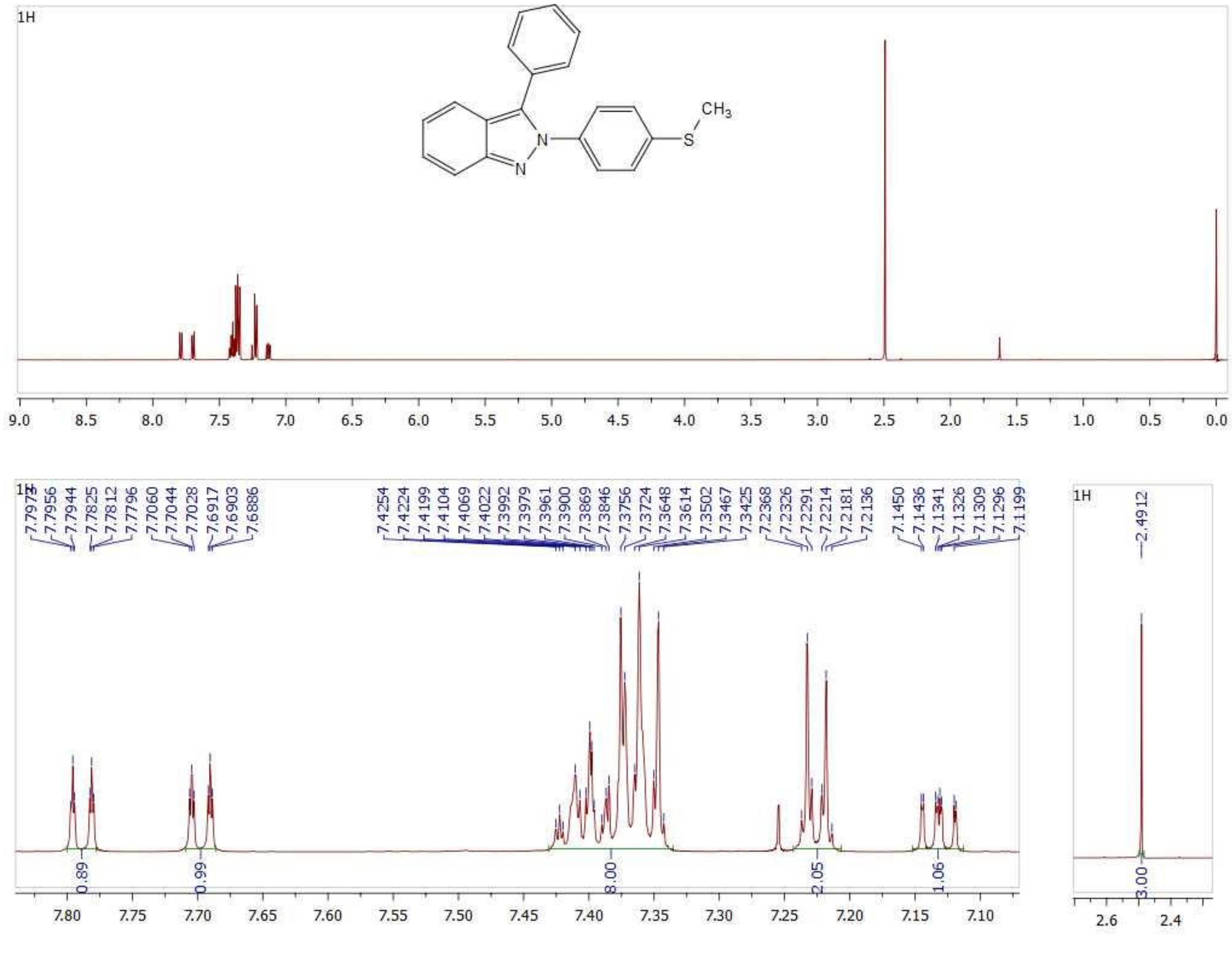

Figure S29. ^{1}H NMR (600 MHz, $CDCl_3$) for 2-(4-(methylthio)phenyl)-3-phenyl-2*H*-indazole (**19**)

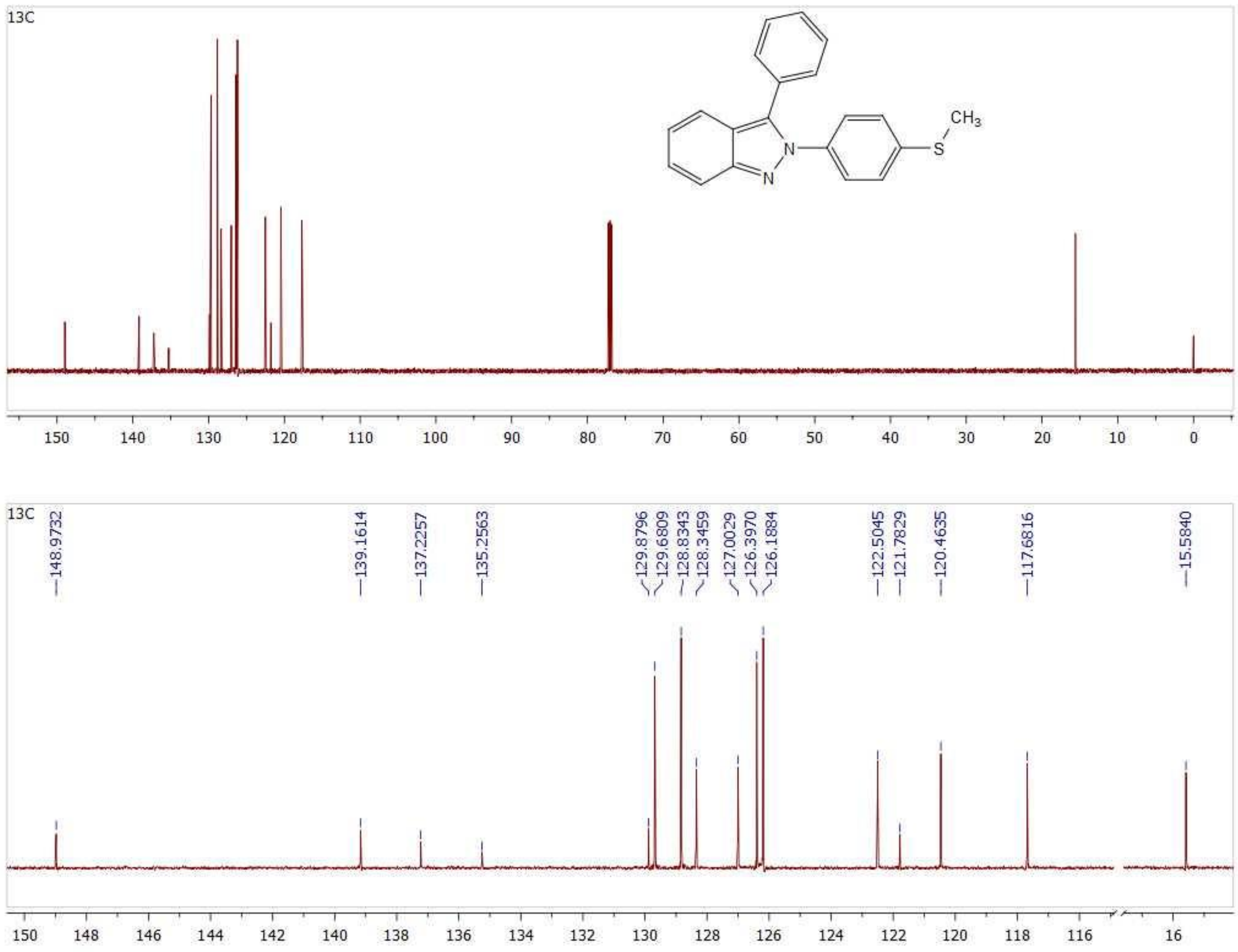

Figure S30. ^{13}C NMR (151 MHz, $CDCl_3$) for 2-(4-(methylthio)phenyl)-3-phenyl-2*H*-indazole (**19**)

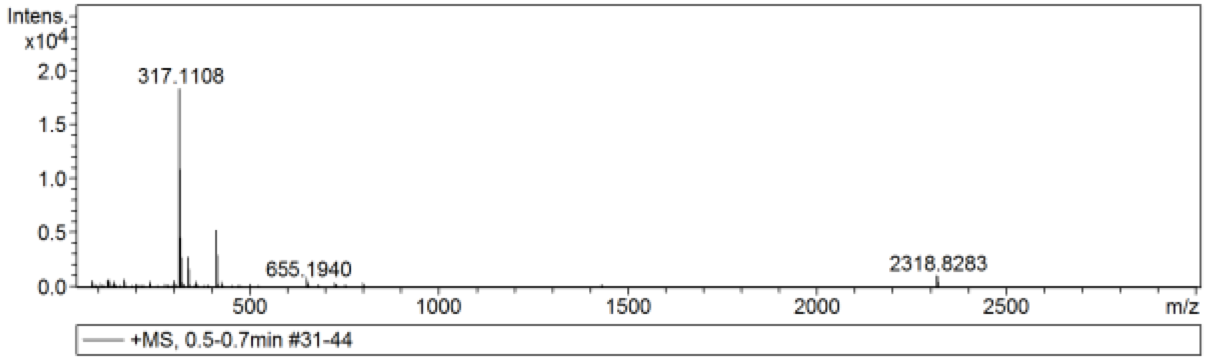

#	m/z	Res.	S/N	I	I %	FWHM
1	84.9589	7359	380.2	651	3.6	0.0115
2	110.0604	6192	155.3	316	1.7	0.0178
3	127.0247	5823	260.5	618	3.4	0.0218
4	129.0500	6583	324.2	780	4.3	0.0196
5	143.0008	5242	126.4	347	1.9	0.0273
6	172.0945	7519	223.1	756	4.1	0.0229
7	202.1810	8262	75.5	297	1.6	0.0245
8	239.1609	8421	97.1	428	2.3	0.0284
9	279.2280	4603	63.1	312	1.7	0.0607
10	301.1501	5597	128.0	662	3.6	0.0538
11	307.2588	5681	50.0	262	1.4	0.0541
12	317.1108	7524	3429.8	18294	100.0	0.0421
13	318.1139	6515	860.9	4597	25.1	0.0488
14	319.1095	6218	270.1	1444	7.9	0.0513
15	320.1119	7356	54.5	292	1.6	0.0435
16	339.0926	7327	508.3	2820	15.4	0.0463
17	340.0964	6920	121.7	676	3.7	0.0491
18	360.3228	6414	74.4	423	2.3	0.0562
19	413.2658	7186	899.4	5325	29.1	0.0575
20	414.2708	7607	252.3	1494	8.2	0.0545
21	415.0449	6433	59.2	350	1.9	0.0645
22	415.2684	10601	51.1	302	1.7	0.0392
23	429.2420	6700	61.6	366	2.0	0.0641
24	501.3130	7860	51.1	304	1.7	0.0638
25	655.1940	7534	101.3	533	2.9	0.0870
26	729.3722	6008	62.2	287	1.6	0.1214
27	803.5394	6024	63.2	250	1.4	0.1334
28	2318.8283	70246	1069.1	1072	5.9	0.0330
29	2319.0078	36976	544.9	546	3.0	0.0627
30	2319.2224	71003	488.3	489	2.7	0.0327

Figure S31. MS (HR-ESI) for 2-(4-(methylthio)phenyl)-3-phenyl-2*H*-indazole (**19**)

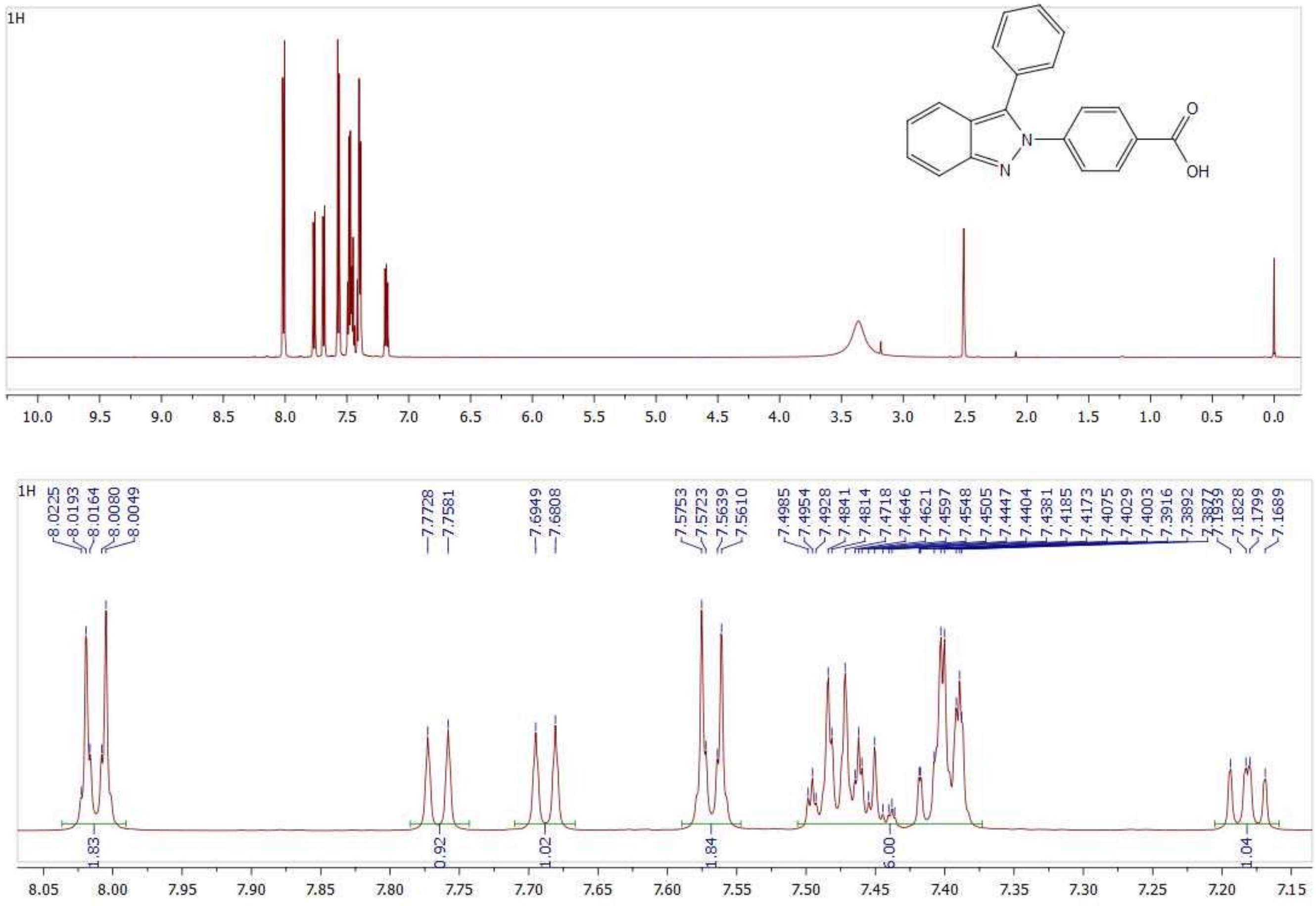

Figure S32. ¹H NMR (600 MHz, DMSO-d_6) for 4-(3-phenyl-2*H*-indazol-2-yl)benzoic acid (**20**)

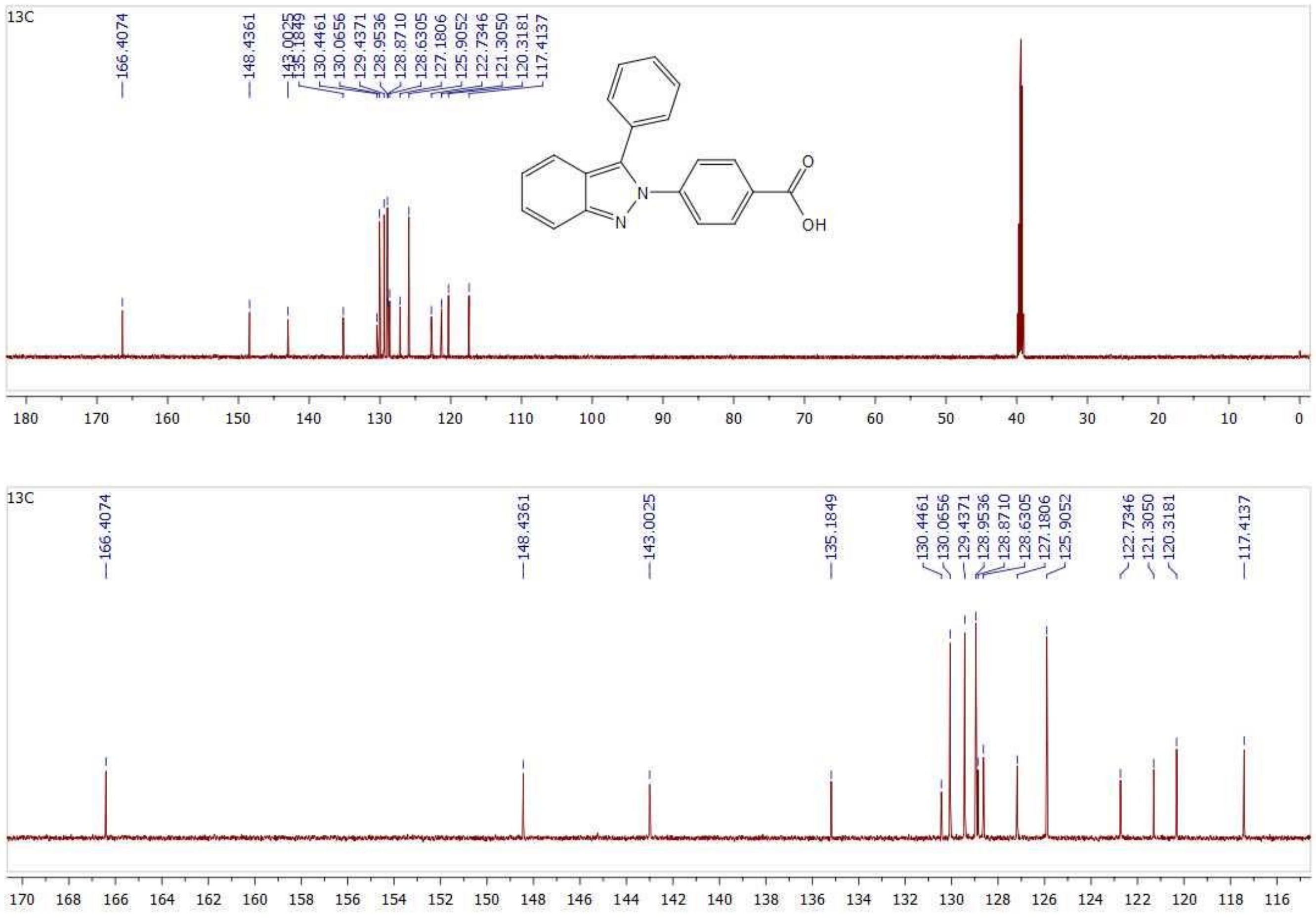

Figure S33. ^{13}C NMR (151 MHz, DMSO-d_6) for 4-(3-phenyl-2*H*-indazol-2-yl)benzoic acid (**20**)

Acquisition Parameter

Source Type	ESI	Ion Polarity	Positive	Set Nebulizer	0.5 Bar
Focus	Active	Set Capillary	4500 V	Set Dry Heater	150 °C
Scan Begin	50 m/z	Set End Plate Offset	-500 V	Set Dry Gas	4.0 l/min
Scan End	3000 m/z	Set Charging Voltage	0 V	Set Divert Valve	Waste
		Set Corona	0 nA	Set APCI Heater	0 °C

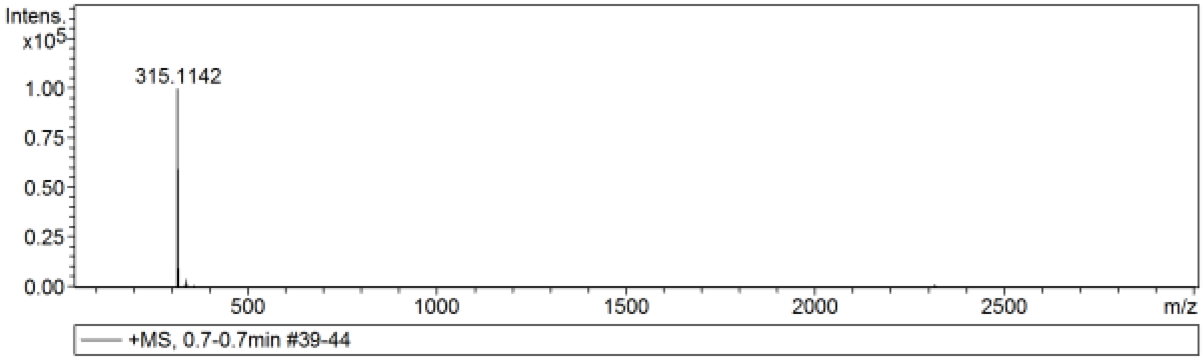

#	m/z	Res.	S/N	I	I %	FWHM
1	131.9634	3693	31.6	217	0.2	0.0357
2	239.0914	7175	15.8	186	0.2	0.0333
3	301.1315	8073	16.5	214	0.2	0.0373
4	314.1104	8098	12.6	166	0.2	0.0388
5	315.1142	8045	7558.2	99637	100.0	0.0392
6	315.5474	2711	17.7	233	0.2	0.1164
7	315.6779	5253	16.6	218	0.2	0.0601
8	316.1167	6871	1649.0	21744	21.8	0.0460
9	317.1210	7026	223.7	2949	3.0	0.0451
10	318.1223	8528	25.4	336	0.3	0.0373
11	321.3159	6010	19.6	261	0.3	0.0535
12	321.4892	24792	13.7	182	0.2	0.0130
13	331.1101	5633	11.4	153	0.2	0.0588
14	337.0994	6976	54.1	729	0.7	0.0483
15	338.1022	10708	15.9	214	0.2	0.0316
16	338.3433	6963	215.2	2903	2.9	0.0486
17	339.3441	6403	46.7	630	0.6	0.0530
18	340.3498	9880	12.9	174	0.2	0.0344
19	360.3265	8130	79.1	1067	1.1	0.0443
20	361.3279	6830	17.3	233	0.2	0.0529
21	391.1439	7041	26.3	350	0.4	0.0556
22	408.3094	6657	11.0	145	0.1	0.0613
23	629.2196	5383	19.8	213	0.2	0.1169
24	630.2148	7787	13.1	141	0.1	0.0809
25	651.2072	10893	16.8	175	0.2	0.0598
26	755.7004	38508	22.8	192	0.2	0.0196
27	755.8128	18218	16.4	138	0.1	0.0415
28	2318.8591	69309	303.1	924	0.9	0.0335
29	2319.0402	33740	150.4	458	0.5	0.0687
30	2319.2534	71910	185.2	564	0.6	0.0323

Figure S34. MS (HR-ESI) for 4-(3-phenyl-2*H*-indazol-2-yl)benzoic acid (**20**)

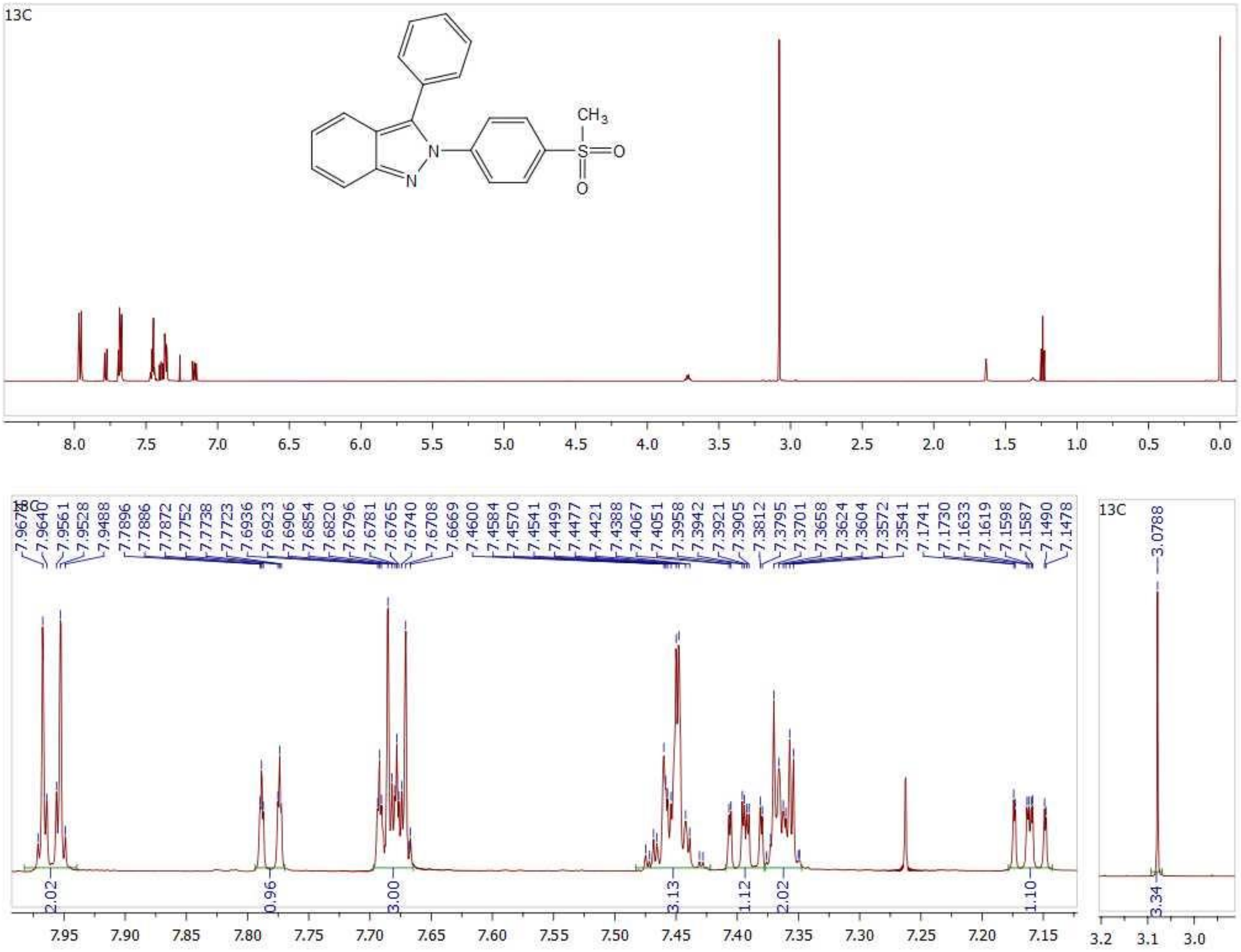

Figure S35. ^{1}H NMR (600 MHz, $CDCl_3$) for 2-(4-(methylsulfonyl)phenyl)-3-phenyl-2*H*-indazole (**21**)

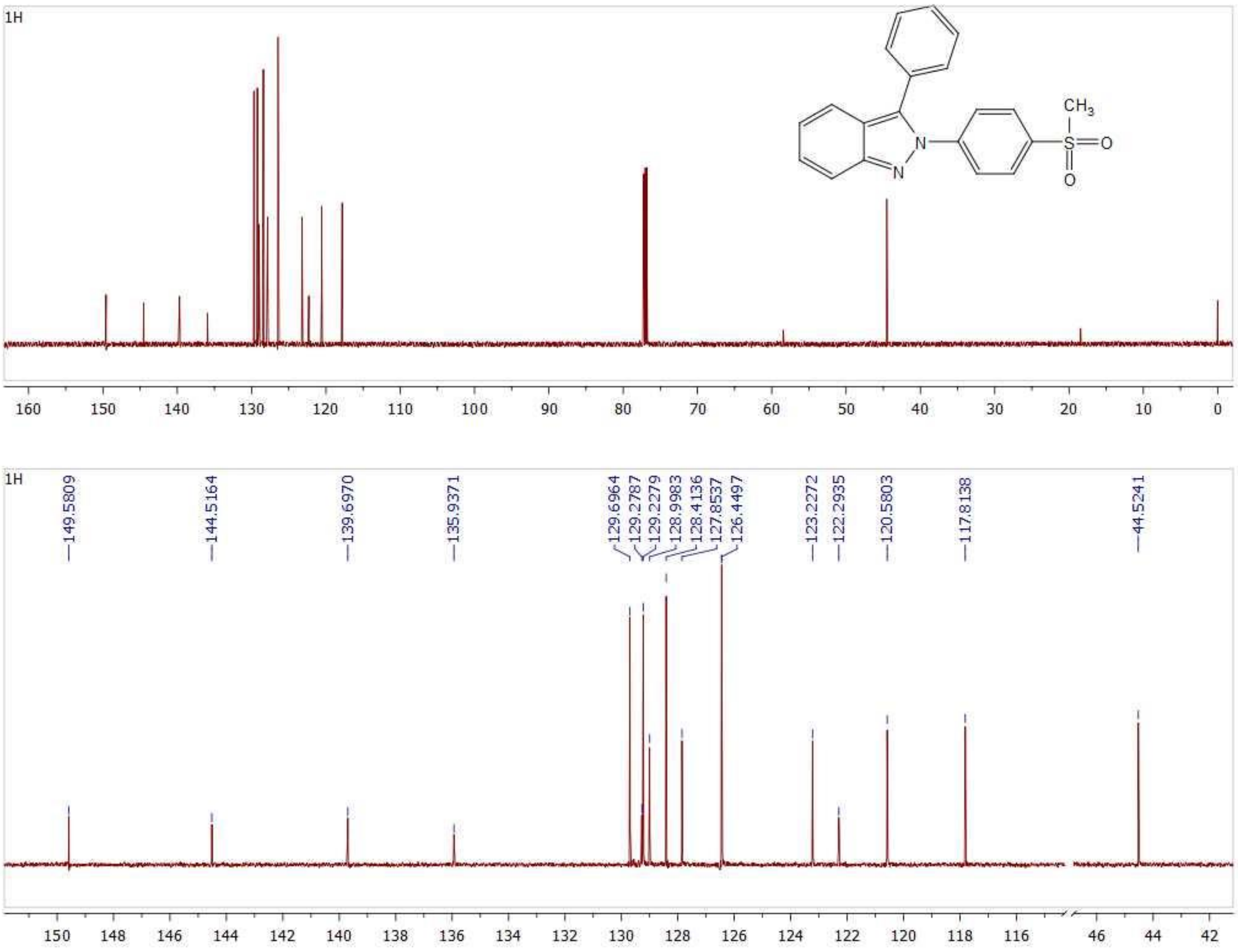

Figure S36. ^{13}C NMR (151 MHz, $CDCl_3$) for 2-(4-(methylsulfonyl)phenyl)-3-phenyl-2*H*-indazole (**21**)

Acquisition Parameter

Source Type	ESI	Ion Polarity	Positive	Set Nebulizer	0.5 Bar
Focus	Active	Set Capillary	4500 V	Set Dry Heater	150 °C
Scan Begin	50 m/z	Set End Plate Offset	-500 V	Set Dry Gas	4.0 l/min
Scan End	3000 m/z	Set Charging Voltage	0 V	Set Divert Valve	Waste
		Set Corona	0 nA	Set APCI Heater	0 °C

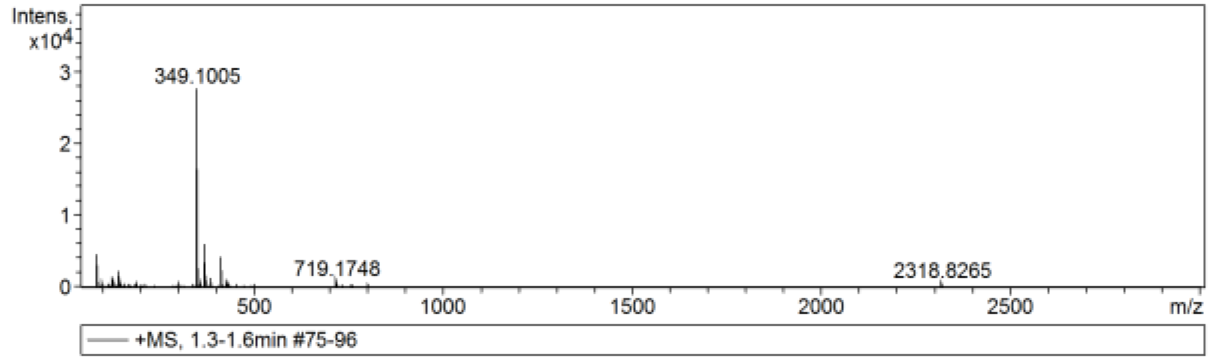

#	m/z	Res.	S/N	I	I %	FWHM
1	84.9597	6899	3841.6	4673	16.9	0.0123
2	100.9906	6562	575.7	769	2.8	0.0154
3	127.0244	6654	865.2	1470	5.3	0.0191
4	129.0491	5671	619.2	1072	3.9	0.0228
5	131.0031	5916	336.4	595	2.2	0.0221
6	143.0018	6638	1208.7	2396	8.7	0.0215
7	145.0164	5571	677.9	1367	5.0	0.0260
8	146.9962	6646	573.8	1182	4.3	0.0221
9	149.0214	5580	187.8	394	1.4	0.0267
10	158.9765	5808	211.4	470	1.7	0.0274
11	172.0933	7209	188.4	465	1.7	0.0239
12	189.0414	6331	167.4	475	1.7	0.0299
13	191.0223	4140	214.4	617	2.2	0.0461
14	301.1418	6503	134.1	629	2.3	0.0463
15	349.1005	7581	5102.6	27588	100.0	0.0461
16	350.1025	6770	1223.5	6627	24.0	0.0517
17	351.0995	6650	416.7	2260	8.2	0.0528
18	352.0997	7048	85.1	463	1.7	0.0500
19	360.3237	7643	122.6	679	2.5	0.0471
20	371.0827	6836	1086.6	6148	22.3	0.0543
21	372.0848	6614	282.2	1598	5.8	0.0563
22	373.0832	6664	94.9	538	1.9	0.0560
23	387.0558	7309	218.4	1268	4.6	0.0530
24	413.2656	7294	720.2	4331	15.7	0.0567
25	414.2706	7295	196.2	1180	4.3	0.0568
26	429.2410	6733	174.6	1063	3.9	0.0638
27	433.0507	8720	96.2	587	2.1	0.0497
28	719.1748	7693	182.2	893	3.2	0.0935
29	720.1788	6334	81.3	398	1.4	0.1137
30	2318.8265	69327	1461.2	578	2.1	0.0334

Figure S37. MS (HR-ESI) for 2-(4-(methylsulfonyl)phenyl)-3-phenyl-2*H*-indazole (**21**)

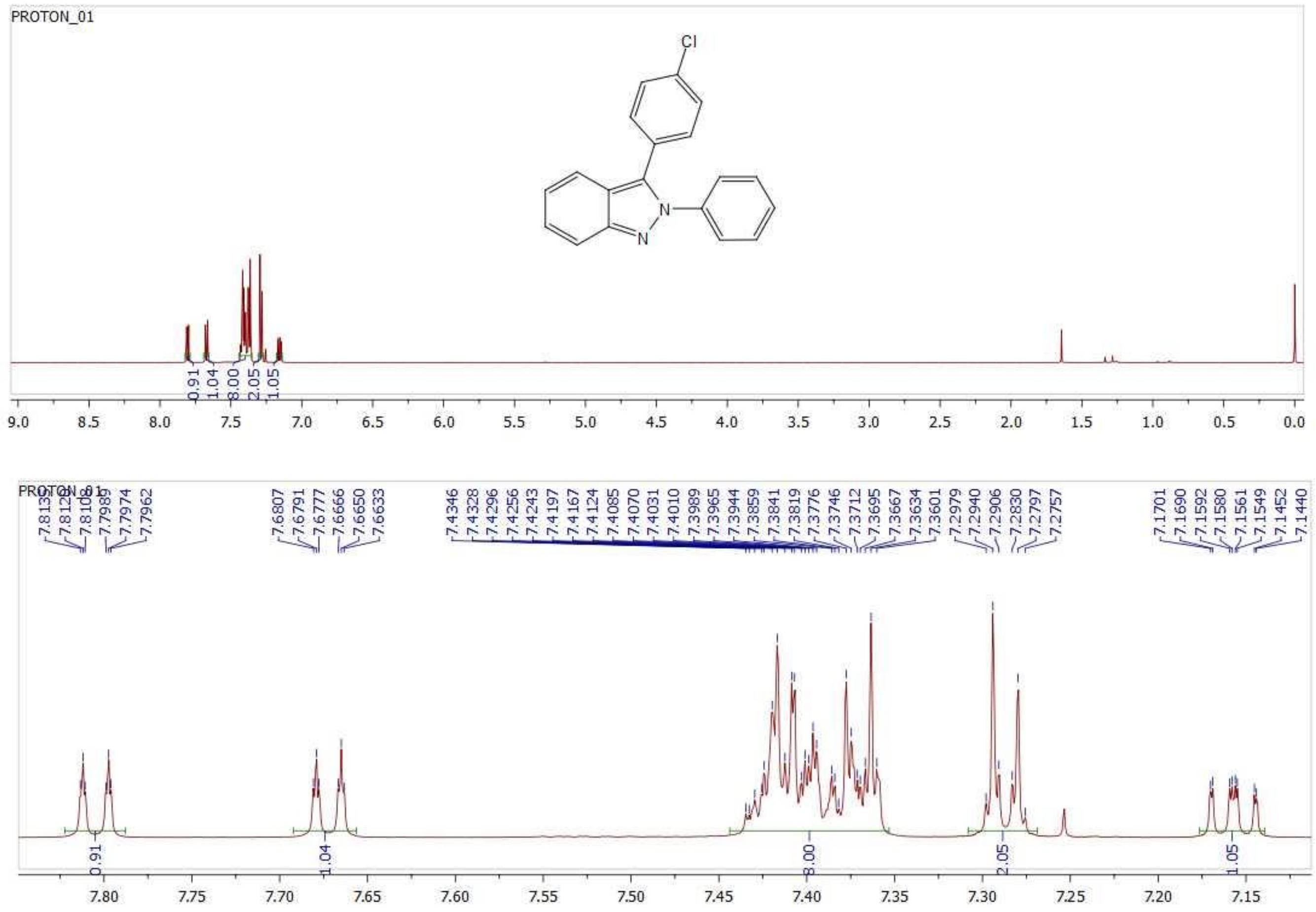

Figure S38. ^{1}H NMR (600 MHz, $CDCl_3$) for 3-(4-chlorophenyl)-2-phenyl-2*H*-indazole (**22**)

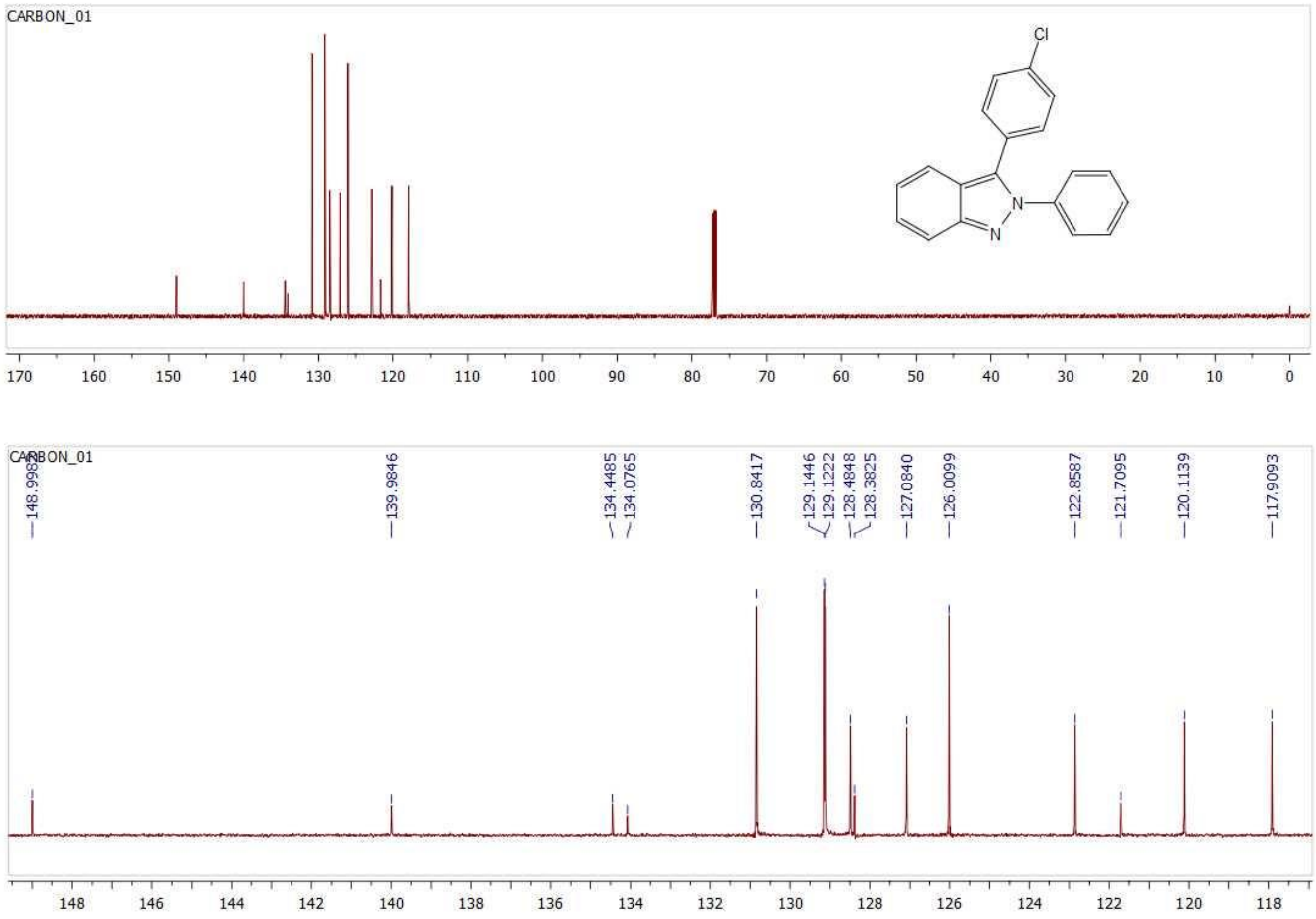

Figure S39. ^{13}C NMR (151 MHz, $CDCl_3$) for 3-(4-chlorophenyl)-2-phenyl-2*H*-indazole (**22**)

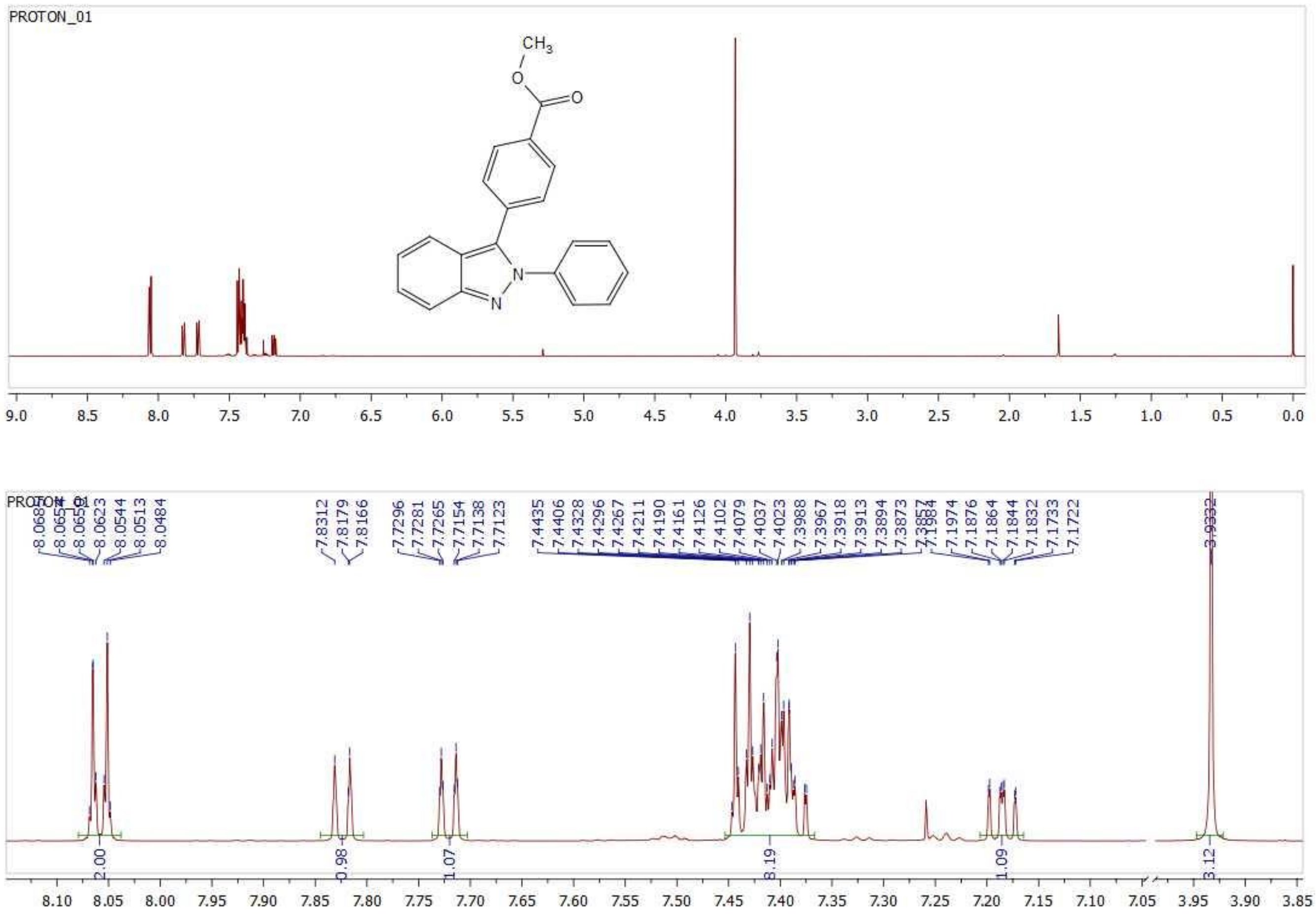

Figure S40. ^{1}H NMR (600 MHz, $CDCl_3$) for methyl 4-(2-phenyl-2*H*-indazol-3-yl)benzoate (**23**)

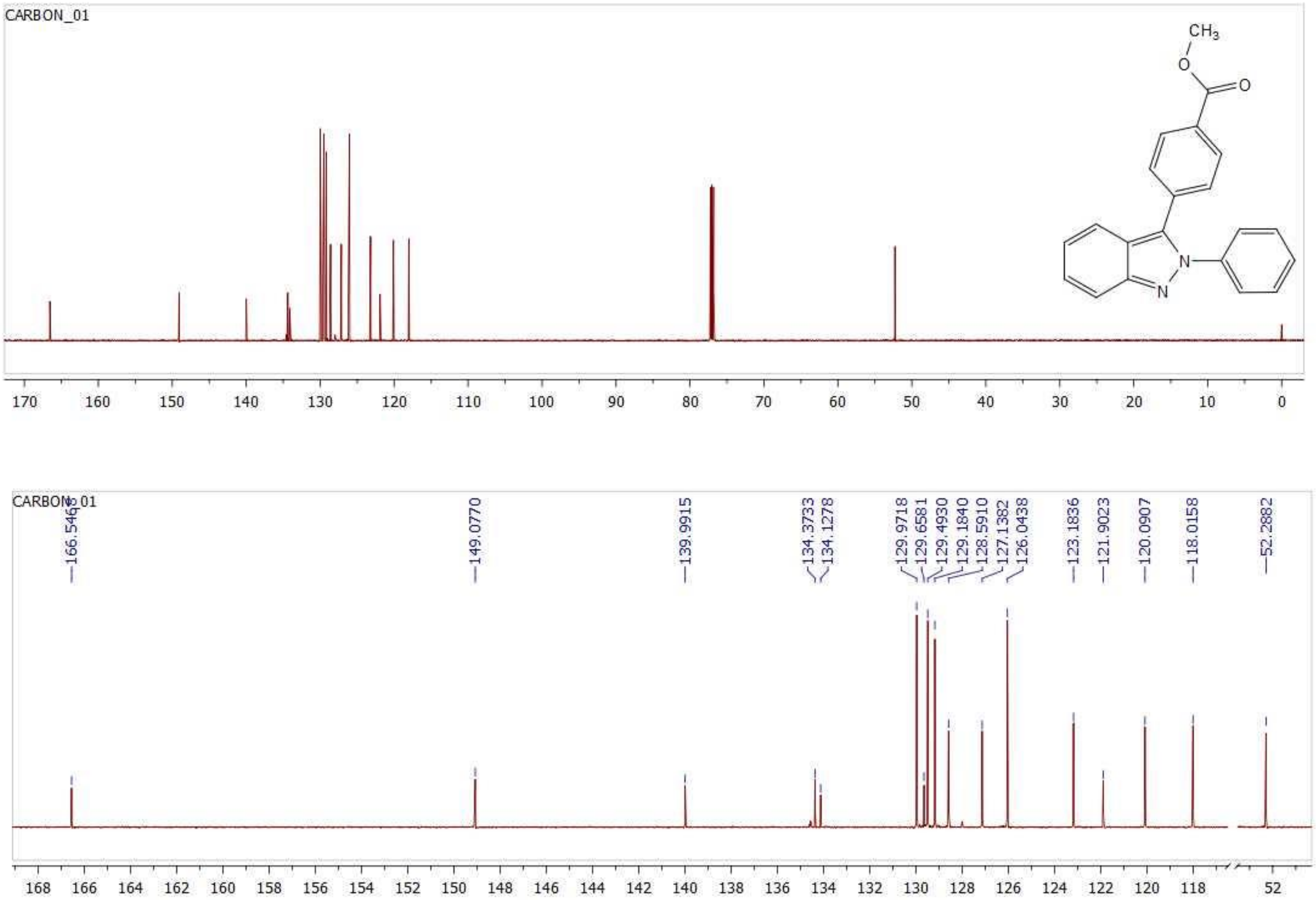

Figure S41. ^{13}C NMR (151 MHz, $CDCl_3$) for methyl 4-(2-phenyl-2*H*-indazol-3-yl)benzoate (**23**)

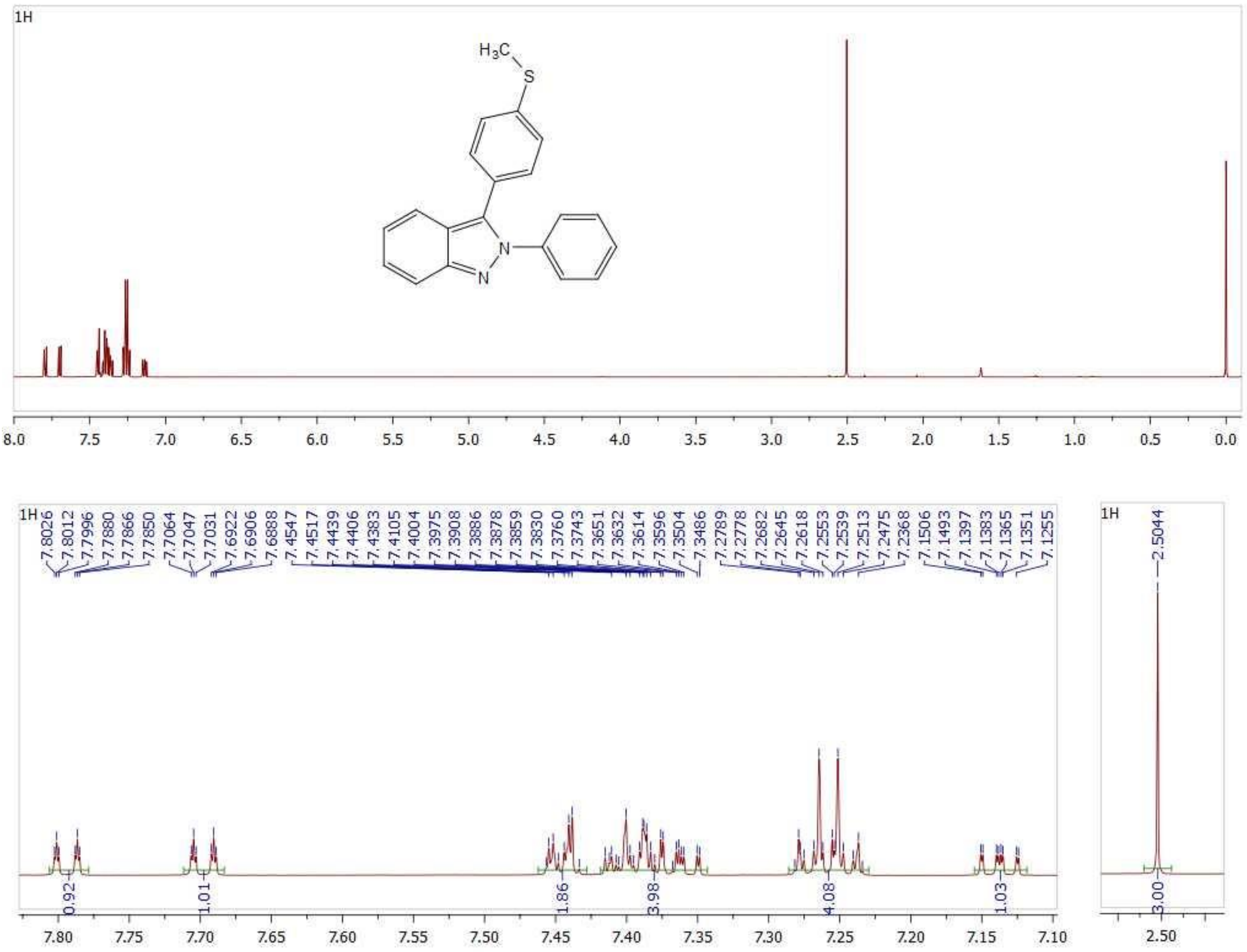

Figure S42. ^{1}H NMR (600 MHz, $CDCl_3$) for 3-(4-(methylthio)phenyl)-2-phenyl-2*H*-indazole (**24**)

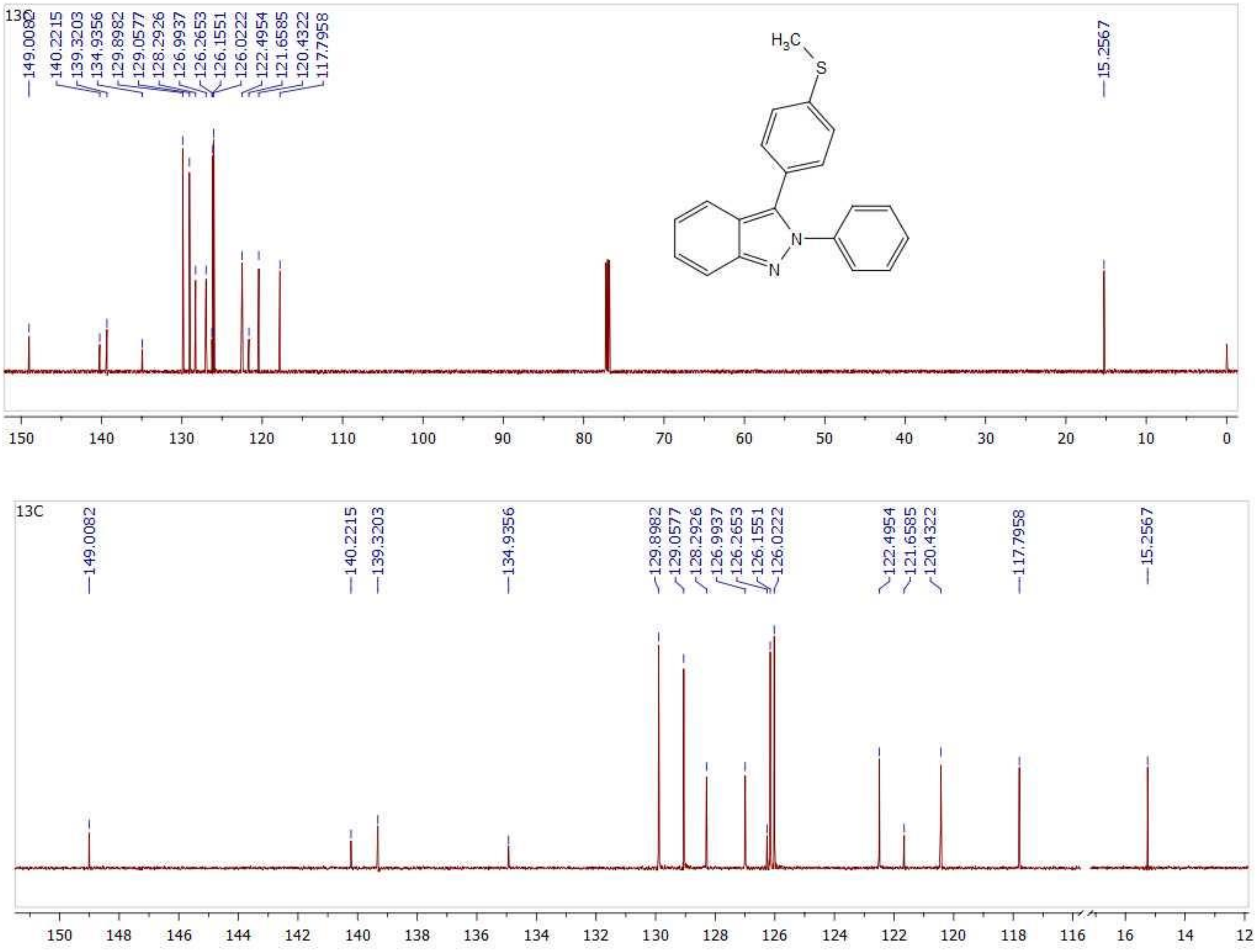

Figure S43. ^{13}C NMR (151 MHz, $CDCl_3$) for 3-(4-(methylthio)phenyl)-2-phenyl-2*H*-indazole (**24**)

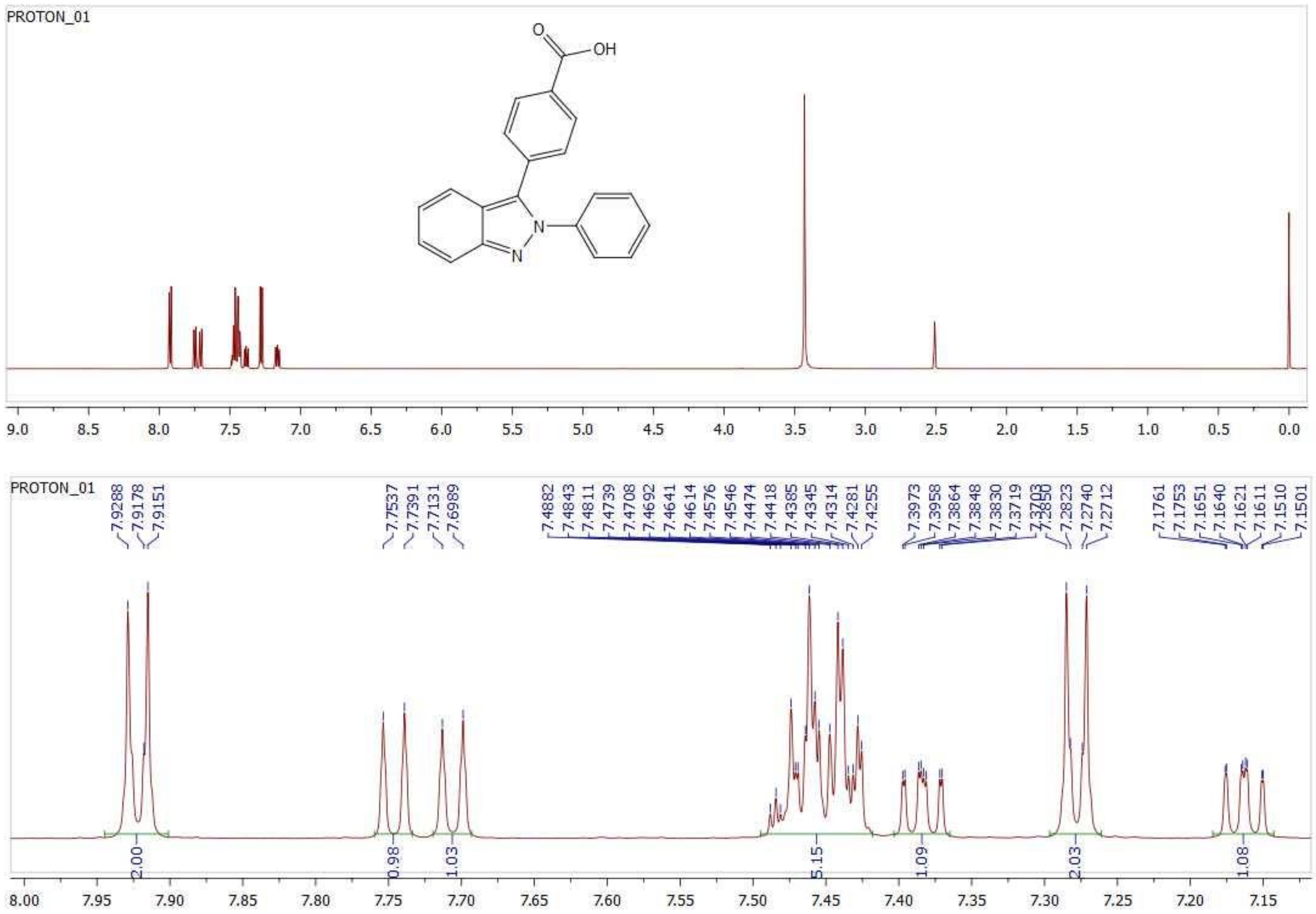

Figure S44. ^{1}H NMR (600 MHz, DMSO-d_6) for 4-(2-phenyl-2*H*-indazol-3-yl)benzoic acid (**25**)

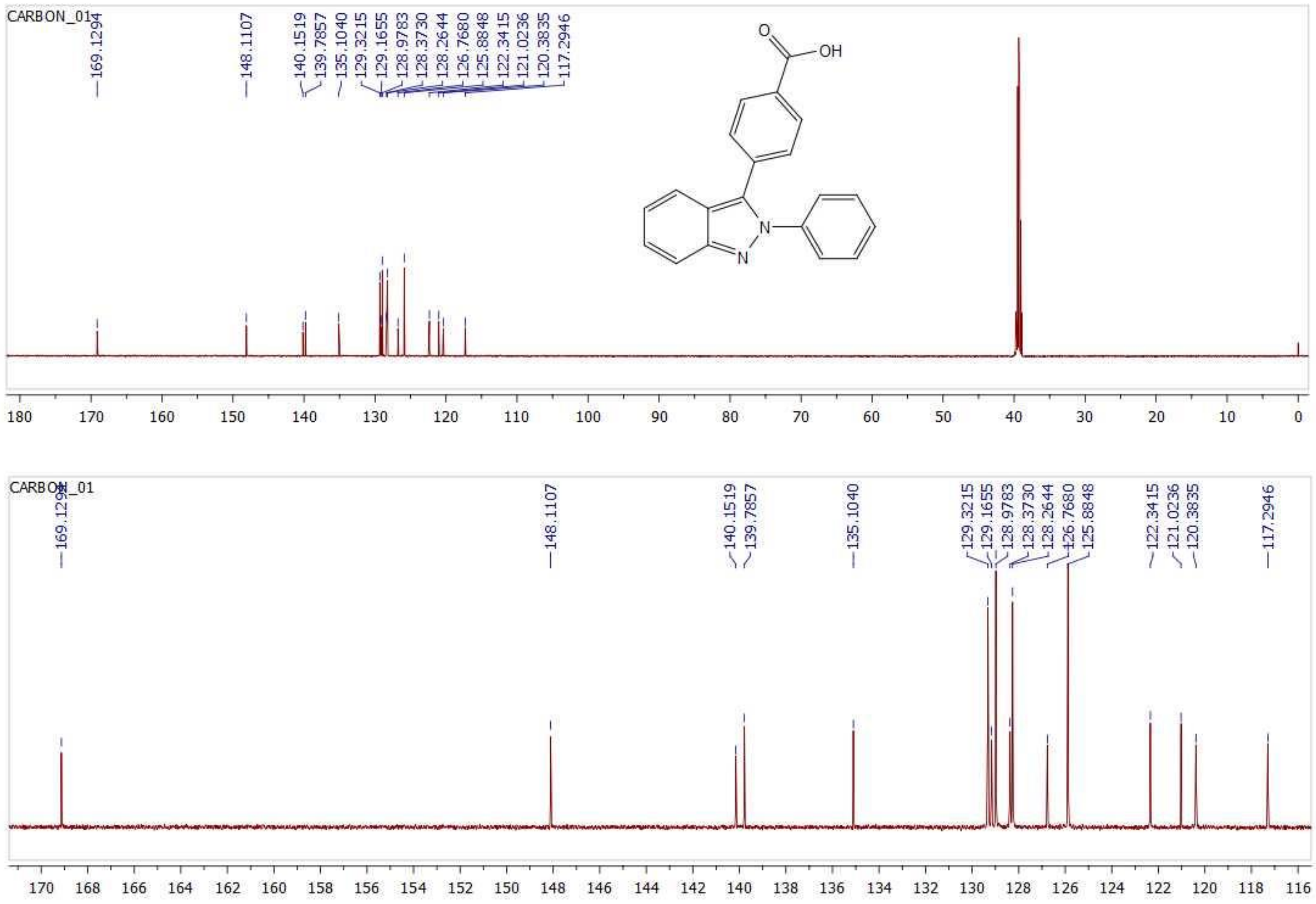

Figure S45. ^{13}C NMR (151 MHz, DMSO-d_6) for 4-(2-phenyl-2*H*-indazol-3-yl)benzoic acid (**25**)

Acquisition Parameter

Source Type	ESI	Ion Polarity	Positive	Set Nebulizer	0.5 Bar
Focus	Active	Set Capillary	4500 V	Set Dry Heater	150 °C
Scan Begin	50 m/z	Set End Plate Offset	-500 V	Set Dry Gas	4.0 l/min
Scan End	3000 m/z	Set Charging Voltage	0 V	Set Divert Valve	Waste
		Set Corona	0 nA	Set APCI Heater	0 °C

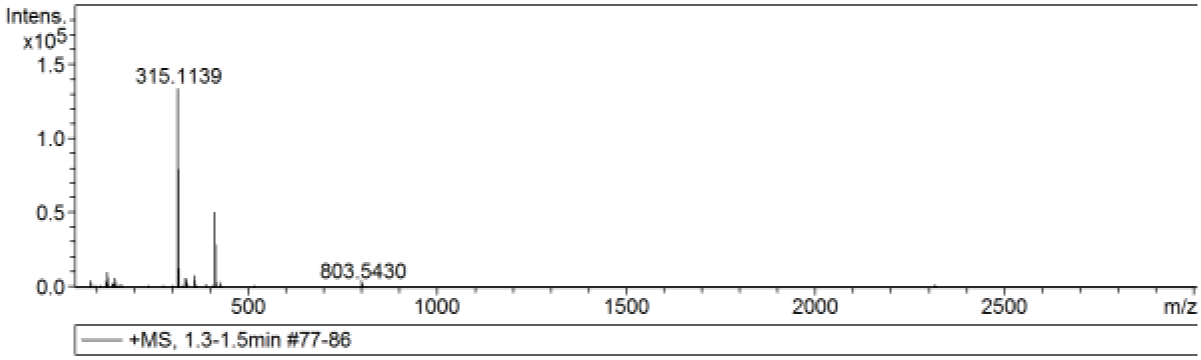

#	m/z	Res.	S/N	I	I %	FWHM
1	84.9596	7337	887.6	4628	3.5	0.0116
2	113.1337	6713	174.5	1115	0.8	0.0169
3	127.0254	7106	371.7	2664	2.0	0.0179
4	129.0528	6383	1402.3	10122	7.6	0.0202
5	143.0017	6580	282.1	2275	1.7	0.0217
6	145.0204	5072	190.8	1562	1.2	0.0286
7	146.9970	7282	184.8	1543	1.2	0.0202
8	149.0243	6878	739.2	6283	4.7	0.0217
9	167.0347	6725	179.0	1704	1.3	0.0248
10	239.1629	4577	94.3	1178	0.9	0.0523
11	279.1619	5723	69.7	976	0.7	0.0488
12	301.1432	6126	82.6	1207	0.9	0.0492
13	315.1139	7954	8941.9	133443	100.0	0.0396
14	316.1168	6877	2085.8	31157	23.3	0.0460
15	317.1171	6120	269.3	4029	3.0	0.0518
16	337.0958	7085	403.5	6260	4.7	0.0476
17	338.1000	6294	92.0	1428	1.1	0.0537
18	338.3429	7373	242.0	3759	2.8	0.0459
19	359.0775	6747	117.3	1866	1.4	0.0532
20	360.3243	7280	473.1	7527	5.6	0.0495
21	361.3279	7081	114.7	1824	1.4	0.0510
22	391.2853	6407	110.2	1787	1.3	0.0611
23	413.2668	7546	3102.1	50523	37.9	0.0548
24	414.2706	6834	857.4	13964	10.5	0.0606
25	415.2734	7782	151.7	2469	1.9	0.0534
26	429.2407	7133	190.9	3104	2.3	0.0602
27	519.3300	7270	89.6	1405	1.1	0.0714
28	803.5430	7142	278.2	2686	2.0	0.1125
29	804.5450	5721	128.3	1239	0.9	0.1406
30	2318.8436	69176	1168.5	1085	0.8	0.0335

Figure S46. MS (HR-ESI) for 4-(2-phenyl-2*H*-indazol-3-yl)benzoic acid (**25**)

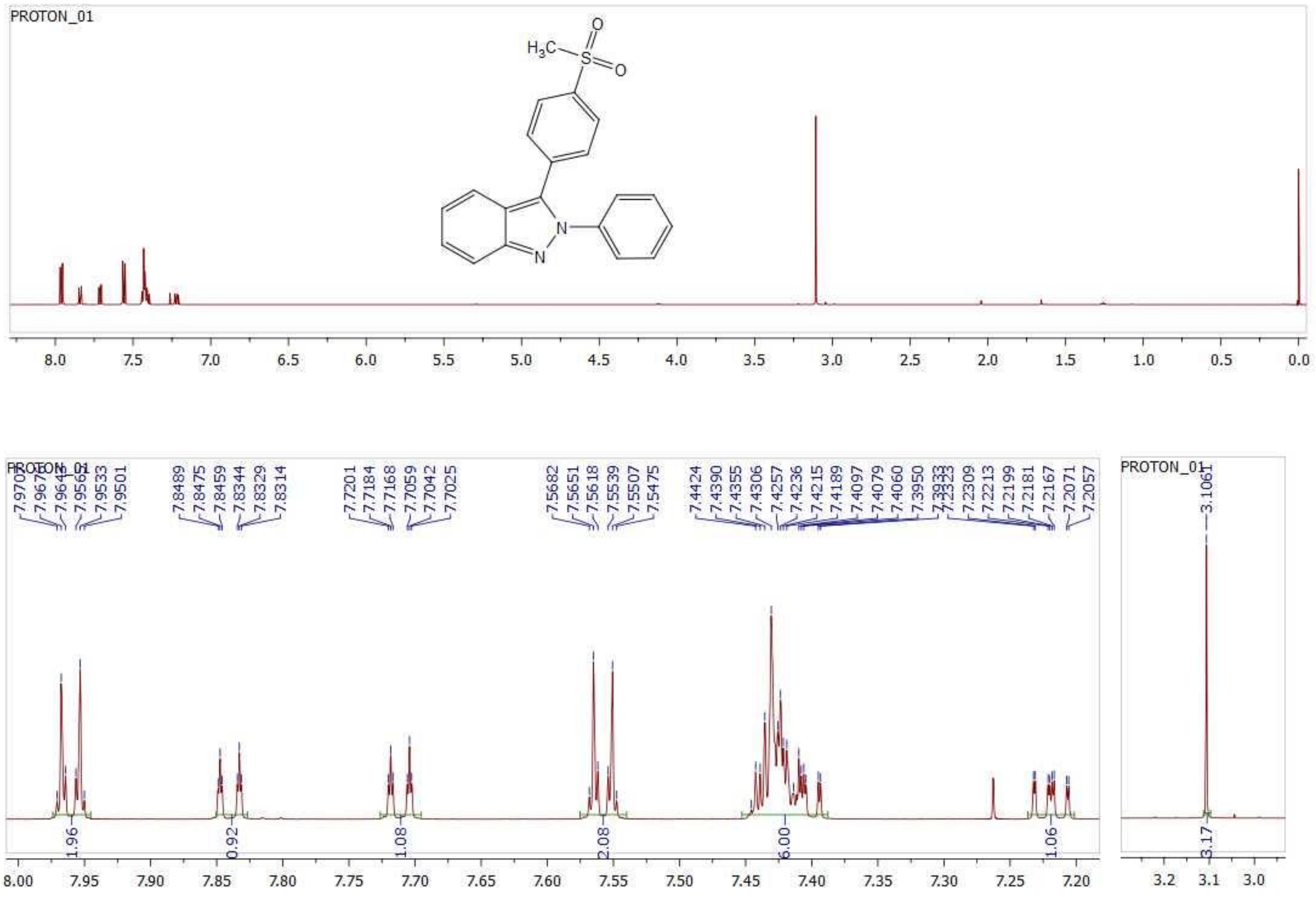

Figure S47. ^{1}H NMR (600 MHz, $CDCl_3$) for 3-(4-(methylsulfonyl)phenyl)-2-phenyl-2*H*-indazole (**26**)

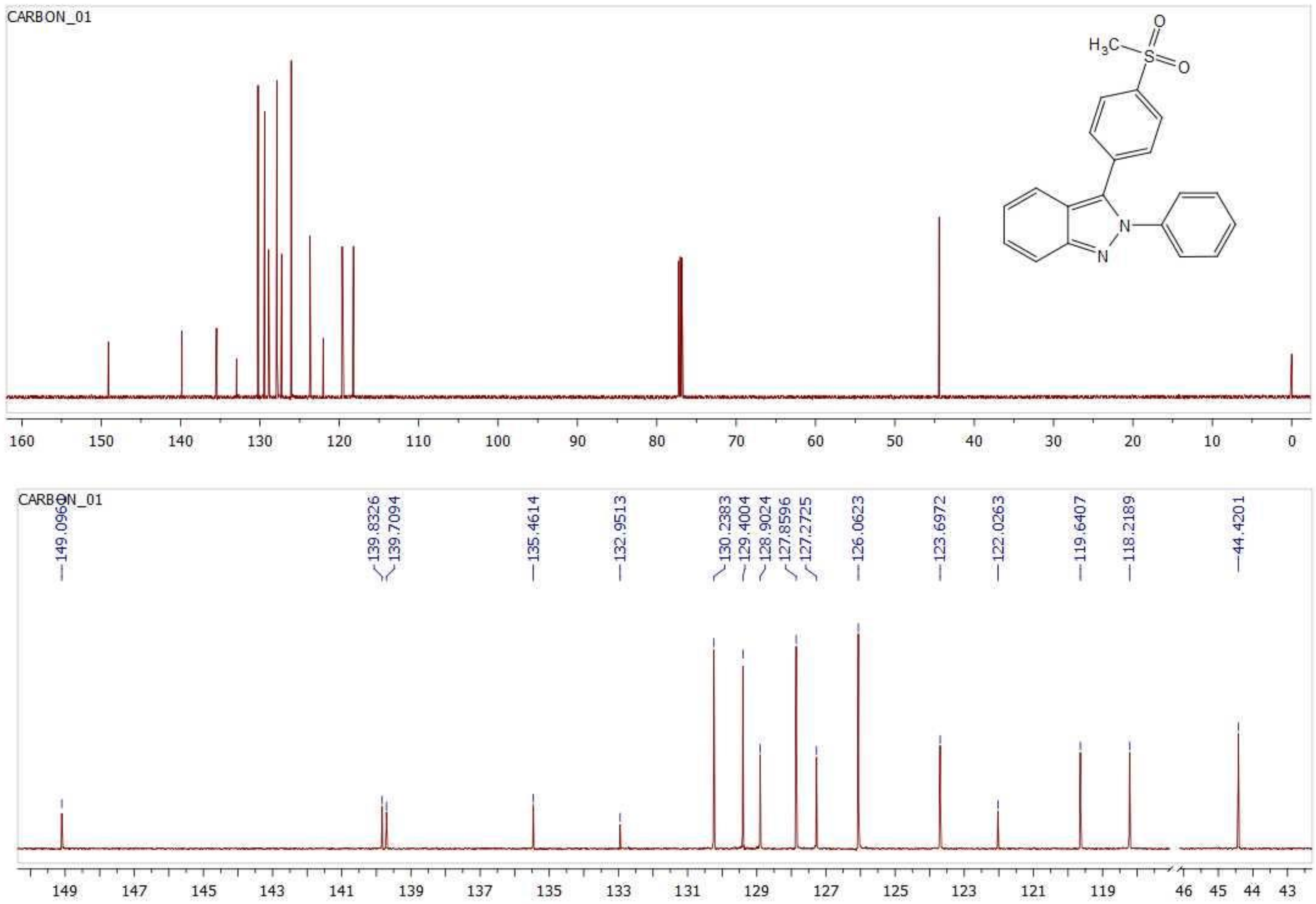

Figure S48. ^{13}C NMR (151 MHz, $CDCl_3$) for 3-(4-(methylsulfonyl)phenyl)-2-phenyl-2*H*-indazole (**26**)

Acquisition Parameter

Source Type	ESI	Ion Polarity	Positive	Set Nebulizer	0.5 Bar
Focus	Active	Set Capillary	4500 V	Set Dry Heater	150 °C
Scan Begin	50 m/z	Set End Plate Offset	-500 V	Set Dry Gas	4.0 l/min
Scan End	3000 m/z	Set Charging Voltage	0 V	Set Divert Valve	Waste
		Set Corona	0 nA	Set APCI Heater	0 °C

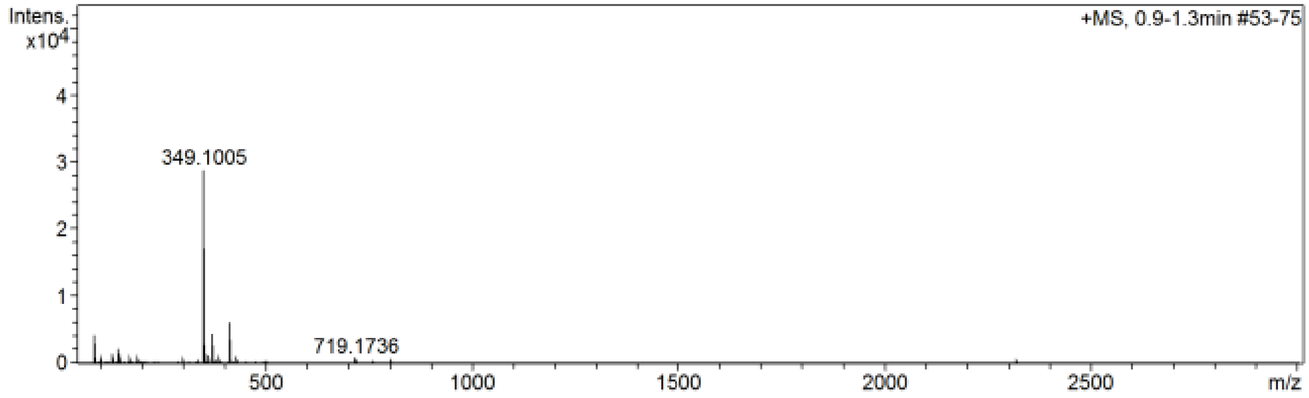

Meas. m/z	#	Ion Formula	Sum Formula	m/z	err [ppm]	mSigma	rdb	N-Rule
349.1005	1	C20H17N2O2S	C20 H16 N2 O2 S	349.1005	0.1	12.0	13.5	ok

Figure S49. MS (HR-ESI) for 3-(4-(methylsulfonyl)phenyl)-2-phenyl-2*H*-indazole (**26**)

Table S1. Antibacterial and antimycotic effect for selected compounds

Compound[a]	*E. coli* 933	*E. coli* 042	*S.* Typhi	*C. albicans*	*C. glabrata*
Zone of inhibition at 5 mg per disc (mm)					
**7**	-[b]	-	+ (9)[c]	-	-
**8**	NT[d]	NT	NT	NT	NT
**10**	-	-	-	-	-
**15**	-	-	-	-	-
**16**	-	-	-	-	-
**17**	+ (2)	-	-	-	-
**18**	-	-	-	+ (10)	+ (3)
**20**	+ (2)	-	-	-	+ (2)
**21**	-	-	-	-	-
**22**	+ (1)	-	-	-	+ (2)
**23**	-	-	-	+ (13)	+ (4)
**25**	+ (2)	-	-	+ (1)	-
**26**	-	-	-	-	-
Ciprofloxacin[e]	+	+	NT	NT	NT
Ampicillin[e]	NT	NT	+	NT	NT
Ketoconazole[e]	NT	NT	NT	+	+

[a]Indazole derivatives were tested at 5 mg.

[b] No inhibition

[c] Positive inhibition (zone of inhibition mm).

[d]NT: no tested

[e]Positive controls: ciprofloxacin 10 μg, ampicillin 25 μg and ketoconazole 50 μg.
